# Construction of High-Density Genetic Linkage Maps and Mapping of Growth-Related Quantitative Trail Loci in the Japanese Flounder (*Paralichthys olivaceus*)

**DOI:** 10.1371/journal.pone.0050404

**Published:** 2012-11-29

**Authors:** Wentao Song, Renyi Pang, Yuze Niu, Fengtao Gao, Yongwei Zhao, Jing Zhang, Jian Sun, Changwei Shao, Xiaolin Liao, Lei Wang, Yongsheng Tian, Songlin Chen

**Affiliations:** 1 Yellow Sea Fisheries Research Institute, Chinese Academy of Fishery Sciences, Qingdao, China; 2 Weihai Vocational College, Department of Biological and Chemical Engineering, Weihai, China; 3 College of Fisheries and Life Science, Shanghai Ocean University, Shanghai, China; Auburn University, United States of America

## Abstract

High-density genetic linkage maps were constructed for the Japanese flounder (Paralichthys olivaceus). A total of 1624 microsatellite markers were polymorphic in the reference family. Linkage analysis using JoinMap 4.0 resulted in the mapping of 1487 markers to 24 linkage groups, a result which was consistent with the 24 chromosomes seen in chromosome spreads. The female map was composed of 1257 markers, covering a total of 1663.8 cM with an average interval 1.35 cM between markers. The male map consisted of 1224 markers, spanning 1726.5 cM, with an average interval of 1.44 cM. The genome length in the Japanese flounder was estimated to be 1730.3 cM for the females and 1798.0 cM for the males, a coverage of 96.2% for the female and 96.0% for the male map. The mean recombination at common intervals throughout the genome revealed a slight difference between sexes, i.e. 1.07 times higher in the male than female. High-density genetic linkage maps are very useful for marker-assisted selection (MAS) programs for economically valuable traits in this species and for further evolutionary studies in flatfish and vertebrate species. Furthermore, four quantiative trait loci (QTL) associated with growth traits were mapped on the genetic map. One QTL was identified for body weight on LG 14 f, which explained 14.85% of the total variation of the body weight. Three QTL were identified for body width on LG14f and LG14m, accounting for 16.75%, 13.62% and 13.65% of the total variation in body width, respectively. The additive effects were evident as negative values. There were four QTL for growth traits clustered on LG14, which should prove to be very useful for improving growth traits using molecular MAS.

## Introduction

Genetic linkage mapping has become a critically important tool in many areas of genetic research. In order to perform a useful linkage study, it is necessary to genotype and map large numbers of the available genetic markers on the mapping families. Microsatellites comprise an excellent opportunity for genomic mapping due to their abundance in most vertebrate genomes, as well as their genomic distribution pattern, high polymorphism rate and ease of typing, all of which are determinable via PCR. Meanwhile, the simple sequence repeat (SSR) alleles are typically co-dominant, and their polymorphisms can be scored in either a simple polyacrylamide gel separation format or with high-throughput capillary arrays [1]. Genetic linkage maps based on microsatellite markers have been produced for economically important fish species, including salmon [2], tilapia [3], European sea bass [4], rainbow trout [5], sea bream [6], Barramundi [7], catfish [8], grass carp [9], Japanese flounder [10] and Asian sea bass [11].

The traditional methods of genetic improvement of quantitative traits have relied mainly on phenotype and pedigree information [12], which are both commonly influenced by environmental factors. It is generally accepted that MAS accelerates genetic improvement in a relatively short period, especially when the target characteristics are disease-related and there is a sufficient amount of observed genetic variation in a given trait. A genetic map constructed from a population segregated for a trait of interest is required for QTL identification. Information on the genetic markers associated with QTL can be used in MAS breeding programs to identify and select individuals carrying the desired traits. QTL mapping in commercial fishes is still in its infancy [13]. The QTL for growth, disease resistance and stress response have been mapped in only a few species to date, such as disease resistance in rainbow trout [14] and Japanese flounder [15], body weight in salmon [16] and European seabass [17], cold tolerance in tilapia [18], and color variation in the guppy [19].

Japanese flounder (Paralichthys olivaceus) is a marine fish which is economically important as a food, and has been widely cultured in Asian countries such as China, Japan and Korea. It is mostly distributed along the coast of China, where it has been cultured for approximately 20 years due to its favourable traits such a fast growth rate, good adaptability to temperature and disease resistance in a variety of cultivation conditions. With extensive cultivation, however, farming of the Japanese flounder has also been confronted with certain problems, including a high mortality rate as well as a decline in growth. In order to further increase the productivity of Japanese flounder farming, it is thus essential to carry out both classic selective breeding and MAS. Recently, a number of genetic studies in this species have been reported. Fuji et al. [15] found a single major genetic locus associated with lymphocystis disease resistance in the Japanese flounder and succeeded in commercially producing a lymphocystis disease-resistant strain by MAS. A large number of flounder families have been developed and disease resistance-related MHC gene markers identified in the Japanese flounder in China [20]–[22]. Furthermore, three genetic linkage maps have been published for P. olivaceus [23]–[24] [10]. In these maps, the second generation genetic linkage map of the Japanese flounder constructed by Castaño-Sánchez is the most dense, having 1375 markers. However, the male and female maps have only 235 and 184 unique positions, with average intervals of 5.0 cM and 4.4 cM, respectively. In this study, a high-density microsatellite genetic linkage map containing 1487 markers and growth-related QTL are reported for the first time for the Japanese flounder. The female and male maps have 1242 and 1215 unique positions, respectively, which afford sufficient marker density for fine mapping QTL. The significantly improved female and male maps were based on an F1 intercross derived from two heterozygous parents that resulted in segregation. The markers in these maps were distributed in 24 linkage groups, and this number is in accordance with the haploid chromosome number of the Japanese flounder. In addition, four growth rate QTLs were mapped on the genetic linkage map and some of them may be useful in MAS in future breeding programs in the Japanese flounder. These linkage maps represent a powerful tool both for research on genome evolution and for broodstock enhancement programs using MAS breeding in Japanese flounder, and will facilitate genome mapping efforts in other species of flatfish.

**Figure 1:**
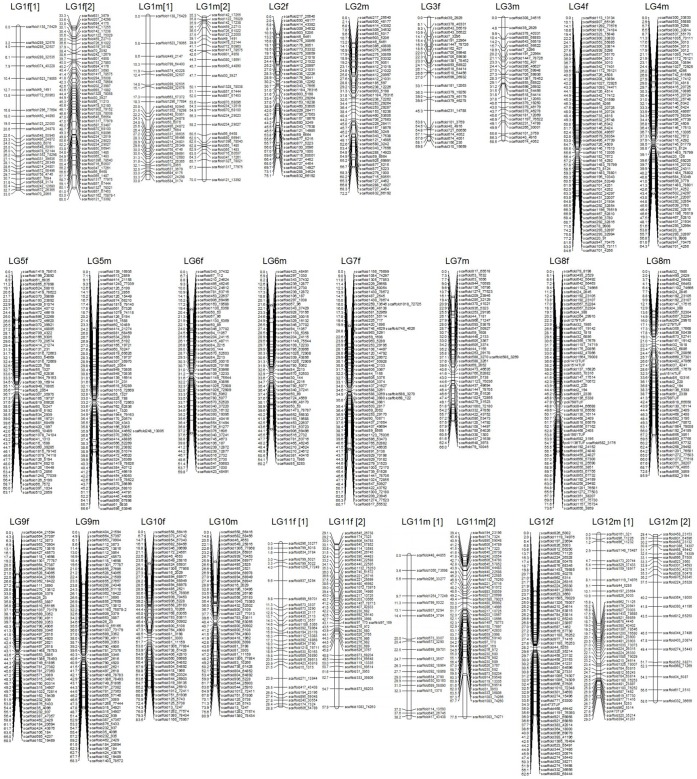
Japanese flounder male (left) and female (right) maps. Linkage groups LG1–LG12. Genetic distances in Kosambi centimorgans are listed on the left side of the linkage groups, and markers are listed on the right side of the linkage groups.

## Results

### Genetic Markers

To obtain microsatellite markers useful for linkage analysis, we examined the segregation patterns of 4600 markers in the mapping family. Among the 4600 markers, 1624 (35.3%) were polymorphic in either the male or female. The sequence data of 1587 newly identified polymorphic microsatellites were deposited with the GenBank Data Library under the accession numbers: JN900500-JN902086. A list of the 1624 polymorphic microsatellite markers is presented in a supplementary file.

**Figure 2:**
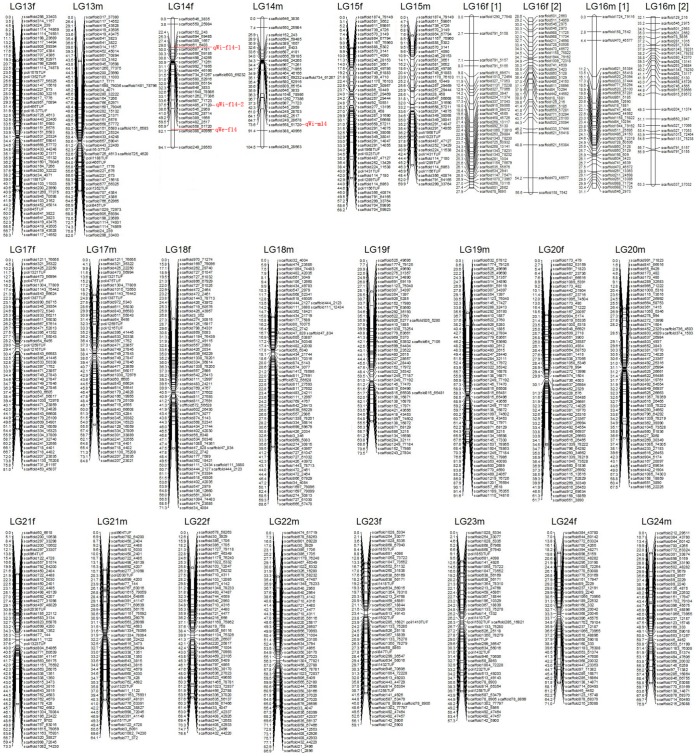
Japanese flounder male (left) and female (right) maps. Linkage groups LG13–LG24. Genetic distances in Kosambi centimorgans are listed on the left side of the linkage groups, and markers are listed on the right side of the linkage groups. QTLs are shown as body weight (qWe), body width (qWi) on LG14f and LG14m.

### Segregation Analysis

Segregation distortion from expectation under Mendelian inheritance was found in 625 (38%) of 1624 microsatellite markers. Although these markers are normally excluded from linkage analysis, the independence LOD score, one of the grouping parameters provided by JoinMap4.0, allows these markers to be included. This test for independence is not affected by segregation distortion and leads to a less spurious linkage [25]. As a result, 583 of these 625 markers were placed onto the linkage map after the linkage analysis.

**Table 1:** Summary of genetic linkage maps of Japanese flounder.

	Female maps	Male maps	Integrated map
Number of markers scored	1429	1336	1624
Number of markers mapped	1257	1224	1487
Number of unique positions	1242	1215	1466
Number of genetic linkage groups	24	24	24
Average number of markers per group	52	51	62
Minimum number of markers per group	27	27	30
Average marker spacing (cM)	1.35	1.44	1.22
Maximum length of group (cM)	94.1	104.5	101.3
Minimum length of group (cM)	44.9	54.1	50.2
Observed genome length (cM)	1663.8	1726.5	1763.3
Estimate genome length (cM)			
Ge1	1728.6	1795.6	1813.0
Ge2	1731.9	1800.5	1826.9
Ge	1730.3	1798.0	1824.4
Genome coverage %	96.2	96.0	96.7

When the total of 1624 polymorphic microsatellite markers was analyzed, 1487 markers were located on the linkage maps, containing 24 linkage groups (LGs), at a LOD score threshold value of 4.0.

**Table 2:** Test of normality for body length, weight and width.

	Mean+σ	maximum	minimum	skewness	kurtosis	p-value
body length	23.67+2.12 cm	28.0	19.3	−0.252	−0.724	0.0280
body weight	118.29+37.89 g	207.0	30.2	0.044	−0.560	0.8389
body width	8.36+0.87 cm	6.4	10.0	−0.245	−0.660	0.0706

### Sex-specific Maps

Significant linkages were identified for 1624 genetic markers. However, 137 microsatellite markers went unmapped in this analysis. Consequently, the mapping ratio of these markers is 91.6%. The female and male maps contained 1257 and 1224 markers, respectively. Both maps were found to have 24 linkage groups. The total length of the female map was determined to be 1663.8 cM, with an average interval of 1.35 cM. The linkage group size ranged from 44.9 cM to 94.1 cM. The number of loci per genetic linkage group varied from 27 to 76. The male linkage map spanned a total genetic distance of 1726.5 cM. The length of each linkage group varied from 54.1 to 104.5 cM and contained 27–66 loci per group, with an average interval of 1.44 cM. The sex-specific genetic linkage maps are presented in Figure 1 and Figure 2. The female and male maps display 1242 and 1215 unique positions, respectively. The estimated genome length, based on the two methods, were 1728.6 cM (Ge1) and 1731.9 cM [check all] (Ge2) for the female, and 1795.6 cM (Ge1) and 1800.5 cM (Ge2) for the male. The average of these two values was taken as the expected genome length, namely, 1730.3 cM for the female and 1798.0 cM for the male. A summary of the genetic linkage maps of Japanese flounder is shown in Table 1. Based on recent estimation of map length, the genomic coverage of the female and male maps were 96.2% and 96.0%, respectively.

**Figure 3:**
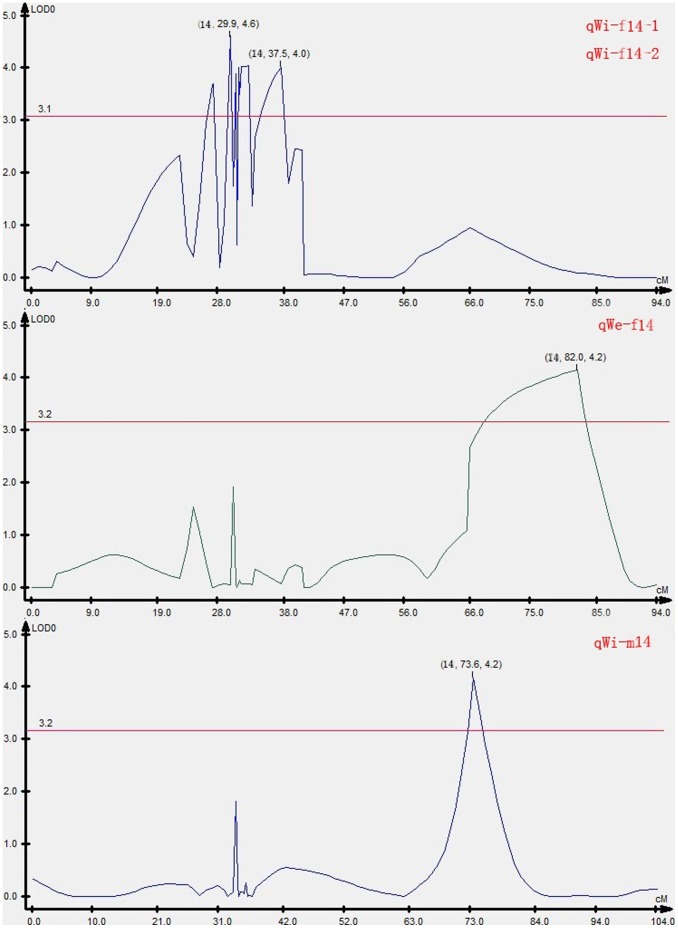
LOD curve graph of four QTLs. Abscissa indicates the relative position on the linkage groups, vertical coordinates indicates the value of LOD; the red line represents significant threshold of QTLs; the figure in “()” represents (group, position, value of LOD).

**Table 3:** Biometrical parameters of individual QTL affecting growth traits of Japanese flounder.

Trait	QTL name	LG	Marker position	Associated markers	LOD scores	QTL Interval	R^2^ (%)	Add.
Body weight	qWe-f14	14 f	82.0	scaffold388_40956	4.2	68.1–83.4	14.85	−62.22
Body width	qWi-f14-1	14 f	29.9	scaffold687_4181	4.6	29.5–30.2	16.75	−0.73
	qWi-f14-2	14 f	37.5	scaffold485_47120	4.0	34.4–38.0	13.62	−0.65
	qWi-m14	14 m	73.6	scaffold558_51720	4.2	72.6–75.1	13.63	−1.65

R^2^ (%): proportion of the explained phenotypic variance.

LG: linkage map.

Add.: the additive effects.

### Integrated Map

Either bridge markers or homologous loci were used to identify the co-linear regions in the female and male maps. The resulting integrated map contained 1487 microsatellite markers with 1466 unique positions. Twenty-four LGs were found, with an average of 62 microsatellites per LG. The total length of the map was 1763.3 cM with an average interval of 1.22 cM. The genome length of the Japanese flounder was therefore estimated to be1798.0 cM, and a coverage of 96.7% was observed. The linkage group length varied from 50.2 cM to 101.3 cM, and the number of markers on the linkage groups varied from 30 to 81.

### Differences in Recombination between the Sexes

The sexes have slight differences in recombination rate, both in general and for specific pairs of linked markers. The availability of SSR markers in the male and female maps allowed an evaluation of the respective rate of meiotic recombination. The recombination rates obtained from 24 linkage groups were on average 0.0135 in females and 0.0144 in males. Therefore, the relative recombination ratio (female-to-male; F/M) in these pairs was 1∶1.07, which was slightly higher in males than females.

### QTL Associated with Growth Traits

The result of test of normality for body length, weight and width were presented in Table 2. Four QTLs associated with growth traits were mapped in LG14 by CIM, accounting for 13.62–16.75% of the trait variation (Fig. 2 and Fig. 3). The individual QTL which were detected were as follows: One QTL that was identified for body weight, designated as qWe-f14, accounted for 14.85% of the phenotypic variation. Three QTLs for body width, designated qWi-f14-1, qWi-f14-2 and qWi-m14, were mapped in LG14 and accounted for 16.75%, 13.62% and 13.63% of the phenotypic variation, respectively. Four markers, scaffold687_4181, scaffold485_47120, scaffold558_51720 and scaffold388_40956, were highly significantly (P<0.01) correlated with growth traits (Table 3).

## Discussion

Genetic maps provide critically important genomic information and allow the exploration of QTL, which can be used to maximize the selection of target traits. The availability of a large number of genetic markers is essential for constructing a useful linkage map and for QTL mapping of genetic traits of interest. In this study, we constructed high-density microsatellite genetic linkage maps using 1624 microsatellites in the Japanese flounder. These markers will serve as an important tool for future comparative map studies and to establish the underlying correspondence with the linkage groups of other closely related species. The current maps span a total length of 1663.8 cM for the females and 1726.5 cM for the males, with 24 LGs. There were no small (doublet or triplet) linkage groups, indicating that this linkage map is complete. Only 12 of the 1624 markers studied remained unlinked to any other marker. This degree of completeness supports the utility of the genetic map as a reference tool for future genetic analysis in this species.

In humans, mice, cows and pigs, and indeed in most vertebrates studied so far, the recombination rates exhibit significant differences between the sexes. In contrast to most teleosts and mammals, but similar to previous report in this species [23], increased recombination was found in the male rather than the female. However, the previous Japanese flounder mapping cross was between a normal female and a spontaneous phenotypic male that arose during gynogenesis, and thus was a genetic XX/XX cross [23]. The observed higher recombination rate in the male in this case is not representative of a heterogametic sex pattern. In this study reported here, we used a normal male and female. The average interval between markers was slightly less for the male map (1.44 cM) than the female map (1.35 cM), suggesting that the recombination rate was slightly higher in males than in females. The recombination ratio between the male and female parents of Japanese flounder was 1.07∶1. Although it was slightly higher in males than females, the ratio was still close to 1∶1. This data suggests that the higher recombination frequency is somewhat restricted in the female of the Japanese flounder. Such restriction does not occur uniformly on all linkage groups, however, (LG2, LG3, LG4, LG5, LG9, LG12, LG13, LG15, LG18 and LG21), as the genetic map microsatellite marker distances are not proportional in male and female recombination and appear to be chromosome-specific. Differences in map length can result from a variation in the number of recombination events in the two parents as well as variations in the number and location of the mapped loci. It is common to find a difference in the recombination ratio between the two sexes in most aquatic species. For instance, the male/female recombination ratios are 1∶8.26 in Atlantic salmon [26], 1∶3.25 in rainbow trout [27], 1∶1.3 in turbot [28], 1∶2 in halibut [29], 1∶1.2 in Sparus aurata [30] and 0.7∶1 in the Japanese flounder [10], which all show a higher recombination in females. In contrast, Coimbra et al. [23] reported a higher recombination ratio of female/male of that was 1∶7.4. Nevertheless, despite this being a common phenomenon, the mechanism responsible for the different recombination rates between the genders is still not well understood. Some studies have shown that recombination rate differences are associated with QTL [31]. Selection using linked markers is more efficient when recombination does not occur between the markers and the QTL loci. Reid et al. hypothesized that the sex-specific environment in which the germ cells undergo meiosis may be the reason for the differences in recombination. In medaka, sex-reversed XY females have recombination patterns that are very similar to XX females [32], suggesting that sex indeed determines the recombination pattern and rate. The average recombination rate across all of the linkage groups is approximately 0.012 in the Japanese flounder, which is lower than that in zebrafish [33], tilapia [3], catfish [8], grass carp [9], rainbow trout [34], turbot [28] and Asian sea bass [11].

In the mapping family, segregation distortion was observed for 625 markers and the distortion rate was approximately 38%. A higher distortion rate has been reported in previous studies, such as 40.5% for the Pacific white shrimp [35]. For other marine species, the rate is 26.9% in the Pacific oyster [36], 13.3% in the rainbow trout [37], 16% in the channel catfish [38], 16.3% in the common carp [39] and 2.28% in the bluegill sunfish [40]. The reasons for the distortion of the segregation ratios may be due to factors such as genetic isolation [41], sampling errors [42], scoring errors [43], the progeny population size and amplification of a single-sized fragment derived from several different genomic regions [44]. Additionally, lethal effects resulting from a recessive homozygote in the juvenile period may affect distorted segregation. Hubert et al. [45] selected juveniles for linkage analysis to eliminate effects resulting from segregating distortion in older progeny.

Information on genetic markers associated with quantitative trait loci (QTL) can be used in breeding programs to identify and select individuals carrying desired traits. In a previous study, one possible QTL was detected associated with resistance to lymphocystis disease [15]. In this work, sex-specific linkage maps were used for QTL analysis. In total, four QTLs associated with growth traits were detected. One QTL was identified for body weight on LG 14 f. Three QTLs were identified for body width on the LG14f and LG14m. The additive effects were negative values. To improve the utility of the QTL in MAS and also move toward the positional cloning of candidate genes, fine mapping of the QTL to a more restricted chromosomal region is necessary [46]. Although QTL mapping has been conducted in a few fish species, such as rainbow trout [14], salmon [16], European seabass [17], tilapia [18] and the guppy [19], the region is usually longer than 10 cM. In this study, the QTL intervals were 0.7, 3.6 and 2.5 cM for body width, and 15.3 cM for body weight. Moreover, there were four QTL for growth traits clustered on one linkage map (LG14), which will likely prove to be very useful for improving growth traits by molecular MAS.

The flatfishes (Pleuronectiformes), which include flounder, plaice, sole, turbot and halibut, are a broad taxonomic group comprising 11 families and approximately 500 species worldwide. And they are a group of teleosts having considerable commercial and environmental interest in terms of both fisheries and aquaculture. The increase in the genome sequencing data has allowed the construction of genetic linkage maps in a variety of flatfish species, such as the Japanese flounder [23]–[24]10], turbot [28], Atlantic halibut [29], barfin flounder and spotted halibut [47] and the half-smooth tongue sole [48]. These maps are invaluable for investigating genomic organization and identifying the genetic traits of commercial interest.

The first genetic linkage map of the Japanese flounder was reported by Coimbra et al. [23] using 111 microsatellite markers and 352 AFLP fragments. The parental male linkage map consisted of 25 linkage groups, while the female map consisted of 27 groups, with an average resolution of 8 and 6.6 cM, respectively. The total map length was estimated to be approximately 1000–1200 cM. Recombination rates were higher in male flounder compared to the female (7.4∶1). Kang et al. [24] constructed a genetic microsatellite linkage map for the Japanese flounder based on 180 microsatellites and 31 expressed sequence tag(EST) derived markers. The total map distance was 1,001.3 cM with 24 linkage groups. Castaño-Sánchez et al. [10] constructed a second generation genetic linkage map of the Japanese flounder. This Japanese flounder map displays the densest flatfish linkage map, including 1375 markers. The male and female maps have 235 and 184 unique positions, with average intervals of 5.0 cM and 4.4 cM, respectively. Therefore, high density SSR genetic linkage maps are urgently needed for fine QTL mapping and MAS in the flounder.

The present study developed a high-density genetic linkage map for Japanese flounder. The improved linkage maps consisted of 1487 markers. The female and male maps have 1242 and 1215 unique positions, respectively, which afford sufficient marker density for fine mapping of the QTL. Meanwhile, mapping the QTL for growth traits (body weight and body width) in the Japanese flounder will provide a basis for identifying marker for growth-related genes and provide a powerful tool for building a foundation for genetic improvement programs using MAS.

The Japanese flounder linkage map provides a basis for a thorough exploration of the entire genome, and for identifying genomic regions related to productivity and of evolutionary interest in the Japanese flounder. Since QTL are often conserved across closely related species [16], QTL studies in the Japanese flounder may be applicable to other flatfishes. In the meantime, this linkage map will form the basis for enhancing Japanese flounder broodstock production through MAS.

## Materials and Methods

### Ethics Statement

All the experimental animal programs involved in this study were approved by the Yellow Sea Fisheries Research Institute’s animal care and use committee, and followed the experimental basic principles. A slight fin tissue from the parents and F1 offspring was sheared under MS222 anesthesia, and all efforts were made to minimize suffering.

### Mapping Family

In May 2011, a full-sib family of Japanese flounder was constructed and used for the development of a genetic linkage map. The male parent was selected from a group of fish derived from wild populations. The female parentwas selected from a cultured population. The induction of the maturation of broodstock and artificial fertilization of sperm and eggs were carried out as described previously [20]. The mapping family was raised in factitial aquarium at the HuangHai Aquaculture Company (Yantai, China). In November 2011, the F1 offspring had shown apparent disparity in the growth-related characters (Table 2). Eighty individuals from the mapping family were collected randomly. Growth-related characters, such as body length, body weight and body width, were measured and recorded. A slight fin tissue from the parents and F1 offspring was sheared and stored in absolute ethanol until DNA extraction. The genomic DNA of the two parents and progeny was extracted following phenol/chloroform procedures with RNase treatment [49].

### Microsatellite Markers

A total of 4600 Japanese flounder Microsatellite markers were tested for segregation across a set of eight individual progeny. These microsatellite markers were recruited from two sources: The first set of 4000 microsatellite markers was obtained from different scaffold by genome sequencing of Japanese flounder in our laboratory. (2) The remaining 600 markers had been previously reported in this species by Castaño-Sánchez et al. [10].

### Genotyping

The primers flanking the microsatellite regions were designed using Primer 5. All primers were designed for a 57.5°C annealing temperature, a 100–300 bp total amplification product size and 40–60% GC content. All of the microsatellite markers were used to genotype two parents and six progeny for screening the segregation markers in the mapping population. The microsatellite markers that produced polymorphic fragments were used in the subsequent genotyping of the parents and 92 progeny to construct the linkage maps. Amplifications were performed in an ABI Veriti 96 well thermal cycler, BIO-RAD MyCycler thermal cycler and Fisher Scientific LabServ LS-P96G thermal cycler. The PCR amplifications were performed under the following conditions: 95°C for 5 min, followed by 32 cycles at 95°C for 30 s, a specific annealing temperature of a specific primer pair for 30 s and 72°C for 30 s, and the final extension was 72°C for 10 min. Amplification reactions were carried out on a 15-µl column consisting of 10× Taq buffer, 0.5 U Taq polymerase, 0.6 mM dNTP (+MgCl_2_), 0.6 µM of each primer and 10–30 ng template DNA. The final volume was adjusted with sterile distilled water. The PCR products were separated on 8% polyacrylamide gels (PAGE) and visualized by silver staining [50].

### Linkage Analysis

Genetic marker data were scored according to the definition of JoinMap 4.0. All of the statistical analyses described below were performed using the same software with a cross-pollinating (CP) type population, which is designed to handle F1 outbreeding population data containing various genotype configurations. Pairwise analyses were performed and markers were sorted in linkage groups at a minimum LOD score of 4.0. The “locus genotype frequency” function calculated chi-square values for each marker to test for the expected Mendelian segregation ratio. Markers significantly deviating from the expected ratio (p<0.05) were eliminated. The linkage distances were estimated for each LG assuming the Kosambi mapping function. Linkage groups with genetic markers on individual maps were merged to create an integrated map using the “Join-combine groups for map integration” command.

### Genome Size and Coverage

The estimated genome length (Ge) of the consensus female and male genomes was estimated using two different methods. First, Genome estimation size 1 (Ge1) was calculated by adding 2 s to the length of each genetic linkage group to account for the chromosome ends, where s represents the average spacing of the genetic linkage map. Genome estimation size 2 (Ge2) was calculated by multiplying the length of each genetic linkage group by (m+1)/(m−1), where m is the number of loci in each genetic linkage group. The estimated genome size (Ge) for each sex was taken as the average of the two estimates. The observed genome length was taken as the length of the framework map (Gof) and the total length (Goa) considering all the markers on the framework map, including the triplets and doublets. The map coverage ratios, Gof and Goa, were calculated as Gof/Ge and Goa/Ge, respectively.

### QTL Analysis

QTL analysis was performed with WinQTLCart2.5 software using the composite interval mapping (CIM) method (model 6). Unlinked might act as an additional environmental effect that reduces the significance of the estimated marker-trait association. Therefore, CIM includes neighboring markers and uses the remaining background markers as cofactors in order to remove the effects of multiple QTL. While the CIM analysis was conducted separately for each map, the background markers used in these analyses were derived from both maps. Five background markers were employed in CIM analysis. A stringent LOD score threshold ≥2.5 was set to identify the putative presence of QTL related to growth traits.

## Supporting Information

Table S1 **Characterization of microsatellite markers genotyped in Japanese flounder mapping family.**
(DOCX)
