# Correction: Construction of High-Density Genetic Linkage Maps and Mapping of Growth-Related Quantitative Trail Loci in the Japanese Flounder (*Paralichthys olivaceus*)

**DOI:** 10.1371/annotation/46b9426e-29cc-4fae-aabc-77fa2a7febec

**Published:** 2013-05-30

**Authors:** Wentao Song, Renyi Pang, Yuze Niu, Fengtao Gao, Yongwei Zhao, Jing Zhang, Jian Sun, Changwei Shao, Xiaolin Liao, Lei Wang, Yongsheng Tian, Songlin Chen

The Funding Statement contains an incorrect grant number. The following is the correct Funding Statement:

The work was supported by grants from the National Major Basic Research Program (2010CB126303), the State 863 High-Technology R&D Project (2012AA10A408; 2012AA092203) of China, and the Taishan Scholar Project of Shandong Province. The funders had an important role in study design and the decision to publish. 

