# Supplementary material for: Construction of High-Density Genetic Linkage Maps and Mapping of Growth-Related Quantitative Trail Loci in the Japanese Flounder (Paralichthys olivaceus)
Source: PLoS One. 2012 Nov 29;7(11):e50404. doi: 10.1371/journal.pone.0050404 (PMC3510152; doi:10.1371/journal.pone.0050404)
Supplement: Table S1 — Characterization of microsatellite markers genotyped in Japanese flounder mapping family. (DOCX) [file pone.0050404.s001.docx]

Additional file:

Table 3 Characterization of microsatellite markers genotyped in this mapping family

| Locus | GenBank Accession no. | Primer sequences (5’-3’) | *Tm* (℃) |
| --- | --- | --- | --- |
| scaffold1138_75429 | JN900500 | F: CAAAACAAGACAGGCCACA  R: ACCAAGTTCAGAGGAAGTGTC | 55 |
| scaffold289_32576 | JN900501 | F: CAATGACTCGCAATATTGATTTG  R: GTTCAGCCCATAAGGACT | 57.5 |
| scaffold289_32507 | JN900502 | F: ACCTTTACCTGGCCGATTAGAG  R: TCAATGCAGTCTGTCTTAAGTGACA | 57 |
| scaffold289_32535 | JN900503 | F: GTGAGCCATCATACTGTGACCA  R: GCTGAGGCACACACTGAC | 57.5 |
| scaffold374_40228 | JN900504 | F: GTTCAACCTCCAGTGTCAGC  R: GTCCAGCATACTTGTGGCTAC | 57.5 |
| scaffold1623_79065 | JN900505 | F: GTGGGATAACACTCATCACCAACA  R: TGTTCAGTTTTGACTCAGTGCCTC | 57.5 |
| scaffold49_1491 | JN900506 | F: CAACTCTGATACGTACGCTGCT  R: GCAATGTTCCTGGTGAGTTCAG | 57.5 |
| scaffold772_60963 | JN900507 | F: GAAAAAGTCCTAAAGAGGTAACAA  R: ACATTTTCTATTGGTTTAAGTCTCC | 52 |
| scaffold450_44890 | JN900508 | F: CAGGAACAAGACCGAGGAG  R: GCAGAATCTGTTCAATGTGATGG | 56 |
| scaffold123_22000 | JN900509 | F: GAATTGAGCCTGCCACAGTTAG  R: GCAAAGTATTCTCTCCTGCGTG | 57.5 |
| scaffold548_50945 | JN900510 | F: TGTTTAGGTGCACAGGTGATCAGT  R: TGTGGGACCATTTTTATTGACCTC | 57.5 |
| scaffold495_24878 | JN900511 | F: TCAACACTGACAATGCTCAGGAG  R: ACACATCATCAACAGCACTGGAA | 57.5 |
| scaffold50_6076 | JN900512 | F: CCTCACAATGATGGCATTGAACC  R: AGCCTAATGGTCAGCTGTGTT | 57.5 |
| scaffold495_24872 | JN900513 | F: CATCATGGTTTTCAGCACCTCC  R: CCAACATCTCCATTGCAGTCATG | 57.5 |
| scaffold548_50951 | JN900514 | F: AACAAACATATTGAGGACATGAACG  R: TCAAATACTAGAGGGGGTGTAGCC | 57.5 |
| scaffold478_52144 | JN900515 | F: TGCAACTGCATGAATAAGGATTTTT  R: AGTCCCGCTGGGTAGGACAC | 57.5 |
| scaffold1352_78298 | JN900516 | F: CAACCAAGGCAGGATTACACAAAC  R: GAGCAGCCTTGTGTCTTTGATTTT | 57.5 |
| scaffold331_39635 | JN900517 | F: AGTTTGAGGTTGACTGCAGAGTGA  R: CCCCATGTTAACAGATATAGTTTCCC | 57.5 |
| scaffold572_26349 | JN900518 | F:GGTCAAACGCTCATCCAGG  R:TTATGACCTATACTGCAGGCAGC | 57.5 |
| scaffold214_24801 | JN900519 | F:CCTGAGCATGACACTTGCAG  R:GTTTCTGTCAGGTGCGTGAG | 57.5 |
| scaffold572_56496 | JN900520 | F:TAAACAGGAAACAGAGGCATTCAG  R:CACATGAGGGGAGTCCTCACT | 57.5 |
| scaffold758_4746 | JN900521 | F:CAACAGACGAGACACATGCTG  R:CAGTGCACCTCTTGGGTG | 57.5 |
| scaffold67_7694 | JN900522 | F:TCACAGCCATGATGAGGAGTTTC  R:CATCAACTGAGGGATGTCGCTA | 57.5 |
| scaffold242_12680 | JN900523 | F:CGTATGTGTGCTGTTGGGG  R:CCGAACTTTCCCTCTGGATCA | 57.5 |
| scaffold572_26365 | JN900524 | F:CCGATGCAGCACTTGTTAAGG  R:TACCAATGTCTCTGGTGGGAAAG | 57.5 |
| scaffold70_2065 | JN900525 | F:TAGAGTGTCCATCAAAGCCAGACA  R:AGAACTGAGCCTCTGCTTCCTCTC | 57.5 |
| scaffold631_3679 | JN900526 | F:CACACAAACATACCACTCCACACA  R:AAGAACAGCCATTTTCCTCCTGTT | 57.5 |
| scaffold207_24298 | JN900527 | F:TTAACTGCTCAGTGGGAACTGG  R:CCTTTCTATGGATCTGGGTCCG | 57.5 |
| scaffold684_4175 | JN900528 | F:AGCAGCATTGAGCAAATGTGTC  R:TGTCAGCTGGGATTAGCTTCAG | 57.5 |
| scaffold584_3174 | JN900529 | F:TCACCTGTAAACATCAGAGGGACA  R:TAGCATTTATTTTCCTCCCACAGC | 57.5 |
| scaffold142_17255 | JN900530 | F:GAGTCATAGATGGGAACCTGCA  R:TGATATGTGGCATCCTGGCA | 57.5 |
| scaffold142_17238 | JN900531 | F:CCATCACCTCCTCTGGAAACA  R:TCTCCTGCTCACACTGGTC | 57.5 |
| scaffold1122_75029 | JN900532 | F:CCCATCGTCCTGACAAACTTC  R:ACAAAACCCAGCCAACTGTC | 57.5 |
| scaffold459_22713 | JN900533 | F:AATTCACCTGAGATGGAGCCTG  R:GATTGTGTTTCCCTGTCAAGCG | 57.5 |
| scaffold726_61022 | JN900534 | F:CATCCAGTCCTGTGCAAAAGTTC  R:CCGGGTCAGTAGTAGGATGG | 57.5 |
| scaffold319_35182 | JN900535 | F:TGAAATGCTGCCAAGGAGC  R:GGTCATGATACACCGTGTGC | 57.5 |
| scaffold70_2092 | JN900536 | F:CTCCAGTTATAGCCATGGCCT  R:AACATTCATCCAAGTGTGCTGC | 57.5 |
| scaffold242_12679 | JN900537 | F:CCCTTGTTGGTTACTGCTCAGA  R:ACACTTGGAATCAGGGTGAGG | 57.5 |
| scaffold753_27863 | JN900538 | F:CAGCTCTGCAACCAGATGG  R:TGCACTTGGATATCAGTGTGAGG | 57.5 |
| scaffold684_4176 | JN900539 | F:CATGCAAAGTTGTAAAAATGAGCTGT  R:AGTTTGAGGGAGGAGGAGAGAAGA | 57.5 |
| scaffold631_3690 | JN900540 | F:CCTGAGAGGCTTAATCTATGGGG  R:TATCTGCTTTCAGGTACCCTGC | 57.5 |
| scaffold801_4858 | JN900541 | F:ACCACGTCTGTTTACGCTACATG  R:GTTCACGTTTGTCCATGTGAGTG | 57.5 |
| scaffold294_35008 | JN900542 | F:GGAGTGAGACATGAGATCGAGGTG  R:CCTGCATCCACCCAACTGTG | 57.5 |
| scaffold1432_78684 | JN900543 | F:TCTGTGGAGGCTGTGTCTC  R:ACAGAGATGGAATCCCTGTTTGAG | 57.5 |
| scaffold1411_78575 | JN900544 | F:CTGGAGGTAGTCCAGAGCC  R:GGTTCAGAGATGTTGTGGCC | 57.5 |
| scaffold993_71882 | JN900545 | F:CACTTGTCCAAAATGTTTATTCCCTT  R:GTCAGATGCATCCTGTCAAAACTG | 57.5 |
| scaffold202_23987 | JN900546 | F:TTGATCAAAGCATGACAATGTGC  R:AGCCATACACCTGTCAGAAGCAAT | 57.5 |
| scaffold30_3927 | JN900547 | F:TGTGAGCAGATAAACACAACGTGA  R:CAGGGGATGTGTGTCTTGACAGTA | 57.5 |
| scaffold1328_78058 | JN900548 | F:AGGCAGAGAGAGGTGTTCG  R:CGACGTGAGTCACACATCCT | 57.5 |
| scaffold95_11213 | JN900549 | F:CTTAGTCGGTCACAACTGGC  R:TGCCAACTAGTACAGACTGCTTC | 57.5 |
| scaffold478_52180 | JN900550 | F:AGTCTGCTTCATCACTGGGACTCT  R:AGTGCATCCCAAATGTTGTATCCT | 57.5 |
| scaffold1328_78038 | JN900551 | F:CTGGATCCCAAATGCTGTTCC  R:CTGGTTCTGTCGTGGATTGC | 57.5 |
| scaffold1902_79152 | JN900552 | F:CAGGCCTACAGCTTCCTAACA  R:GGGTCTGAACTCCAGCTGAT | 57.5 |
| scaffold645_56554 | JN900553 | F:CCTCGTAAACACTGCACCTC  R:CACACAGTGGAAACAGCAGTAG | 57 |
| scaffold1317_77979 | JN900554 | F:AATGGATTTTTCTTCCTCCTCTGC  R:CAAACATCAAAATGGTGGAACAGA | 57.5 |
| scaffold857_67453 | JN900555 | F:CAACCTTGAGTCAAACTGGACC  R:GCTCTGCAGGGGAGTTATGT | 57.5 |
| scaffold587_53487 | JN900556 | F:GAACTGGCCAACGTGTCC  R:TCCATTGATCTAAGCTGTCATCTGTG | 57.5 |
| scaffold870_68100 | JN900557 | F:CTGCTGGTTTCAACAGTGGG  R:TGCCAATTTCCATGAGAAAGGC | 57.5 |
| scaffold234_12519 | JN900558 | F:AAACGGTGGATTTGAACACGC  R:TCTGATGCAGAACGGAGGAAG | 57.5 |
| scaffold870_68096 | JN900559 | F:CATTAGCATTGTTTGGACAGAGAGC  R:ACACAGGGTCAGACAGACATTC | 57.5 |
| scaffold234_29023 | JN900560 | F:GATCATTTCTCCTCATGTCAAGTCC  R:AGATATCGTGGAAATCTGCGAGAG | 57.5 |
| scaffold850_66941 | JN900561 | F:TGTTTGAGAACACGGGAGTGA  R:GCCAGTTTCAGCGTTCCA | 57.5 |
| scaffold234_29027 | JN900562 | F:TCACGTCACACAGAAGATGTCAGA  R:GCTCACTGGCCCCTTTAAACAT | 57.5 |
| scaffold365_1487 | JN900563 | F:ATCCGATCAGCCAGTGTAGG  R:TGCAGAGAACCAGACTTCCAG | 57.5 |
| scaffold716_60507 | JN900564 | F:ACTTGCTGAATCCTCAGCCT  R:GAGCCTATCGACAACCAACAC | 57.5 |
| scaffold347_17261 | JN900565 | F:GTTGAAGTGAACAGCGATGATGTC  R:GAGAACACTCTGCTGGGTGA | 57.5 |
| scaffold1396_78540 | JN900566 | F:GATGGCGGAAGACGAGACT  R:CAGGTTTCTGATTGGCTGCTAC | 57.5 |
| scaffold365_1483 | JN900567 | F:ACAGGTTCCTCGTTACAGCTC  R:GGACGGTAAAGACGTCCAAATG | 57.5 |
| scaffold365_1485 | JN900568 | F:GTAAATCACCGTGACAAGTCCG  R:AAACTCTGGAGAAAGTCCAGACC | 57.5 |
| scaffold56_6468 | JN900569 | F:CGGTCTTTGTCTGGCTCAG  R:CACGTATGTTGAGGATCATGTTTGAC | 57.5 |
| scaffold1327_78021 | JN900570 | F:CATGTGTGGTTACATGTGTCTGGA  R:GGTCTTTAGTTCAGGATCCCATCA | 57.5 |
| scaffold857_67444 | JN900571 | F:CTGACGACTAAAATAACCGCCAAC  R:CCGTCTATTTCTGTGGACGTCTG | 57.5 |
| scaffold1317_77975 | JN900572 | F:GTTTCTCCTGATGTCCACATCCTT  R:CTGCCACTCATCACATCTGCTTAT | 57.5 |
| scaffold1296_77694 | JN900573 | F:AGCGGAGCGATTACTGCT  R:GCCAGGGAGCTATTTTTGGAG | 57.5 |
| scaffold1162_75878 | JN900574 | F:TTACTTCAGCGTCCATCAAGTCAG  R:TCAGGTGTGCATCCTTG | 57.5 |
| scaffold121_13392 | JN900575 | F:GGTCGCAGAAGATAAATCACAGGT  R:AGGGTCAGCTCATCAAGCAGAG | 57.5 |
| scaffold217_25540 | JN900576 | F:CTCTGAATGTGGTAACAGCAGC  R:TCCAAAAACCAATCAGCCAGC | 57.5 |
| scaffold500_48177 | JN900577 | F:TCTTCATCATCTGCACAACAACAA  R:GCTCGTCAGAATGAAACTAGCGTT | 57.5 |
| scaffold309_34532 | JN900578 | F:TCTACGCCCTGAAGAGGTC  R:CGTCCACAACCACGACTC | 57.5 |
| scaffold903_5206 | JN900579 | F:AATCAGCACAGCGTTCACC  R:GAGATGATTGCAACGCTGTCTG | 57.5 |
| scaffold46_5461 | JN900580 | F:CAGATCTGCAGCATCACCAC  R:GCCATAGTAACAACATGAGGGC | 57.5 |
| scaffold275_30865 | JN900581 | F:CACAAACAGACAGCTGCGTTAC  R:AGGTGATGTCTGGATCCGTC | 57.5 |
| scaffold275_30859 | JN900582 | F:CTCCACAGAGTGAGATCCAGAC  R:GATGACGTTGTGGTTTACGCC | 57.5 |
| scaffold176_9051 | JN900583 | F:GGAAATAAAACGACTCCGCTGG  R:TCAGTTCTTCACTCTGTGCCG | 57.5 |
| scaffold773_63332 | JN900584 | F:ATGACCCGGAGAACACTGAG  R:CATGGTCACTTCCTGCTGTG | 57.5 |
| scaffold412_21018 | JN900585 | F:AACATTGAAAGCACCGTGTCG  R:GACCATAGAGGCATCCGGT | 57.5 |
| scaffold412_21022 | JN900586 | F:CCTATACATGACGGTGAAGCTGT  R:TGTTGAACACAGGGACGAGTT | 57.5 |
| scaffold412_20997 | JN900587 | F:CCTCAGTAGTTGTACTCATGGAGC  R:CTGGTTCAACACGTCACACAC | 57.5 |
| scaffold412_21031 | JN900588 | F:TGAGTGTTTCGGTCCACTTCAG  R:ATTCATGCCAGGGCACAAAC | 57.5 |
| scaffold238_12226 | JN900589 | F: ACAGCTCCTACAACGCC  R:CCTGTTCTGCAAAGACCATCAC | 57.5 |
| scaffold176_9041 | JN900590 | F:CTGTTGAACCTCAGACGTCGA  R:CCAACATGCTCCTGAGTGGTA | 57.5 |
| scaffold238_12262 | JN900591 | F:CAGGGAAGAGCTTTAATATTCCAACA  R:ATGTGATTCTCTCCCCGTGTTTC | 57.5 |
| scaffold343_17655 | JN900592 | F:CAGACCTCAGCTCAATGTGC  R:ATCAGAGGAGTCCACCTGC | 57.5 |
| scaffold1184_76316 | JN900593 | F:TGCAGCTTAGGACTCCACTG  R:TTGAGAGCTCGATAGCAGGC | 57.5 |
| scaffold903_5198 | JN900594 | F:AAGTGAAGCTCTACGACGGAAG  R:GGGATGAAAGAGCTCAGGAGT | 57.5 |
| scaffold253_29264 | JN900595 | F:CAAAACTGGAGACGGGGAGA  R:TTGATATCTGTTCCCAGCAGCAG | 57.5 |
| scaffold153_18238 | JN900596 | F:GTGGTTGACTTTCGTCCTTCC  R:TTCACAGGCTGGAGACACAA | 57.5 |
| scaffold460_53605 | JN900597 | F:GCTTACACAATGAAAACACATCCC  R:CACTTTGGTTAATAAATGACCGTTGA | 57.5 |
| scaffold229_28411 | JN900598 | F:TGCCTTGAAATCCTAGCTTTGTTC  R:GAAATGTGTTGAATCAGCAAGGG | 57.5 |
| scaffold706_27563 | JN900599 | F:ACACGCACAAACACTGACAC  R:CTGATTTAAGTCTGTGCGCTGG | 57.5 |
| scaffold157_18676 | JN900600 | F:TCAGGTGATTCTACAACGTCCAG  R:CTGCTGTCAGTGGATTACATGC | 57.5 |
| scaffold580_3249 | JN900601 | F:ATGAATTTGGAAACACGCTTC  R:GTGATTCTGCATTTCACTGGACAC | 57.5 |
| scaffold343_17538 | JN900602 | F:AGAAGGATTGATGGAAACCTGTGT  R:GGGAATAATTCATCCCACTATCCA | 57.5 |
| scaffold121_14985 | JN900603 | F:GCGAGGTTGTCTCCTTTCTC  R:CACGACTCAATTTAGGCTCCAGAT | 57.5 |
| scaffold59_6564 | JN900604 | F:TCCTGGAATGTACGGGAACAG  R:GGAGGAAGTAGAGAAAGGCAGT | 57.5 |
| scaffold877_5214 | JN900605 | F:TGCTCATAAACATATCACTAACCCACA  R:TGTGAGGTCCAAGCACTTACACAC | 57.5 |
| scaffold877_5215 | JN900606 | F:TAAGTGTCAGTAAAGTTGGGGGCA  R:CTGTTTGATTATTGGCCCAGTCAG | 57.5 |
| scaffold877_5223 | JN900607 | F:ACGTGTTTTGAAGCCCAAAATG  R:CAATTTCCCAACTGGGATC | 57.5 |
| scaffold100_3596 | JN900608 | F:TGCTGTCCCGGTTAGTCTG  R:GGACCCAAATACGTCCTGAATGT | 57.5 |
| scaffold279_1000 | JN900609 | F:GTTAGGGCCTTTTTGATTTGACAC  R:GTCGTCAGAAGTCATTGAGACACA | 57.5 |
| scaffold715_60551 | JN900610 | F:GTATACACACAGGTGGGAACGGT  R:AGTCATGATAGTCATGTGCGTTTG | 57.5 |
| scaffold727_4462 | JN900611 | F:GCAAGATCAACTTCCCCAGAC  R:AGGTAGTCACCAGCCACAG | 57.5 |
| scaffold727_4454 | JN900612 | F:TCTAGTCAGCAGCCCTTCAG  R:GCCTTGGAAGATAACATGACAGC | 57.5 |
| scaffold288_14927 | JN900613 | F:AGTGGATTAGCGTGGTGGATAC  R:AGATCAGTGTGGTCAAGGTGG | 57.5 |
| scaffold288_34524 | JN900614 | F:AACACAGCAGCGGATACAG  R:ACTTATGCGTCGTTTTGTG | 57.5 |
| scaffold378_40331 | JN900615 | F:GACCTGCAGTGTAGCAGC  R:GTGAATCTGGAATTTCTTACGCACAC | 57.5 |
| scaffold643_56519 | JN900616 | F:AGGCTCCTCCTGTCAGTG  R:GCTCGGCCAGATTTAAGACC | 57.5 |
| scaffold643_56522 | JN900617 | F:TGTCAACATGGACTTCTCAGCA  R:AGTGCAATCAAATAAGTTTCCCCTCA | 57.5 |
| scaffold7_3296 | JN900618 | F:TACTGGGAATAACGAAGGCAGAGA  R:ATTGGACCGCGACTATACTGTAGG | 57.5 |
| scaffold191_22964 | JN900619 | F:GAAAGTGAGGGAACGAAGAGG  R:TGTTGTCCAGCTCTGTAAACCAAC | 57.5 |
| scaffold1447_78726 | JN900620 | F:CCGCTGACTTTGATAACTCTGC  R:GTCAGCATCTGTGAGCTTCATC | 57.5 |
| scaffold162_407 | JN900621 | F:TCACTTCAACCATGTTGTCGGT  R:ATGATGTCCTACCTCAAACAGCC | 57.5 |
| scaffold705_59948 | JN900622 | F:CACACTCAAACATCAGACGAGACA  R:GACGATGATGTGATGTATCAGGGA | 57.5 |
| scaffold705_59936 | JN900623 | F:TCCTCCTCTCTTTGACTTGCAGAC  R:CAGTAACTAGACATCATCACGCCG | 57.5 |
| scaffold1381_78452 | JN900624 | F:GAGAGTTGACCAGAGGGGTATG  R:TGTATCTGCTGGTGAGGCTG | 57.5 |
| scaffold518_54414 | JN900625 | F:TCACTGACCCTAATCACCTCTCAGT  R:GACAGTTTTACACAACGCAAGCC | 57.5 |
| scaffold518_54456 | JN900626 | F:ACCTTTCCATCTCTTCTTCTGCG  R:CCTGTTACCTCATCTCTGTTATCTGCT | 57.5 |
| scaffold599_26932 | JN900627 | F:CAGACCAGCTCCAGAGAGAC  R:CAGGTGGACTTCAAAGCCG | 57.5 |
| scaffold181_12063 | JN900628 | F:GGCATATCCTCTGCACGGTA  R:CATTGGCATTGGAGATGAGCC | 57.5 |
| scaffold379_19250 | JN900629 | F:ACACGAGCTGCTATGACGAC  R:ACCCTGGTTACTCTTGGGTTG | 57.5 |
| scaffold379_46179 | JN900630 | F:ATTTGTCCCAAATGTCATTGGTTT  R:TGCATCATATCTATTACCCCCACC | 57.5 |
| scaffold379_46278 | JN900631 | F:CATTTCCTTGTGCTCATTCTTTGA  R:CATTACCACAGATTCTCCTGAGGG | 57.5 |
| scaffold231_14788 | JN900632 | F:AGACGATTTCACGCCTTGAGAG  R:GTCCATAAGCGCCGACTTTC | 57.5 |
| scaffold101_3759 | JN900633 | F:AACAGGAAGGACCTGGGTC  R:GGGAGTATGTATCCCAGTTGTGG | 57.5 |
| scaffold40_4916 | JN900634 | F:CTGCAGGTGTCTGTGAAGC  R:TCTTGTGATCACGCTGTCCAT | 57.5 |
| scaffold492_47457 | JN900635 | F:GCCTACTACACTGAGGGCTTAG  R:TACATCCACAAAGGCAAGGCA | 57.5 |
| scaffold674_4052 | JN900636 | F:TCTGTCTGAACAACTCAGTCACC  R:CAGCAGTGCTCATCTGTATGC | 57.5 |
| scaffold721_60656 | JN900637 | F:GTCAACTCAAAGCGCTGGTC  R:GAGTGTGCGTTCTACACAGGA | 57.5 |
| scaffold113_13134 | JN900638 | F:CCTGCTCGACTGCAGATG  R:TCCAACTCAACAATGTGCAAGATG | 57.5 |
| scaffold1292_77676 | JN900639 | F:TGTCCATGTCCTCGTTGCA  R:GAGATCGGAAGAAGCAGAGCA | 57.5 |
| scaffold1081_74184 | JN900640 | F:TACACTCAGAGTCCACGGAGAA  R:GTGGAACCACAGGAACAACTG | 57.5 |
| scaffold323_15848 | JN900641 | F:ACGAGTCACAGCACCTCTG  R:CAGATACTTCCGCAGACCGT | 57.5 |
| scaffold300_33833 | JN900642 | F:TGCAGCATCGACAACGTG  R:AAGCAACCGGGACAGATGTA | 57.5 |
| scaffold553_55885 | JN900643 | F:TGGATGCACATACTTCTACATGCAC  R:CACTGACAAACTGGGTGATGACTG | 57.5 |
| scaffold300_33814 | JN900644 | F:CCGGCTACAACCAGAGCTA  R:TCCTGCATGTTGTCCACTTCA | 57.5 |
| scaffold939_5298 | JN900645 | F:CATTCATAGATACACATCCGCTCTG  R:CCTGCATGTTGTCCACTTCATACT | 57.5 |
| scaffold537_55170 | JN900646 | F: TGCAGTCGCTCATTTCACA  R:AGGTGTGACTCCTGGTTAGGTGAG | 57.5 |
| scaffold564_3013 | JN900647 | F:CATCATGGAGTCGCTGTGTTG  R:TGTCCTTCTGCGTGACATCTC | 57.5 |
| scaffold564_3010 | JN900648 | F:TCGCTCTGTCACGTCTTAGC  R:CGAGTCAACTTGCTGTCTCGAT | 57.5 |
| scaffold342_17401 | JN900649 | F:GAAGTGTTCTGTTGTCCAGGC  R:CGGAATGGAATCAGCCACAAG | 57.5 |
| scaffold1172_76121 | JN900650 | F:CTGGTTGTCTTGCTCTGTGG  R:AGAGAACTCCATCAGTTCAGCC | 57.5 |
| scaffold1093_74471 | JN900651 | F:CAAAGAGAGGACGCTGGTG  R:CAAGCTAGCGGTTCTGAAGG | 57.5 |
| scaffold208_10894 | JN900652 | F:AAAGAGGCTGAGGATCCAGG  R:TCGTGCCTGCTTAGAGTTGTC | 57.5 |
| scaffold726_4508 | JN900653 | F:ACGTTTGCATCTGACAATGCG  R:TGAGACCAAATGTTGGTATCCGG | 57.5 |
| scaffold1027_72925 | JN900654 | F:CCTGCAGTTTCTGTTCATGTGC  R:GTGCCTGTTGCCTGACATC | 57.5 |
| scaffold726_4514 | JN900655 | F:GATTGCATGGGGGCTATTGTG  R:TCGGTTCTCTCCCAAAAGGAG | 57.5 |
| scaffold742_61599 | JN900656 | F:GGATGATTCTCGGTTCAGCAG  R:CTAACCTGAACAAAAGAGCCTGTG | 57.5 |
| scaffold342_17412 | JN900657 | F:AATGACCAGGTCTGACCACAG  R:CGTCTTTCACCAGCACGATG | 57.5 |
| scaffold96_4265 | JN900658 | F:TGATTCAGACACTCATGAAACCCC  R:TCAGCACTGGAAACAGGAGTC | 57.5 |
| scaffold733_4818 | JN900659 | F:TTCATCTGGGTCGTCCAGG  R:GTGAACTATCCCTTTAAGCAACTGTCT | 57.5 |
| scaffold416_20724 | JN900660 | F:CACCTTTTACATGGTGGATGCTG  R:GACAATACTCACCTGGGATACCC | 57.5 |
| scaffold416_20725 | JN900661 | F:GATTTCCCTGTTTGTGGGACAAC  R:CCTGACAACCTCTGAAGGCT | 57.5 |
| scaffold532_25748 | JN900662 | F:ACAGATGTAGCCTTCAGCACTG  R:ACTCCAACAGTTGACGTGCAT | 57.5 |
| scaffold146_6342 | JN900663 | F: ACCTTAAAGAGAGCAGTGTGC  R: AGTCCAGTGTGGGGCTG | 57.5 |
| scaffold146_6424 | JN900664 | F:GTTTAGCACTGACACAGGGTCT  R:ACACTGTTGTACTGCAGCTATGAC | 57.5 |
| scaffold508_48667 | JN900665 | F:TGCCTTAACAGTGGCTGGT  R:ATCTTGATGACACACGGGTACG | 57.5 |
| scaffold679_58354 | JN900666 | F:ACAAGCGTGGATGTCACATTG  R:CAGCTTTACCTCCCGTCTCA | 57.5 |
| scaffold495_47739 | JN900667 | F:TCTGTTCCTCAAACTCGTGGAG  R:GGTAACCTTGTTTGACTGTGCAG | 57.5 |
| scaffold726_4509 | JN900668 | F:GCTTCAACAGTATCATAGCACGCA  R:TTGCCTAGGAGACAGAAAGTGGTG | 57.5 |
| scaffold116_13305 | JN900669 | F:ATCAATCAAAACTCTTTGCCCCTT  R:CAACAAGTCAAGCTGGACACAC | 57.5 |
| scaffold146_17513 | JN900670 | F:ACAACTCAGTGTGTGCAGC  R:AGGTCCAGTCAAGTCCGAG | 57.5 |
| scaffold182_7672 | JN900671 | F:GCTGTCAGTGGGCAGTTTC  R:AATTTGGTGCAGAGCCACAC | 57.5 |
| scaffold116_4292 | JN900672 | F:AGGTCATGGAGACATTGGTAATGG  R:CGGTGATCTTGAACTCCCAGT | 57.5 |
| scaffold73_8124 | JN900673 | F:GCCCTTGCAACTCAAGAC  R:ATTCACTGTGGGATCCAGTTTC | 57.5 |
| scaffold1483_78799 | JN900674 | F:CTGTCTGTAGTCTCACCAAGGTC  R:AGCTGGCTAAAGGACATGAGG | 57.5 |
| scaffold1483_78801 | JN900675 | F:GGATCCAGAATGAACCCCTGA  R:GTGACCTGCTGACTGGTC | 57.5 |
| scaffold584_53349 | JN900676 | F:TGAGAAAGAGACGGGGTGAC  R:ACACAGGTTTACCTAAGATCTGCA | 57.5 |
| scaffold195_10343 | JN900677 | F:GCTGGGGATTTAGGTTTCTGTGA  R:TTAAAACCCACCACAACACAGCTA | 57.5 |
| scaffold701_4252 | JN900678 | F:TGTCCAATTTTGTTTTCTGTTGCT  R:AGTCTGAGTCACAGCCTGTCTGAA | 57.5 |
| scaffold701_4251 | JN900679 | F:GTCGACAAAGAAATGAGGACTGCT  R:CATGATTATTCCCAGACACACCAA | 57.5 |
| scaffold431_22007 | JN900680 | F:CTTTAGCTGGGACAAAAAGCCC  R:TCTGACTGGACCCATTGGAC | 57.5 |
| scaffold502_24297 | JN900681 | F:CCACTGAGGTTCACGGTTTG  R:TGTCGGCCAGGGTTATAATGG | 57.5 |
| scaffold292_32854 | JN900682 | F:TAAAGCAGCCTCAGGTAGGAC  R:CCCAGTGTTTGTTAGTGCATCC | 57.5 |
| scaffold639_3780 | JN900683 | F:CTTCTCCAGGAGTTTTGATGCTTG  R:CACTGGCACTGTGTGTCTAAGGAC | 57.5 |
| scaffold292_32816 | JN900684 | F:CAGATGAGTGGTGGCAGTG  R:GGGGTGATTTAAAGACGACAGC | 57.5 |
| scaffold1196_76519 | JN900685 | F:ACAGCAGGAATGTTATCGGCA  R:GCCGTGACAAGGTGACTC | 57.5 |
| scaffold467_52810 | JN900686 | F:GGGAAGCTAATTGATTCACTCGTC  R:GCACACATCTTTTGCTCATGAATC | 57.5 |
| scaffold431_21934 | JN900687 | F:AGAGTAGCCTCAGGGTCCT  R:GATGGAATCCACTTGTGTCTGC | 57.5 |
| scaffold78_9908 | JN900688 | F:GTAAGGCATGTGAACCCGG  R:TGTATGCAGTCGGTGGATGAAG | 57.5 |
| scaffold941_70475 | JN900689 | F:CAGCTCTGCAAACTCACACTG  R:CGACTTAAGTATTGGACGTGCCT | 57.5 |
| scaffold701_4256 | JN900690 | F:CAGAGCTACCATAGGCTGACTG  R:CAGGTGTAATTGGTGTCTGCAAG | 57.5 |
| scaffold595_53946 | JN900691 | F:AGGTTTATCTCTGAGCAGCCTTG  R:AGTATCCATCAGGCTGTCAAGC | 57.5 |
| scaffold139_16935 | JN900692 | F:GATTGAGGCAGCACTGTCC  R:TGTCCATCCTCCTGACACAC | 57.5 |
| scaffold414_21158 | JN900693 | F:CCAAACGGCTCATCAGTCAC  R:CGGTTTCTGTGCAGGGTTC | 57.5 |
| scaffold445_44808 | JN900694 | F:TTGAACTCAAACAGGGCTGC  R:GACAGCATGAATCCAACCTGAG | 57.5 |
| scaffold445_44791 | JN900695 | F:GTTTCTGCAGTGCTTGGACAG  R:CAGTCAAGCAACAAAGCCTGTG | 57.5 |
| scaffold61_6835 | JN900696 | F:TGTCTCCTCAGGGTGGTC  R:ATGGAAGGTGCATGTGTGG | 57.5 |
| scaffold865_67898 | JN900697 | F:GTCCCCATGAGTTGTAGTTGGTTC  R:ATCCATTTTCACCAGAGTAGCTCG | 57.5 |
| scaffold624_58810 | JN900698 | F:ATTGAAAACAACTTGCATCCTTGG  R:TGTTGTGTTTGTGCTTTTAACTGTGA | 57.5 |
| scaffold1419_78622 | JN900699 | F:ATTGTGGTTTGGCATGTCTCC  R:ACTCCTGCTTCCTGGACTC | 57.5 |
| scaffold370_39899 | JN900700 | F:TTTCTGACATAAACAGGCAGAGGC  R:GCGATGCTCCATATTATACCACTGA | 57.5 |
| scaffold193_23892 | JN900701 | F:GACAAACACACAAACATTTACAACCA  R:ATCAATGTCGACTCTGTGATGAGC | 57.5 |
| scaffold439_51074 | JN900702 | F:TCAAGCAATTTTCTGTATGGGACA  R:TGGAAATGTGTGTTGATTGGTC | 57.5 |
| scaffold384_40712 | JN900703 | F:AAACAACGGGCTCTTTCATTAAGC  R:GTGAATGTTTCCCTATGCCAG | 57.5 |
| scaffold566_52269 | JN900704 | F:CACACATCTCCATGTGCTGG  R:TTTCCTCTCCTCTGACCTGC | 57.5 |
| scaffold472_46619 | JN900705 | F:CTCTACTTCATCTCCCCAACTGGA  R:TCATCAGGCTTTACTGATGGG | 57.5 |
| scaffold472_46620 | JN900706 | F:GAGGCTGTTCCAGAGAGTGAAAGA  R:ACAGCCCACTCAACCTAGAAACAG | 57.5 |
| scaffold370_39921 | JN900707 | F:AGACATTCCTCAAAGCAGC  R:ACCTGCCCTGTTTGCAGATAG | 57.5 |
| scaffold414_48885 | JN900708 | F:GGTCTGTTCTGTGTAAATGGAGGC  R:AGAATATTTGCATCTGCACACACC | 57.5 |
| scaffold709_4322 | JN900709 | F:GCATTAGAGATTTGCAGACCTGC  R:TCTACGTTGGTGAGGACAGC | 57.5 |
| scaffold166_9305 | JN900710 | F:CTGTGGTGAATCAGAGACAGGAG  R:ACATCACAAGCAGCGACTG | 57.5 |
| scaffold170_20574 | JN900711 | F:ATAGGTGGAGCACCGAGAAG  R:TCACCAAAAACCCTCAGTCGTAG | 57.5 |
| scaffold131_231 | JN900712 | F:CAGAATCAGTCTCAGAACACCTCG  R:GGTAAAGTGGACCCTCACCAA | 57.5 |
| scaffold1016_72663 | JN900713 | F:CATCTACTCCATGACCGCGA  R:CACCTAGATCTTACACACTGGACC | 57.5 |
| scaffold603_54669 | JN900714 | F:GTGTTGAGAAGGATTCACAGGC  R:GGTCAGATGTAGTCCCAGGAG | 57.5 |
| scaffold1016_72669 | JN900715 | F:AAACATTAGTCAGCCAGCGC  R:AACAACAAACCTAGCATTCGGTG | 57.5 |
| scaffold225_788 | JN900716 | F:AACCGTAACCTTGACGATGGAG  R:GACGGACAACACAAGAAGTTTCC | 57.5 |
| scaffold55_1327 | JN900717 | F:CTTAGAGCCCCGTCTCTTTACACA  R:ATGTGGAACACAGACTCTCAACCA | 57.5 |
| scaffold762_62836 | JN900718 | F:TGAACATTGCTGAATTCACAGCTTG  R:GCAGACACAGCATGAGTGTG | 57.5 |
| scaffold1944_79163 | JN900719 | F:GCCAGTGGGGATGAATGTATG  R:GCTGCAGGTGTTAGACCAG | 57.5 |
| scaffold139_16914 | JN900720 | F:GTAAGTGTGCATGTGCCAATGT  R:GCTCTGTCAGGCCTCTGA | 57.5 |
| scaffold949_70805 | JN900721 | F:GCCCAACTGCTTCTGTTGAAT  R:AGGACAATAGAAGTCTGGGCC | 57.5 |
| scaffold5_2054 | JN900722 | F:CAAGTGTTCTGCCACACTCTTTGT  R:ACAGACGTGTTGAAATTCCTCCTG | 57.5 |
| scaffold307_35970 | JN900723 | F:AACAAAACTCCCCTTCCCTCTC  R:CTGCTCTACTACAGTTCAGTTCAATGAG | 57.5 |
| scaffold819_65626 | JN900724 | F:AGAAAAGAGGAGTGGAGCTGG  R:GTTGAACCACTCAACCATGAGC | 57.5 |
| scaffold165_19737 | JN900725 | F:CGAACCCCATTCACACAGC  R:TCTCTCAGCAGATGCTTCACC | 57.5 |
| scaffold60_10247 | JN900726 | F:ACCTTTTCTTTCTCTCTTTCTGGC  R:TCCCAAGATTCTCTTTCTGTATTGA | 57.5 |
| scaffold916_5192 | JN900727 | F:GTGGACAGCTATAGAGTGACAGCG  R:GAGTAGTTGTCAGGCACGTCACAG | 57.5 |
| scaffold534_2939 | JN900728 | F:GTTTCAAGGCTCTTTTCAGAC  R:AGTGGCTACAGTGCAGAGTGTGA | 57.5 |
| scaffold165_19721 | JN900729 | F:ACACAGTTCCAGTCTCTGACAC  R:GCCTGACTGCTCTACCATCT | 57.5 |
| scaffold881_68459 | JN900730 | F:AGACCTCCCAAAACAGTCGAG  R:AAGGAGGTCATCGAACTTCAGC | 57.5 |
| scaffold60_10489 | JN900731 | F:CACTCTCTCGCTTCTTCTTACGTC  R:AACATGTCAGAAAATGAGG | 57.5 |
| scaffold16_1588 | JN900732 | F:CCTACATCGTTGGTGGCC  R:TGCACCAAATTGCACAAACTCA | 57.5 |
| scaffold1815_79140 | JN900733 | F:TTTGGACGCAGCGTTGATC  R:CGAGCTAACAAGGGAGCG | 57.5 |
| scaffold1079_74118 | JN900734 | F:GAACGGGTGTAGGATGTAACCTC  R:AGTTACACGCACGTGTACGAG | 57.5 |
| scaffold126_5194 | JN900735 | F:CAGGTAGGCCAAGTCTGTAGG  R:TCTGAAGTCGAGAAGGTGCAG | 57.5 |
| scaffold874_68210 | JN900736 | F:GCTCAGTGATGGGGAGCTA  R:ACCTGTCAGACCCTCTAGCA | 57.5 |
| scaffold199_23892 | JN900737 | F:AGGAGATGTAACAGTGGTGTGG  R:ATGGGAACATTCGCAGGAAC | 57.5 |
| scaffold1240_77039 | JN900738 | F:GGAGGTGAACATTGTGTTCTGC  R:TTGAGATCTGGGCCTGGAC | 57.5 |
| scaffold126_15449 | JN900739 | F: TCATTCCGAGTGAGTGGG  R:GAACGTCCACTTGGTATTTTCCTG | 57.5 |
| scaffold513_2856 | JN900740 | F:GGACTGTTCAGGCTCAGATGT  R:GGACACATTCTTCATCGCAGACT | 57.5 |
| scaffold126_5199 | JN900741 | F:GCAAAAGAGCGTGGACTGAAG  R:GGAGTGTGATCAAGGTGGATTCA | 57.5 |
| scaffold66_7572 | JN900742 | F:GATAGAACGATGCAAGTACAGGAGC  R:GAGAGCGTGTTTTATGCTCACAGA | 57.5 |
| scaffold1419_78615 | JN900743 | F:GAGGGACAAGCAGTTGTGTTC  R:GCGAGAGGGTCGTTGACATA | 57.5 |
| scaffold513_2859 | JN900744 | F:AGTGAGCGAGTGAGATGCTTC  R:TCCTGAAAGTCCTGATGTGGTC | 57.5 |
| scaffold423_48491 | JN900745 | F:TGGATTTTAAGTGTGGTCTCACTGC  R:TGTCACTTCACACACTGGGTC | 57.5 |
| scaffold297_1030 | JN900746 | F:CTGAAATTGGCCTTGATGTGGAC  R:CGTCTTTCATCACCGAAATGTGG | 57.5 |
| scaffold67_112 | JN900747 | F:CCTGTTATGACTTCCATGTAATGTGTG  R:CCCAATCCCTATCTGAGGTC | 57.5 |
| scaffold541_50683 | JN900748 | F:TCGTAGTTTAGGCCCCAGATG  R:CTGCAGAAGTGTTTCCATAGCAAC | 57.5 |
| scaffold516_2730 | JN900749 | F:GCTGCCGTTAGGATTTACATGG  R:AATGGCTCTTCTCTGCTCTGAC | 57.5 |
| scaffold516_2732 | JN900750 | F:CAGCTTAAGCAATCAAGAGCACAA  R:GCAGCAACAGACTCAACGACC | 57.5 |
| scaffold239_12877 | JN900751 | F:AGAGAGTGTGAGGTGGTGTG  R:CTGTGAGGGCAACAGAGC | 57.5 |
| scaffold239_12872 | JN900752 | F:GCTGTTATATGGCAGCAGCATG  R:TGCAAAGACAATCAGCCAAGC | 57.5 |
| scaffold415_48711 | JN900753 | F:TGGAAGAAGTTTATCATGCAAATCC  R:CAGGCCTGTGTCCTTACAAAG | 57.5 |
| scaffold106_187 | JN900754 | F:CAGCACTGAGTTAAAACGCATC  R:TGATTTCAGCCTCAAAATTGTC | 57.5 |
| scaffold67_96 | JN900755 | F:CACGTGTTCTGTGCTGTCTG  R:ACTTTGCTGCAACCTACTCTTTGC | 57.5 |
| scaffold928_70156 | JN900756 | F:CCCTCATTGCCACAGTCAG  R:GACTTCTCGTCGGCAAACAC | 57.5 |
| scaffold376_1606 | JN900757 | F:TCGTTCTGTTCAGTCTCAGGAATG  R:AATCACGGTTTATTGTGCCGATAC | 57.5 |
| scaffold554_51494 | JN900758 | F:ATCACTCACATGGTGACGTGATG  R:ATAATGCAACAGGCCACAGC | 57.5 |
| scaffold735_61277 | JN900759 | F:CTTACCAGCCTTGTCCAGAGT  R:GTTGATGGAATGCGTGTAGCT | 57.5 |
| scaffold265_30015 | JN900760 | F:GGAGACAAAAGTGATGTTGCAGG  R:CCCAAACCTGACAATAGACTCCA | 57.5 |
| scaffold745_4673 | JN900761 | F:CGGTACCGTCACTCCTCTGACT  R:CACTTGGAGCAGATAAGACGAACA | 57.5 |
| scaffold1130_5358 | JN900762 | F:GCTGTCCACAATAAATGGAAGTCA  R:ATTTGGACTACTGGTCCTGAAACG | 57.5 |
| scaffold329_16132 | JN900763 | F:TCAGGTGTTCGACATCAAGTGG  R:CAAGACGAGGGCAAAGCTG | 57.5 |
| scaffold393_19395 | JN900764 | F:CTGTTGAAGAGATCATGTGGCAAC  R:GACGTTTGGTGCTCACACATC | 57.5 |
| scaffold346_37702 | JN900765 | F:GGCCTATGTGAATTCCAGGC  R:TGCCAAAAGTCAGTCGAGCAT | 57.5 |
| scaffold570_52520 | JN900766 | F:CATACTCAGGCAAACCTGCAC  R:CCTTCAATCTGGTCCTTGGC | 57.5 |
| scaffold50_53 | JN900767 | F:GGGACAGACAATATTTCACCGC  R:TGTGGACAAGGTTCCACACA | 57.5 |
| scaffold1130_5359 | JN900768 | F:TTTGAGGGCAGAAGTTAGGAGATG  R:TTTAACCTGGAGGGTCAATC | 57.5 |
| scaffold50_89 | JN900769 | F:CGGTCACTTTCCTCATAAACCAGT  R:GCTTCATTCATTACAACTCCTCACAG | 57.5 |
| scaffold84_2215 | JN900770 | F:TCCACCATGACCAGACAAAACA  R:GATGTTGACATGCTTAAGGGCAC | 57.5 |
| scaffold1149_75544 | JN900771 | F:CAGCCTGTGATAGCAAGAAGC  R:GAGAGTACTGCCATACCAGAAGG | 57.5 |
| scaffold1026_72908 | JN900772 | F:TCTGAGAAGTCACATGACACCC  R:AGACATACGCAGTGACGGATATG | 57.5 |
| scaffold109_12233 | JN900773 | F:GTGGGATGGGTGGTATTCTGTACT  R:TATTGATTAGTCTGAATGAGGCGG | 57.5 |
| scaffold786_63956 | JN900774 | F:GCAGATGAATTACCCAGACATGC  R:AGAGTGGAAACAAGAGTCACCAC | 57.5 |
| scaffold339_43856 | JN900775 | F:CATAAAGCCATCAATGAATGTGAA  R:CTAATATTTGTGGATTTGTGGATGC | 57.5 |
| scaffold1026_72909 | JN900776 | F:GTGACTCACAGGATTCAGGGA  R:AGGAACAGATTGCTGTTCTAGGT | 57.5 |
| scaffold30_144 | JN900777 | F:CCATAATGTGGGGGCTCG  R:CCAACGCAGACACGTTAACAC | 57.5 |
| scaffold780_5062 | JN900778 | F:TGTTCTTTGTGAGATGACCTTGACC  R:AGGTGAGGGGTTTAACAGAGTGTG | 57.5 |
| scaffold84_2213 | JN900779 | F:GCGTGGAAAGTTTGTTCGTTGAT  R:AACTGCACCAAGCACATCAC | 57.5 |
| scaffold570_52503 | JN900780 | F:AGAAGCACCGAAGGTTATGGTC  R:CTACAGCACCGAGGGTGAA | 57.5 |
| scaffold780_5077 | JN900781 | F:TGCTTACAGTGATCATTTTGGGTC  R:TCCCATTTGGCATCAATAAAGT | 57.5 |
| scaffold94_11057 | JN900782 | F:ATACCTGCAGGGTAGACAAAGCAC  R:AGCTTCCATCCCAGTCAGGTAAG | 57.5 |
| scaffold204_24115 | JN900783 | F:GTCACTTTGCACCAGAGCAG  R:GAAGATGGTGAGCGAGAGGTT | 57.5 |
| scaffold340_37241 | JN900784 | F:AACAGGTCTAGCTGCAAGCA  R:GATGAAGACATGGACGGACACA | 57.5 |
| scaffold142_5865 | JN900785 | F:CATAAACACATGGCAGGAGAGC  R:TCAGTTCTCTCAAAATGAATG | 57.5 |
| scaffold163_19588 | JN900786 | F:TGCTTTCACTTCTGAGGCTGA  R:CAAGCAATCTGCAGTGTGTCA | 57.5 |
| scaffold453_22637 | JN900787 | F:GACAGGTCGTGTGCAATGTG  R:AATGAATCCAATGACCCCAGAGTC | 57.5 |
| scaffold906_69496 | JN900788 | F:GCAGCAGAACAGACTGTCAC  R:TCTGCACGTCAGACTGGATG | 57.5 |
| scaffold381_40416 | JN900789 | F:GCAGACACTAGTCGAAGCGA  R:GAGATGGAAGAGGAGGACAGG | 57.5 |
| scaffold465_46245 | JN900790 | F:CTGCAGGATGTTTATGTGTGTTAGTG  R:TTGAGATAGTGGGTCTGTGGTG | 57.5 |
| scaffold210_24624 | JN900791 | F:CTGACATCTGCTCGATTACCTCCT  R:GTTTCTCCTGCACCGACTAC | 57.5 |
| scaffold210_24612 | JN900792 | F:TGCAGTGTCTTAACTGGTGTTGAC  R:ACAGTGACACATGGAAACAAGTGA | 57.5 |
| scaffold781_63716 | JN900793 | F:GCTAGACATTTGACTCTTTTTGGG  R:TGTGCAGTGCTGTAATATTCTGGTG | 57.5 |
| scaffold343_37432 | JN900794 | F:CCTTTGCACATGTTGTTTTCTCAC  R:TGGGTCACACAACCAAATACAGTC | 57.5 |
| scaffold697_59597 | JN900795 | F:AGACACTAACACAGGAGCTGAC  R:AACCATTTAGCCAGGCTCTGTA | 57.5 |
| scaffold196_23349 | JN900796 | F:TTGCCTGGATGAAGTGCATG  R:CTGACACTGCCTCTGCTG | 57.5 |
| scaffold478_46912 | JN900797 | F:ACATGGATTACCTCTGGTCTCAG  R:CCTCAAAGCGAAGTGAATCTCC | 57.5 |
| scaffold1156_75699 | JN900798 | F:ACATAGTCACGCAAGCTGTCA  R:GATCAGAACCAACCAGCGG | 57.5 |
| scaffold1362_78380 | JN900799 | F:CCACAGCCTGTTGTATGTTG  R:TCATCTATCACGGATGACAGG | 57.5 |
| scaffold1084_74297 | JN900800 | F:TGGCAAAACACGTCAACTGATTG  R:GAAGTGCAAACCTGTGCGA | 57.5 |
| scaffold1305_77853 | JN900801 | F:TCTGTTCTCACCTGTCATCTGG  R:TCTGAAGGTGAAGCTGTGTTTGAC | 57.5 |
| scaffold229_26658 | JN900802 | F:CAACTGAATGATTTATCGCAACGA  R:ACTTGCCCTGTCACAATC | 57.5 |
| scaffold528_50054 | JN900803 | F:TGTGCAGAGTCAGAGAGCTG  R:CTGATGCAGGCAGGAATGAC | 57.5 |
| scaffold1430_78672 | JN900804 | F:GAGCAGCAGTATGTCCGC  R:AACAACGTCCCGGGAACT | 57.5 |
| scaffold1430_78674 | JN900805 | F:AGTCACATTGAAGCACACATGC  R:GAAACACACGCTGTCCTTGG | 57.5 |
| scaffold487_2545 | JN900806 | F:GACAAAAGTTCTGGCCACGAG  R:GCACACTTCTGGGAAACAGG | 57.5 |
| scaffold1018_72725 | JN900807 | F:CACTTCACAGGCAGACCAC  R:TGTGAACTTGACCGATACGGTG | 57.5 |
| scaffold259_13646 | JN900808 | F:GCTGCTCAGACACAATGTCC  R:CGCTACTTTCTGTGTGCAGTG | 57.5 |
| scaffold498_48138 | JN900809 | F:GAATGGAAGCAGGTGAGCTG  R:GCTGAGCACTGAAGATGCAA | 57.5 |
| scaffold581_52969 | JN900810 | F:GGTTTTGCAGTTTAGCTCAGTGG  R:CCTCAAATTGTTATTGAGCTGGGG | 57.5 |
| scaffold551_55114 | JN900811 | F:CAGACGGCTTCTCAAAAGACAAGT  R:TCCTGTAAACCATCCTCATTCACA | 57.5 |
| scaffold225_717 | JN900812 | F:CGTATCACAGTTTGCTGCCTTAC  R:GCTATCATCTGCTGCCACTG | 57.5 |
| scaffold420_1898 | JN900813 | F:GTGGACTGTTGGTCCTTGG  R:AGTTTCGACAGGGGTACAGG | 57.5 |
| scaffold748_4626 | JN900814 | F:GATGAATCAACGTGTGTTGACAGG  R:CATGGGGACACTTGTTGTTGG | 57.5 |
| scaffold748_4632 | JN900815 | F:GCCAGTAAATCACCAATACGCC  R:TGAACAGAGAGTTCACAGCACTG | 57.5 |
| scaffold748_4629 | JN900816 | F:TCCTGCTCTGCACTTACACAG  R:GGTAAACATGTGACTGGGCG | 57.5 |
| scaffold963_5291 | JN900817 | F:TGTCACTCACACACGTGCA  R:GACGGATGCTCAAGTGGAGT | 57.5 |
| scaffold59_1608 | JN900818 | F:AGGAACAGTCTACCCTGGAGA  R:GTTACGCTGCTGTCGAACAG | 57.5 |
| scaffold963_5289 | JN900819 | F:TCTTCTTTTACCTTTTGGCTGTGC  R:TGCCAGTCTATCAAACAGAACATACA | 57.5 |
| scaffold253_29195 | JN900820 | F:TCACTGACAACACGCACAAAC  R:GGGTGAGTTCGAATATCAGATCG | 57.5 |
| scaffold547_50927 | JN900821 | F:CACAGGAGCAGATCAACAGC  R:GCCCAAGTATGATGACAGCC | 57.5 |
| scaffold547_50928 | JN900822 | F:ACAGCAATGTTTGCCTCAGTG  R:GTGGGAGTTTGTACAGCAGC | 57.5 |
| scaffold230_26672 | JN900823 | F:CAGGTGCATTGATCATAGGTGC  R:CTGTCCTCAGAGGGATCCTG | 57.5 |
| scaffold448_2133 | JN900824 | F:CGTCAGCTCCCTGCTGATTTACT  R:GGTTCACACGGTTAACACAACTCA | 57.5 |
| scaffold605_3367 | JN900825 | F:GGGATGGCTTGATATGCAGTTG  R:GACCTGATTAAAAACAGTGGGCG | 57.5 |
| scaffold69_2052 | JN900826 | F:TATTCAAGTGCAGTGAGCCACA  R:CTTCAACTTCTTTGTGAGCAGCAG | 57.5 |
| scaffold605_3374 | JN900827 | F:CTGTGTGCATGTGTGCATGTG  R:CTTGTTCTTCCCCAGGTTGATG | 57.5 |
| scaffold44_1155 | JN900828 | F:GGAGCCTGTGGAAACTGTACTGAG  R:TCACCGTTTTCATTTGTTGTTG | 57.5 |
| scaffold605_3375 | JN900829 | F:TAAACACACATTCAGCACAAAGCC  R:AGTGAAGTGAGCATGGAGG | 57.5 |
| scaffold253_29170 | JN900830 | F:ACCTGGCAAAACCGACTTG  R:CCACACAAATGCACACACGTAC | 57.5 |
| scaffold588_3270 | JN900831 | F:ACACACTGTCTCGAGTGGTC  R:TCAGGTCAGTACTGGTGCAG | 57.5 |
| scaffold588_3269 | JN900832 | F:GTGCAGTTAGAATGACAGCAGC  R:CATCCCGAGTGGTTTAGGTATCC | 57.5 |
| scaffold588_3261 | JN900833 | F:CAGAACCCGTCAAAGAAGCC  R:GGCCACAGGAAACTTTACAGG | 57.5 |
| scaffold437_21627 | JN900834 | F:GTTGAGAGCGATGTGCGTC  R:TCAAGGAGCAGATGAAGGTCAG | 57.5 |
| scaffold68_122 | JN900835 | F:CAGCTCTTAAGACTCCAAACGC  R:CTGATTGAGTGACGGGCAC | 57.5 |
| scaffold648_3980 | JN900836 | F:TGATGTCACTGGACAGTCGTC  R:CTGTCCTTAAACACCGACGC | 57.5 |
| scaffold648_3981 | JN900837 | F:TCCACAAAGCTCTGGGAGAG  R:CAGCACAAAGTAAAAAGGTCTCGG | 57.5 |
| scaffold503_2817 | JN900838 | F:GGCAGGAAGAAGTGAAGTGC  R:AGAATAGGACACATGGTGTCTGC | 57.5 |
| scaffold90_10871 | JN900839 | F:CCTCACGTCAAAGTGTCCTCATAG  R:CATGTTTGCCAGTTAAGCTCTGG | 57.5 |
| scaffold1123_75036 | JN900840 | F:TACAGCTCCTCCTCTGCG  R:TTCCGCTAAAGAACGGCTACA | 57.5 |
| scaffold89_8565 | JN900841 | F:ATCAGTCTGTGTAAATGCTTCGGA  R:GTACGTGGCTGCTTTTCTTC | 57.5 |
| scaffold437_49694 | JN900842 | F:AGCCTTTAGATCCTGAGGGTCAGT  R:ACTTGCAGGAACCCTGACAGTCT | 57.5 |
| scaffold437_21654 | JN900843 | F:TTGTCATGGTAACAGGCAGTGT  R:TCCATGTGAGTCGGAGCATC | 57.5 |
| scaffold473_46635 | JN900844 | F:AATATTGGCTGCGCTTAGCAAC  R:CCAAATAACAGTGTGCTGGACG | 57.5 |
| scaffold765_62882 | JN900845 | F:CTGTAATGGGATAGACACGAGGT  R:CAGTGTAACATGGTGCCGATAC | 57.5 |
| scaffold891_5138 | JN900846 | F:GCCTCAGATCTTCTGTCTATGCGT  R:ATTCCTCTGCTTTCCCTCTCTGTT | 57.5 |
| scaffold279_31523 | JN900847 | F:ATAAGTTGACCACCTCAGGACCAC  R:GAATGACACTTGATTCAGGGGATT | 57.5 |
| scaffold929_70198 | JN900848 | F:CATTCAGCCAAACTTTGACAACAA  R:TCCCAGTTGTCTGTTTGCATTAAG | 57.5 |
| scaffold739_61526 | JN900849 | F:GCTAACATGACCTGGGTGG  R:GGCAAATACTGACACATGGACC | 57.5 |
| scaffold1441_78705 | JN900850 | F:AACTAATTTGCTTCAGACAAGCCG  R:GGCATTTAGTTCTGAAGGTTCCTG | 57.5 |
| scaffold1000_72170 | JN900851 | F:TCATCATTACCAAGACAAAGTTTCCTC  R:AGCTCTCTGTACAGCTCAGCG | 57.5 |
| scaffold929_70182 | JN900852 | F:GCGTTTACTGGCACAAACATCTAC  R:GGATGAGTAACTGAGGGAAACTGG | 57.5 |
| scaffold1024_72855 | JN900853 | F:GCAATTTCCTCAGAGAGCTGGTT  R:TGAGGGATAGTGAAGAAGAGCCTG | 57.5 |
| scaffold586_53473 | JN900854 | F:CAGATCTCACAGAAGCCGACA  R:AGACACACACATAGGGCGATG | 57.5 |
| scaffold120_14792 | JN900855 | F:GACAGCTCAGAGATCGCATGT  R:CCTGCTCTTTTTGGAAGTCACC | 57.5 |
| scaffold108_14497 | JN900856 | F:TTTCTCCTGATCACCTTCACTTCC  R:ACTGCTGCGTGACTCCTGAAC | 57.5 |
| scaffold422_43752 | JN900857 | F:TGAGTCTGGTGAGTTAATGCC  R:AGCAGGTACCACAATTTCAATTCC | 57.5 |
| scaffold209_23945 | JN900858 | F:ATGTCCTCTGAAGTCATGGAGACC  R:ATAGGGGCTTCATTCAACACACAT | 57.5 |
| scaffold1274_77523 | JN900859 | F:CGTTGTGACCTCAAATGCACA  R:CAGACCCCATGAGTCCAGT | 57.5 |
| scaffold655_3859 | JN900860 | F:GTGCTCGTTGGTCTGAATAAACCT  R:TAACTTCCCGACATTCATCG | 57.5 |
| scaffold1157_75734 | JN900861 | F:TCGTGACGTCTGCACATACATAGA  R:ACGCAGTGACTCTGGTCAAAGAG | 57.5 |
| scaffold1157_75730 | JN900862 | F:TCAGGATGCTGCGTTTACAGTATG  R:CACTACATTACGCTGCCTGTTTTG | 57.5 |
| scaffold351_38207 | JN900863 | F:TCCTCAGGGTGCTGGTTATG  R:TGATTTTGAATGTGCAGCTCTGC | 57.5 |
| scaffold1272_77503 | JN900864 | F:CTTGATGCTCTTCCCTGAACG  R:ACAGCCAGATTTGTGCTGTAGA | 57.5 |
| scaffold258_29492 | JN900865 | F:AGGAAGCCTCTTCATCATCGTC  R:CCTAATCAGCTTCCTGCAGTGA | 57.5 |
| scaffold1201_76581 | JN900866 | F:AAGAAGAAAACACCCACCGC  R:CGAACCATCACCAGATGTTCC | 57.5 |
| scaffold863_67755 | JN900867 | F:CGACGTCTGTTCCTGGATGAT  R:TGACGGAGATGGATCCTGAC | 57.5 |
| scaffold655_3851 | JN900868 | F:CACCTAACACCAGGACCAACTGAC  R:GTTCGATCCTCAGCCCAC | 57.5 |
| scaffold844_66688 | JN900869 | F:GGAAAGGAACTGATCGCTGC  R:CTGCAGAACATGAGTGTCAACG | 57.5 |
| scaffold218_25590 | JN900870 | F:TGTGTCTCTCACTGAGGCC  R:TGAAACTGAACCATGGTCGGT | 57.5 |
| scaffold660_57300 | JN900871 | F:TCCTGTAACCTGCTACGAGAGGAC  R:CCCCTACGGCAATAGTGATTATGA | 57.5 |
| scaffold91_2336 | JN900872 | F:ATTCACAAGGCTGCTCATGGTA  R:TGAATATCTCAGGACCCAGACGT | 57.5 |
| scaffold455_24027 | JN900873 | F:GAAAGCACAGCTCTCATGCTG  R:CGTCTCTCAGACGAGGATCG | 57.5 |
| scaffold455_24040 | JN900874 | F:ACCGAAGCTAGGTTAGCTCAATG  R:CTCTAATGAAAACGTCCCGGTCT | 57.5 |
| scaffold582_3176 | JN900875 | F:TCACAATAGTAGTAAGTACGTGATGCCC  R:TTTCAATCTTTTTCATTTAGCGGC | 57.5 |
| scaffold468_2408 | JN900876 | F:TGAACATCAGAGTGATGAGCTGTG  R:GTAAACGCTTGTATTTGCTGACCTC | 57.5 |
| scaffold582_3195 | JN900877 | F:CCCCGCTAGTTAAAGCAGAG  R:AACATTGTTTGAGGACCCTGGAT | 57.5 |
| scaffold656_57162 | JN900878 | F:CGTTCTTTGCCATTTCAGAGTGG  R:ACAGTGAGATCCGACACTTGG | 57.5 |
| scaffold818_65579 | JN900879 | F:ATTGACTTCGTGAGACACAGACCA  R:CTAGCTCCTCCACGTACCTGTG | 57.5 |
| scaffold468_2409 | JN900880 | F:TCTGTCTCATTCTCACGCAAGG  R:CTGTAAAGGATCCAGGCATGTG | 57.5 |
| scaffold818_65550 | JN900881 | F: ACAAGGAGATGCACAGC  R:CTGACACAATTCTGGCCATTAAGA | 57.5 |
| scaffold130_16114 | JN900882 | F:CACTTCGTATCTTGGGGACCA  R:GAGGATTTGTGTCTTGCCAGG | 57.5 |
| scaffold91_2339 | JN900883 | F:CTGATAATGACGCCAGCGTG  R:GACCCAACATCAAACAGCACATC | 57.5 |
| scaffold136_5338 | JN900884 | F:ACTGACTGAGTGACCGACAAG  R:TACTCTTCCTGGGTGGTGC | 57.5 |
| scaffold2_194 | JN900885 | F:GAATGTCAGTGGGCACGAAG  R:GTGCACTTGCAGCATATACGATC | 57.5 |
| scaffold2_220 | JN900886 | F:CACTGCACAAACCCACCTT  R:GAGGTAATCCATCAGAGGCGT | 57.5 |
| scaffold947_70672 | JN900887 | F:GAGGATCTGTCCTTCACCACCA  R:GAAAATGTTGGGGGAAGTAAATCTG | 57.5 |
| scaffold137_16826 | JN900888 | F:CTAGTTCCTTCATCCCACCTCCTT  R:CAGTCTGAGGGAGAGCAAAAGAGT | 57.5 |
| scaffold85_10316 | JN900889 | F:TGTATGCAGAGGGTTGAGAAATGA  R:CTGGTGAGTGTCCTGGTCTGTG | 57.5 |
| scaffold1564_79008 | JN900890 | F:TCGGCACTAAGCTCTCACACTGTA  R:AGACCAGTGAACCACTGAATGCTC | 57.5 |
| scaffold482_47066 | JN900891 | F:ATGTCAGCCAGGTATGAGAACACA  R:TGTTTCATAAGCCTGTTTGTTGGA | 57.5 |
| scaffold175_20856 | JN900892 | F:TCCTCTGAATCCCCCATTCATG  R:ACACATATGGACAACCTGTGACC | 57.5 |
| scaffold1107_74719 | JN900893 | F:AAGAGAGGGAGGCCTTGATTAC  R:TCCCAGACCAATGGCTCA | 57.5 |
| scaffold32_6886 | JN900894 | F:ACAAAGTCCCTGTTTCCTAAGGCT  R:CAAATGCAAAGCACAGAGAAGATT | 57.5 |
| scaffold32_1818 | JN900895 | F:TGACTGAATGGAGCTCTGATGG  R:GCTTCCTCACATGACTGGATCT | 57.5 |
| scaffold161_19142 | JN900896 | F:GGATGGTACAGATGGTATGAGACAG  R:ACTACGCCTCACACAGAAGG | 57.5 |
| scaffold355_17679 | JN900897 | F:AACAAGGTAAATCTGGACAGGAGG  R:GGGACTTGATTTTGCCTAAGCC | 57.5 |
| scaffold264_29910 | JN900898 | F:CATTTAGGCCTTGCCCTGTAAC  R:CTGCATCAGCCACGCTATTG | 57.5 |
| scaffold4_388 | JN900899 | F:AGCTACCTTGTTTAACAGCATCTACAG  R:CACTGTTAACACCAGATTTGTCAGC | 57.5 |
| scaffold567_52304 | JN900900 | F:GTGTGCCTGGGTATGTTGTG  R:GTTCGTTGACAAACCAGGGG | 57.5 |
| scaffold567_52284 | JN900901 | F:TGCCTCTCTAACACACTGACG  R:ACCTACCGTTAAGCCACAGG | 57.5 |
| scaffold32_1985 | JN900902 | F:AGCCAAAGCACCTGAATTGCAT  R:CACATAATGCTGTGGGGAATGC | 57.5 |
| scaffold404_21594 | JN900903 | F:GAAAAAGAGTTGAAGGTCGGCG  R:AGCTTGTGCAGGAGATGACTC | 57.5 |
| scaffold664_57397 | JN900904 | F:GTGTAATGTCACATGTGGTAGGC  R:CAAATAGCAACCATGAACCACTCG | 57.5 |
| scaffold953_70904 | JN900905 | F:AGGTCGCACTAATGTCACCAC  R:AGATTTATCCATCACTCCTGGTGC | 57.5 |
| scaffold270_13816 | JN900906 | F:CAAACAATGTCACGGTCCTCC  R:ACAGCTTTTTGTTGTTGTGCGT | 57.5 |
| scaffold427_48573 | JN900907 | F:CGTGACATTCAGATAATGGCAG  R:CATACTGAAAAATGTGAGGTGTGTG | 57.5 |
| scaffold118_14698 | JN900908 | F:CCAGAGAGACCGAGAATCCC  R:CAAAGCCTGGTGCTAATGAGG | 57.5 |
| scaffold1301_77787 | JN900909 | F:AGGGTTATAGGTGTGCTCCAAG  R:AACAATGGCTCACAGGATTTCG | 57.5 |
| scaffold664_57387 | JN900910 | F:TGTCTCTCATTGTCTGGCACTG  R:CTAAACGGAGGCAGCACTG | 57.5 |
| scaffold855_27686 | JN900911 | F:ATCCAGGTTTCAGTGGCAGT  R:AGACTTTCCCAGGGCG | 57.5 |
| scaffold528_54065 | JN900912 | F:TGCACACCTCTTATCTAGCCACTG  R: CTTGATGCTCCTATTGCTC | 57.5 |
| scaffold404_21589 | JN900913 | F:GAGTTCCAGTATCTAGGCCAGC  R:CAATGTTCCTAAGCAGGGATGGA | 57.5 |
| scaffold362_18422 | JN900914 | F:ACAGTTGCTATGACAGGATGTGAC  R:CACTCATGGACACCAGGGAT | 57.5 |
| scaffold111_3890 | JN900915 | F:TCACTTGGCTCTCAGTGTCC  R:GCCTTTTCACATACTGTCCTTGG | 57.5 |
| scaffold270_13812 | JN900916 | F:GTGGGAGTCATTAATGTGTGGC  R:TGGGGTGAATTGCAGGGTATT | 57.5 |
| scaffold609_3379 | JN900917 | F:TGTGCAGAACAATGTCCCTGT  R:TGCATCAGTGCTTCTGGAGAG | 57.5 |
| scaffold26_23 | JN900918 | F:TATGAGGAAGTGAGTGGGCC  R:GTTCCTGGCTAAAGCTAAACAGC | 57.5 |
| scaffold653_3887 | JN900919 | F:GTGGTAAACCATGCGTAGCG  R:GACACTTTTATCCAAAGCGACTTCC | 57.5 |
| scaffold810_65165 | JN900920 | F:TCTTTCCGACTTGTACAGACATGG  R:CTTACAGTGCCGAAGTTCCAG | 57.5 |
| scaffold1037_73179 | JN900921 | F:GTCATGACAAGGAGGGACAGT  R:TGTGTTGTCCCCAAACTGC | 57.5 |
| scaffold589_3352 | JN900922 | F:ACACCTTCACTTCTGGGACAC  R:CCTCATCCAGGAGCACATTGA | 57.5 |
| scaffold790_4911 | JN900923 | F:GTGCAATGTCAAGGGTCACG  R:TTCCTGAGTACAGATTGGCTGG | 57.5 |
| scaffold589_3353 | JN900924 | F:GGATAAGGTTCTGGTCAGGCA  R:TTTGTCCTTGTCGCTCAGGT | 57.5 |
| scaffold35_4096 | JN900925 | F:CCAGGTTATTATCGGACAAACC  R:TGAAACAGAATTCAAGGGGAAAG | 57.5 |
| scaffold589_3346 | JN900926 | F:ATTGGACGTGCGATAGAAAATG  R:ACAGCTGTTTGAGAAATGCC | 57.5 |
| scaffold497_2616 | JN900927 | F:TTGATCCATGGCCAGGT  R:GGCGCTGTGGTTGTGTGTTA | 57.5 |
| scaffold790_4920 | JN900928 | F:CTCGCTACATGTCACTCCCATAC  R:GAGAGGCCAAAGACGTGTCT | 57.5 |
| scaffold497_2621 | JN900929 | F:GAGAAACAACAGTCTCTGCTGCTG  R:ACTCACTTCAAGTCTCAGCTGCCT | 57.5 |
| scaffold497_2618 | JN900930 | F:GATCACATCTGGATGGGTCGATAC  R:AGGTTGTCGAGCTCCTGTCAAGT | 57.5 |
| scaffold1468_78783 | JN900931 | F:GGAGATGAGAGCTGCACACT  R:GCAAGTCGGGGAACAGTG | 57.5 |
| scaffold653_3885 | JN900932 | F:TCACCAGTTTCGGTGAACTACC  R:CCATCTGACACACCCAGC | 57.5 |
| scaffold1386_78493 | JN900933 | F:CCAAACCTGGAATCTGATGTGAAC  R:CTCTGCCTGTCAGCTAAACAAC | 57.5 |
| scaffold749_61895 | JN900934 | F:AGCAACAAAGAGGCACAGGTGTAG  R:ATTCAAGAGTGATTGCACGAC | 57.5 |
| scaffold781_27382 | JN900935 | F:CAAGGATGTGAAGCGTGGG  R:TCATAGACTCAGTCACCGGAATGT | 57.5 |
| scaffold781_27363 | JN900936 | F:AGGGATCATTACGCCTGACTG  R:GTCTGTGTTTGGACTTTTGGGTG | 57.5 |
| scaffold571_3089 | JN900937 | F:ATCTGATCGTACCCGTCAGC  R:AGCTCCAAGTCACAATGCGA | 57.5 |
| scaffold315_34921 | JN900938 | F:TCATCTCATTTGCTCCCTGCA  R:GCTTCGAGTGATGTCAACCAAC | 57.5 |
| scaffold750_60314 | JN900939 | F:GCCGGTGTGTCATCAAATAG  R:ATAGAAGTCCCTTTGTCTCCGGTC | 57.5 |
| scaffold315_34907 | JN900940 | F:TGGTGCCGTCTGGATACTC  R:TACAGTGAGCGTGACGCAA | 57.5 |
| scaffold1022_72814 | JN900941 | F:AGCCAAGGTGCTCAAAACC  R:CAATCTGGTGAGAGACACAGGAG | 57.5 |
| scaffold162_19439 | JN900942 | F:CCACAGACACAGAAAAATGCTGAC  R:CCTGTTGACACAGTAAATGCCAGA | 57.5 |
| scaffold232_939 | JN900943 | F:TCCTGTAGGTAACACTGGCC  R:AGATGACGAAGTGGTTACTGCC | 57.5 |
| scaffold876_5433 | JN900944 | F:TGCCAGTGGTCATTTCTGTC  R:TTCTTCGCGGAAACGTTAC | 57.5 |
| scaffold1302_77797 | JN900945 | F:GGTACTCACTAGCACAGGAAGAG  R:TGATGACCTGACAGCCAGAG | 57.5 |
| scaffold232_937 | JN900946 | F:AAACGCAACCAGCATCTGAC  R:GGATCCTACTCTGTAACACCCTG | 57.5 |
| scaffold387_47357 | JN900947 | F:AGGGCCAAAGTCTGGGTCAGTA  R:ATCTCATAATGGTTGGCTGACACG | 57.5 |
| scaffold462_2429 | JN900948 | F:TGCAATTACCTCAGACCCACTTTC  R:GACTCTCCACCAGGTTAACCCCT | 57.5 |
| scaffold184_20694 | JN900949 | F:TGCATTGACACACCCCAG  R:TGACACATTCTTCCGTTTCTGC | 57.5 |
| scaffold424_43876 | JN900950 | F:TCACAGAGTCCTCTGATCACTCA  R:AGTGTGAGTCGTGGCAGT | 57.5 |
| scaffold106_184 | JN900951 | F:TGCAGCATGTGCTTCACTG  R:GTCCTGTTGCACGTTGGTAAC | 57.5 |
| scaffold685_4237 | JN900952 | F:ACAGTCCAATCAACCTCGTCTG  R:CAGTCACACCAGTGCACAC | 57.5 |
| scaffold162_19489 | JN900953 | F:GGCGTCCTGTGTTAAAGCTTAC  R:CCATATGCTCACTCGTTTTCTGTG | 57.5 |
| scaffold558_56416 | JN900954 | F:ATGCTGTGCTGCAAAATATGTGAC  R:GTACAGTATGTGCATGTGTGGGTG | 57.5 |
| scaffold854_67343 | JN900955 | F:GCTGCACAGCAAGTGGAT  R:TGGAGGCCTCACATTAACACA | 57.5 |
| scaffold346_39699 | JN900956 | F:ATCTGGACCAGGATG  R:CAGCTTTAACAACATAAGCCCTC | 57.5 |
| scaffold46_4553 | JN900957 | F:ATCCCATGACTTACTGACCCAGTG  R:TGCAGACTGGAATGTCAGAGG | 57.5 |
| scaffold549_25816 | JN900958 | F:CAATCTACTGCACCACACTCCA  R:TGCAAATTCAACAGCACCCAG | 57.5 |
| scaffold824_65931 | JN900959 | F:CAATTCTGAGGCATTTCAGGGG  R:GAGAGAGGCATGCTGTTACGT | 57.5 |
| scaffold16_2029 | JN900960 | F:ACATTCAGTGACTGCTCAAGCAAC  R:TGTCAGTGAGTCATTGCTCCTCTC | 57.5 |
| scaffold1306_77868 | JN900961 | F:CAGAACTCACCAGCAACCTG  R:GTGCTTGGCTCTCTGAAGG | 57.5 |
| scaffold894_68977 | JN900962 | F:ACCTTAGATGGCCTTACTGAAGC  R:GAGCACACTGACTGGTGTTG | 57.5 |
| scaffold894_68949 | JN900963 | F:TGTCCGGATCTCGATCTAAACCT  R:TGTTGCGCAAACTTAAAGCCA | 57.5 |
| scaffold485_2520 | JN900964 | F:GTCATCTTTTGTTGTGGTCTGCAC  R:TAGTGGTGATCAGCTGCAGGTCT | 57.5 |
| scaffold894_68978 | JN900965 | F:TCAGTGTGCTCCGCTAACA  R:CTGCCAGAGAGGGACAGAT | 57.5 |
| scaffold485_2525 | JN900966 | F:TTTCTTTGTGATGTGACCTTGGGT  R:TGATGATCCTATGCTGAGGTC | 57.5 |
| scaffold485_2521 | JN900967 | F:TCCTCTGTCATTTTTCCACCTTTC  R:GAGGAAATGATAAGGGAGGAAGCTA | 57.5 |
| scaffold894_68941 | JN900968 | F:GCATTTGCCCAGGACCAT  R:TAGGCAAGCTGAGGGAGAC | 57.5 |
| scaffold1525_78936 | JN900969 | F:TCTGCGCCATCCAATTCAAC  R:CTAATGGACACGCTGTGGC | 57.5 |
| scaffold939_70453 | JN900970 | F:GCGGGACTCCTGTTTG  R:TTACACGGCCTCTGAACC | 57.5 |
| scaffold558_26183 | JN900971 | F:GAACAGATTGTGGTGGTCTGGA  R:TCAATGTGGAGAAAAAGAGGGAGG | 57.5 |
| scaffold783_63811 | JN900972 | F:GTGAGGACACAATGCATTCCC  R:CACACCCACAGTGTCAAACAC | 57.5 |
| scaffold768_63055 | JN900973 | F:TCCTCTGATGGTATTGGCCTTG  R:AGAGTTTGACAGTATGCTCGATGG | 57.5 |
| scaffold800_60902 | JN900974 | F:TATCCTTTCTTCCCACTGCTTCCT  R:TAATCAATACAGCGACGACCAAGA | 57.5 |
| scaffold800_5108 | JN900975 | F:TCCTGATAAGAAACAGGTCCGC  R: CAAGTGTTTTCAGCCATCAC | 57.5 |
| scaffold764_4900 | JN900976 | F:GACCACATTTGATTCAACTCTCCC  R:TATCGCAGGTTTTGTATTGTGCAT | 57.5 |
| scaffold800_5098 | JN900977 | F:GGTTTCAGTCACAGCCACAAG  R:AGTGTCCTTAGAAGTCCCTGC | 57.5 |
| scaffold81_3198 | JN900978 | F:AGATCCATAGTGTCCTTGCAGTG  R:GGTCTTGTCCTCAACATGCC | 57.5 |
| scaffold81_3303 | JN900979 | F:CTCAAACACCTCACCAACCC  R:ACCCAGAGAGAGGTGACGA | 57.5 |
| scaffold81_11159 | JN900980 | F:CGTTTTTGGGTGAACTATCCC  R:GTTTAACAAGCCGCAACACCAC | 57.5 |
| scaffold738_61428 | JN900981 | F:TACACAAGGTCTGACAGGGC  R:CAGTGTTGTCCCATAGGCTGA | 57.5 |
| scaffold1306_77884 | JN900982 | F:TACACAAACACACGCACCTTTG  R:ACTCTAGGTGATGAATGAAGCCC | 57.5 |
| scaffold83_10266 | JN900983 | F:CCAAACAAAACAAGTGTTCACTCC  R:CAAGAGCAGTACGCTGACG | 57.5 |
| scaffold406_42810 | JN900984 | F:GATATTAGGGATGTGGGTGTCCC  R:AGCACATGAACAGCTGCCATA | 57.5 |
| scaffold804_64852 | JN900985 | F:ATGACTAAAGCAGACAGGGTGGAC  R:CAATTGTGATGCATTGCTTTGTCT | 57.5 |
| scaffold560_56685 | JN900986 | F:CAACCATCACTCCTCCTCTGTCTAC  R:ACAAAGGAACATCTCCTCTGGG | 57.5 |
| scaffold804_64892 | JN900987 | F:TGCACATACAGCTCTTCAAACACA  R:TCTACTCAGTTTCATCATTCCCCC | 57.5 |
| scaffold418_1966 | JN900988 | F:CAGTTATTTACTGGAAACCGCTGG  R:GTGATCTGGTCGGTTCTTCCTC | 57.5 |
| scaffold936_5228 | JN900989 | F:AGGAGTGTGTTTCCCAAAATGC  R:AATTACCTCGCATGCTGCC | 57.5 |
| scaffold169_20324 | JN900990 | F:GACCCTGGGAGAAAAACAG  R:GGGATCAGGGAAGAGTGACAT | 57.5 |
| scaffold544_25903 | JN900991 | F:GGTTTTCAGCACCATGGACAG  R:AGTTCTGGGATGAACGTGG | 57.5 |
| scaffold319_39135 | JN900992 | F:CACACTCACAGCTGGGTCAGTT  R:GGACGTGTGCATGTGAAATATAGC | 57.5 |
| scaffold544_25910 | JN900993 | F:CTGCTGCTATTTGCTGTCCTG  R:TTGTGTGAAATGTGACCAGACGA | 57.5 |
| scaffold1012_72411 | JN900994 | F:AATAACCTCCCTGGCGGAG  R:GTCTTGAGTCACGGTCCAGTAC | 57.5 |
| scaffold751_61936 | JN900995 | F:CAGTCATCCGTTCAAACTCCG  R:CTGCTGTACAGTTACCAGACGT | 57.5 |
| scaffold125_5743 | JN900996 | F:GGCTAACAGATGTGCGTCTTACT  R:TGGTTGTGGAAGCTGTGC | 57.5 |
| scaffold13_1247 | JN900997 | F:TGAACATAGCATGGACTGGAGG  R:CCATGGTTACCAGTGTGGTACT | 57.5 |
| scaffold1282_77574 | JN900998 | F:GGTACAACTGAGCCCAACATG  R:AGAACTGAATTCCTCTGTGACCC | 57.5 |
| scaffold1380_78434 | JN900999 | F:CGTCACATGTTTCATTTGAGTTCAG  R:AGTCACTCTCCTCTACTGCGGTGT | 57.5 |
| scaffold634_3784 | JN901000 | F:GATCTGTGCGTTAAAAATGAGGCT  R:AGCCTTGTCAGGGACAGGTG | 57.5 |
| scaffold1254_77249 | JN901001 | F:TGAGGACTTCCTGCTCTACAAC  R:CCTCAAACAGCGTACTGACCTA | 57.5 |
| scaffold799_5021 | JN901002 | F:CTTTCCAAAATGGATCCTAGAGGG  R:CAGGAGATATTAGCCTCCATTACCAC | 57.5 |
| scaffold799_5022 | JN901003 | F:TTTTATTCCCTGATGTCCTCAACG  R:TTTACAGTGTTTGGTCTGGTGCAT | 57.5 |
| scaffold296_33277 | JN901004 | F:TCTGATGTAGACAGATTGATGCCA  R:TGAAAGAGTATCGACCAGCTGAAG | 57.5 |
| scaffold937_5294 | JN901005 | F:TTGCTCCGGCTATAAACTTTTGTT  R:TGCTGTGTGATTGTCGTATTAGCA | 57.5 |
| scaffold699_59701 | JN901006 | F:GAAGGAAGAAGTGCACAGAGG  R:CTCCTCGCATATGTACCCG | 57.5 |
| scaffold573_3307 | JN901007 | F:GTTGGAGCGTTTGTTGTC  R:TGAGTGAGATTTCCTCCAATCCAT | 57.5 |
| scaffold573_3290 | JN901008 | F:TTCACAAAACCTTTCAGCAAACAA  R:GTGCACCTGCAGATGTGAGTG | 57.5 |
| scaffold435_2117 | JN901009 | F:CCTGCACATTTTTAATTCCTCTCC  R:TCATATCAAGAATTATTTCAGCCGC | 57.5 |
| scaffold392_18964 | JN901010 | F:GAGTACGAGAAGCGAGTGGAG  R:TATCCAACAGGACAAGGGACC | 57.5 |
| scaffold435_2113 | JN901011 | F:CAGGGTGGTTTGGATTACCCTT  R:GGGTTTTAGCACTGATCCTGGTC | 57.5 |
| scaffold613_3557 | JN901012 | F:CCTGAAGATTACGCTCTGCTCA  R:TCCTTTTGTCTCAGGCTCTCC | 57.5 |
| scaffold423_43798 | JN901013 | F:GGCAGCTCGACTTTACATCG  R:CCACGTCTGTTACCGAAGC | 57.5 |
| scaffold194_23178 | JN901014 | F:TCTTTGAATGGCAACACCGC  R:GTGAACCTGCACTTGAACTTGAC | 57.5 |
| scaffold296_33196 | JN901015 | F:GTGGACGATTACTTGCACTAGCCT  R:ACTACAGGTGGGAGACATTTCTGG | 57.5 |
| scaffold112_13049 | JN901016 | F:AGGATCCTGGTCTGTTGATAATGC  R:ATTTGCCTGCTGAGAAACAATCTC | 57.5 |
| scaffold112_13083 | JN901017 | F:GTGTTCATCCTACCTGGTTTGGTC  R:CTCTCAGCTCCATGGCTTCTTCTA | 57.5 |
| scaffold634_3790 | JN901018 | F:CTGAGCGTCTTGTCAAGCG  R:AGGTCAAGGGTCAAAGTCCTG | 57.5 |
| scaffold1189_5365 | JN901019 | F:CACAAGAATCCAGTCGGCC  R:GTCTGACTCCAGCATTTGGC | 57.5 |
| scaffold392_18868 | JN901020 | F:TACCGTGTGTTCCCAGTGAC  R:CTCCTAACATCAGCCCCTCTC | 57.5 |
| scaffold530_50163 | JN901021 | F:TTCAGAGCTGCAGGGTAGAG  R:TCACATTAGCTGCCAGTTCAGT | 57.5 |
| scaffold689_58890 | JN901022 | F:GGACGACATGACAGCACC  R:CAGCAGAGTGAATGCAGGATG | 57.5 |
| scaffold1218_76711 | JN901023 | F:CGACTCCACTTAGACGTTCTGG  R:AGGAAGAGCTGGTATCGCAAC | 57.5 |
| scaffold916_69613 | JN901024 | F:GGGAAGTTCCAATGAAGGAAGC  R:CAGAGCAGGTGTGTTGTGATG | 57.5 |
| scaffold423_43818 | JN901025 | F:CAGGGTTGACGCGTTTACAC  R:TTGATAGAGTGAGGAGCGACCA | 57.5 |
| scaffold15_1375 | JN901026 | F:TTGCTCTCAGGACATCTGGAG  R:ACGAGCAGATTAACGAGGAGAG | 57.5 |
| scaffold271_13944 | JN901027 | F:CAGACAGTTGCAGTGACATCAC  R:CCAGGACAAAGTTGAGGCTG | 57.5 |
| scaffold114_13593 | JN901028 | F:AGCTAGACAGCTGAGTGAAGTG  R:TTCCTGCAGAGACGTGTGAT | 57.5 |
| scaffold417_43438 | JN901029 | F:AGACGCAGCAGCTCTGAT  R:AGAAAGTTTGTGAGCGTGCAG | 57.5 |
| scaffold194_23196 | JN901030 | F:TGTGTGTTTGCGTGTTCACC  R:TTCTCAGGAAGCATCTGTCTGTG | 57.5 |
| scaffold895_69048 | JN901031 | F:GCACGTTTCTGTCTGGAATCAG  R:AGGTGCTGAAGGAGATGCTC | 57.5 |
| scaffold240_30554 | JN901032 | F:TAAATTAACCATTAGGGCTGCCTG  R:ACAATGTTTGCAAATCAAAC | 57.5 |
| scaffold606_54789 | JN901033 | F:CCATGTTTAATGGCAGTCTCACAC  R:AGCTCTGTCTCATACCAGGC | 57.5 |
| scaffold645_26738 | JN901034 | F:TTTCATGCTCTGCGCTCGA  R:ACATGTGGGAATCAGGTGGTAC | 57.5 |
| scaffold174_7323 | JN901035 | F:GTGGGGAATCAAACCTGTTGAC  R:CAGGTGGGTAGACAGACAGAC | 57.5 |
| scaffold174_7324 | JN901036 | F:GTCTTCCATATCCACCCGTCA  R:GCGTCTGTGGGCTCATAAC | 57.5 |
| scaffold606_54793 | JN901037 | F:ACATGAGAAGTGCACAGAGGG  R:TGATATAAAGCCCAGATGTGCAGG | 57.5 |
| scaffold645_57852 | JN901038 | F:CCATTTCATGCTCTGCGCTC  R:CGTCCATGAATTACGTCCATG | 57.5 |
| scaffold645_26745 | JN901039 | F:CTATGGCACAGACTCAGCCT  R:AAACGGACAGAGAGGGACTG | 57.5 |
| scaffold440_22588 | JN901040 | F:TGACCATCAGCTAAAACCTGTCAC  R:TTCAGTGTGGATGAACCTGCA | 57.5 |
| scaffold390_44483 | JN901041 | F:CTTTTCTCTCTTTGGGCCTGC  R:CTCTGCTGTTTTCTCTTCGTGC | 57.5 |
| scaffold440_22585 | JN901042 | F:TGTTCACCACACAATGATCTGCAT  R:TTCTTCCCTGACCCATAGCAC | 57.5 |
| scaffold1132_75250 | JN901043 | F:AAGGCCATTGCTGGGATG  R:GTCACTCCATATCCTTGTGTTGC | 57.5 |
| scaffold740_61572 | JN901044 | F:CCGCTAATGGAGAGAGTGCA  R:CGGAGGAATCACTATGGGTCTC | 57.5 |
| scaffold221_25716 | JN901045 | F:TATGCATAGCTGCCACTCAGG  R:GCGAGTGTAATCTGATCGTTCGA | 57.5 |
| scaffold221_25699 | JN901046 | F:CCCACTACAGGCGGTATGT  R:CAAGTTCCTTCGGCTGGAAG | 57.5 |
| scaffold480_46992 | JN901047 | F:GAGAATCCACGCAGTGAGC  R:GCTGTTGTCTCTCACATGGAGA | 57.5 |
| scaffold1072_73975 | JN901048 | F:GGATCGTGATTCTGGTACTGCT  R:CTGGCACAGACTCAAACACAC | 57.5 |
| scaffold286_14986 | JN901049 | F:TCATCTACCTTCGACAGCAGC  R:CCATGTCCAGTACCAAAGTCCA | 57.5 |
| scaffold217_10725 | JN901050 | F:CCTGTGTTCGTGGAGTACTGTAGC  R:GAGAGGTGGGGTAAGGCTAGAATC | 57.5 |
| scaffold203_10566 | JN901051 | F:AGCATGGTTTGCGTGGATTG  R:AGAGCAGGAAAGTAGGCCAC | 57.5 |
| scaffold1457_78773 | JN901052 | F:ACACAGGGTTCACCACACA  R:ACATACAGTAACCCTTCTGCCTC | 57.5 |
| scaffold407_42833 | JN901053 | F:AACACCACAGTGACAGGCTT  R:CAAACTTCACACCCACGATTGG | 57.5 |
| scaffold659_3998 | JN901054 | F:GCTGGACATTTCTTGGGACAC  R:ACCTCTGACCTCTGCATGG | 57.5 |
| scaffold692_59140 | JN901055 | F:AACCTTCTCCTGACCTGACG  R:GCAGAACCGAAACAGGAAGTC | 57.5 |
| scaffold97_170 | JN901056 | F:GCTGGCAGTTAACTAATGGAGC  R:TGTCCTTCTGACTACGGCTG | 57.5 |
| scaffold97_169 | JN901057 | F:TGTTCCCAATGGATTTGCGTG  R:GCACCTGTGCTGTAGCTC | 57.5 |
| scaffold276_972 | JN901058 | F:GGAGGTTATTCTCATGTGATTTCCA  R:TGGTTACTGTGAGACAGGTCACTG | 57.5 |
| scaffold276_982 | JN901059 | F:ATCTGGTGAGAGGACCACAGC  R:GCACATTTGTCCTCTGGTTAAAGC | 57.5 |
| scaffold626_3765 | JN901060 | F:AGTATTAAAGTTGGCGTGCTGC  R:TGTGTGCATGAGTGAATGAGTGA | 57.5 |
| scaffold276_986 | JN901061 | F:GGTGATGAGATAAGAAGAAGCAAC  R:TTTCAATGAGACGTCAAGGAC | 57.5 |
| scaffold626_3767 | JN901062 | F:CGAAACATGTGCACAACC  R:GCTCGTCCTAACTCCACTCTCTTG | 57.5 |
| scaffold230_689 | JN901063 | F:GATCTCTGCTTGAGACTAAGCCTG  R:CAGGCCACATCCCAAGATG | 57.5 |
| scaffold230_682 | JN901064 | F:TCTCTACAGCAGGGGTGTTG  R:CCTGACTTTGGCATCCATCC | 57.5 |
| scaffold873_68204 | JN901065 | F:ACCCTGAGCTCTAGGATGGA  R:AGGAACGGGTGGATGTAGATG | 57.5 |
| scaffold914_69548 | JN901066 | F:CATGAGGGATTGACCATTTGTGG  R:TGAAACCCATCTACGACTGCTG | 57.5 |
| scaffold119_13335 | JN901067 | F:TAACATCTCTGTGGTCGATGCTGT  R:CTCAGTGAGCGAGGGTGAGAG | 57.5 |
| scaffold910_69514 | JN901068 | F:TGAAGCATGAAAGCAAGGCC  R:TATCTCTTCATCCTGGCTGCG | 57.5 |
| scaffold31_3953 | JN901069 | F:CTGGTCAGCCAAAGGGTG  R:GCCTCCCGAGTGAATGTG | 57.5 |
| scaffold333_36806 | JN901070 | F:TGGAGGCTGTGCAACATCT  R:AGCAGCAGCATTGAGTGATTTG | 57.5 |
| scaffold873_68203 | JN901071 | F:AGAAGCCAGTCAGACAGCAG  R:GTGACTACGCCACTTGCG | 57.5 |
| scaffold1119_74976 | JN901072 | F:GTCATTTTGTCCCGCAGTCTC  R:AGGCCAACTGTTTCACAAAGG | 57.5 |
| scaffold197_23694 | JN901073 | F:GGGGTTCAGTATATTTCCCTCGTC  R:AAGTGCTGCCATTGATGAGATAGA | 57.5 |
| scaffold826_5003 | JN901074 | F:AGGCCAGATTACAAGTGCTACAAG  R:AGGTGGGTTTCAGTCCAGTC | 57.5 |
| scaffold274_15498 | JN901075 | F:CTGAAGATCGTGAGGATGGATCG  R:GGTGGAGCTGTTGTCAAACAC | 57.5 |
| scaffold962_71125 | JN901076 | F:GACTACCTTACTGAGCGAGGC  R:GATTTGCTGTTCACTTGTACCCG | 57.5 |
| scaffold896_69079 | JN901077 | F:GGTCCAGACACTAATCAGTAAATGCTC  R:CAACCGTCCTCACATTAATG | 57.5 |
| scaffold274_35443 | JN901078 | F: TGATGCTGTCTCCGTGTGT  R:CTGGGAATCCTCTTCTTCTTCCAT | 57.5 |
| scaffold344_37486 | JN901079 | F:CATGATGACGCTGAATTACCTGC  R:TCGGGTTTACAGAAGTGTTGAGTG | 57.5 |
| scaffold921_69859 | JN901080 | F:AGGATGTTAACTGGTGAGTGACCG  R:GAGAGGATTCTGGGCTTTTGTGTA | 57.5 |
| scaffold481_23903 | JN901081 | F:GGTAGGCAAATAACGACTTGATGC  R:TGCACTGAAAAGACTGCACCT | 57.5 |
| scaffold224_26315 | JN901082 | F:CACAATGTCGGAACTAATCTGTGC  R:CGGAACTTGTGGTGTGATCTTATG | 57.5 |
| scaffold44_5253 | JN901083 | F:GTTTGAACTGCGGATTTAGGAATG  R:AGACAAACCCAATGGCACA | 57.5 |
| scaffold921_69868 | JN901084 | F:GATGGCAGATGGATGATCTAACCT  R:CGCCTCTAAAATTGTTGGATTCAC | 57.5 |
| scaffold1585_79029 | JN901085 | F:TCACAACAAGTCTGTCTCTCTGGG  R:CTAGTCCATCACTCCCTTGTCTGC | 57.5 |
| scaffold1326_78007 | JN901086 | F:GATCTGCAACCATCAGCTG  R:CTCAGGACCGTCTCCTTCAG | 57.5 |
| scaffold210_24614 | JN901087 | F:TCTTTGGAAACCATCGCATC  R:CAGGCAAAATGTGAAGAACACATC | 57.5 |
| scaffold784_63815 | JN901088 | F:CAGTGCAAAGACAGGTGCTAAC  R:TGTATTGTTGGGCCACATAATCCTC | 57.5 |
| scaffold812_65243 | JN901089 | F:TCCTTGTGTCAAGTGCTGATGT  R:ACTTTGTGGTTATGGCCGC | 57.5 |
| scaffold784_63814 | JN901090 | F:TCAGGCCTACAGCATCATCAC  R:ATCACGACCTTGGGCATTTTG | 57.5 |
| scaffold666_57505 | JN901091 | F:ACTCATAGTCACCGAGGGC  R:TCCTCCTCTGGTTTCATGACG | 57.5 |
| scaffold587_1462 | JN901092 | F:GACATTCACAGGTTGCACATTG  R:GACCGTCATTAGGCCAGAGAG | 57.5 |
| scaffold481_23897 | JN901093 | F:ACTTGCTGAGTTTGCTGGTTG  R:CCGTCTCTCAGATCCACACTG | 57.5 |
| scaffold977_71416 | JN901094 | F:TGAACAAGCAGGAGCGTCA  R:GCTACGCTAAGCACCGAAG | 57.5 |
| scaffold619_57730 | JN901095 | F:GGATATCCCTCAGCTTATCCTCC  R:TGTATCCCCACATAATCAAAATG | 57.5 |
| scaffold1180_76252 | JN901096 | F:CTTGCAGGAGCAGTCGTC  R:CGGCAACATCAGGGTGAAG | 57.5 |
| scaffold1180_76253 | JN901097 | F:GTCCTTTGCAGATGTAGACGC  R:GTCAGGAGGATCTGGCTGT | 57.5 |
| scaffold962_5314 | JN901098 | F:CATAAGGAAGACAGGTCCCCA  R:CACTGAGAAGCTGTGAGCG | 57.5 |
| scaffold224_26330 | JN901099 | F:TGCATGGTGGAAGGTTTAGT  R:ATACGTGCCTTGTGGCTCA | 57.5 |
| scaffold1010_72348 | JN901100 | F:CGTCCTGTGGTCAAGTGATTG  R:GCAAGTTTGGGCAGATAGGAG | 57.5 |
| scaffold771_63300 | JN901101 | F:TCCCATGCTATTCTGAAGGCTC  R:AAGGGAATCACTACCACTGCTG | 57.5 |
| scaffold393_41196 | JN901102 | F:TGAGTGTGATCAGGGGTGATG  R:TGAGCAGCTTGTTAGACCTCC | 57.5 |
| scaffold274_15496 | JN901103 | F:CAGGATGTTCAGGGTCGTCAT  R:AATCTATTCCAGATGGGTGTGGC | 57.5 |
| scaffold320_35214 | JN901104 | F:TGTTTCTTACAGGGGGTCCAG  R:CACCCCACATTAGGCAAAAGTC | 57.5 |
| scaffold458_23153 | JN901105 | F:TTCTCAGCTCCACAATCCTCAG  R:GTCCAATCACTCCGCCGATAT | 57.5 |
| scaffold394_41231 | JN901106 | F:CACTCAGCGCTGTTAATTGGTG  R:AATCCAGATGGGAGAAGCTCG | 57.5 |
| scaffold730_27331 | JN901107 | F:CTTGGCAGAGGTATGAGAGGTAC  R:CTTTGAGCCGTCATGTTGATGTG | 57.5 |
| scaffold801_64596 | JN901108 | F:GCAACCTGACATTCACGTCC  R:GCAGTCATGTGACAGTGAAGC | 57.5 |
| scaffold458_23132 | JN901109 | F:TGTCCTGCTACAGGAGACATCT  R:ACTGAGAGTGCACTGGTCC | 57.5 |
| scaffold1086_74307 | JN901110 | F:CGTGCAGTAGCCATATGTGC  R:TGAGAGCCTGCAGAGACAC | 57.5 |
| scaffold1086_74309 | JN901111 | F:GGATCCAGAACTTTGTGCAATGG  R:GTGTCACAGCAGCAGGTG | 57.5 |
| scaffold1191_76393 | JN901112 | F:TCTCTCCTAACAACATATGAAAGCCA  R:TGGACTATGTTTGGTTAATGTTTGTGT | 57.5 |
| scaffold301_34693 | JN901113 | F:CTTGGTTGTTTAGCTACGAGCGAT  R:GACCGTGGTCACTTTATACATTTCAG | 57.5 |
| scaffold623_3660 | JN901114 | F:GCAGAACATCCACCTCAGTG  R:GCTTTGAATTGTGCTGTTCGC | 57.5 |
| scaffold468_46442 | JN901115 | F:TCCTCTTCACGCATAAGAGAGC  R:CAAACAACTCGAAACCCACCTC | 57.5 |
| scaffold350_40278 | JN901116 | F:CGAGTGCAAAATAAACGCAC  R:CACCCATAAGAGAAACACAGCTCA | 57.5 |
| scaffold538_50478 | JN901117 | F:TTGATACCGTACACCTCATCTCCA  R:TGAAAAGAGAGAAAAGGAGCC | 57.5 |
| scaffold1433_78685 | JN901118 | F:GGCAACACTGAGCTACCAC  R:CTGATTTCTGCAGCCGAGATTC | 57.5 |
| scaffold383_42014 | JN901119 | F:TTGCTCTGTTAAACGACTCTGGCT  R:GTGCTCCTTCCCTTCTATGTGCTA | 57.5 |
| scaffold224_26328 | JN901120 | F:GGATTGGATCTGTCATATCGGG  R:ATACACTGGTTTAGCGCATGTG | 57.5 |
| scaffold364_18000 | JN901121 | F:GAGTCACTTCTGGTGCAGGT  R:TCGTCTGTAACGGTGGAGC | 57.5 |
| scaffold403_20874 | JN901122 | F:CAAACTCTGCCGGACTTGG  R:GTGACTGAACAGAGGCTGC | 57.5 |
| scaffold1386_78494 | JN901123 | F:GTCCTGGCAATTTGTTAAGTTTGC  R:GGGACTTTAGAAGAAAGGCACCAT | 57.5 |
| scaffold352_38271 | JN901124 | F:TCAGTTTCTGCTGCTCAGAGTG  R:CCTGAATCTCATGGACGTTTCCA | 57.5 |
| scaffold623_55491 | JN901125 | F:ATTTCCTTAATCTAAACCTGGCCC  R:GATATGGGATATGCAGCAGAAC | 57.5 |
| scaffold812_65250 | JN901126 | F:TGTCCTCATCCGTCAACTCAG  R:CTGACGCAACTGAGCATCTTG | 57.5 |
| scaffold332_36656 | JN901127 | F:TACCGTGTCTCCTGTGAGC  R:GGATCGACTGTGTGTTGGG | 57.5 |
| scaffold1290_77648 | JN901128 | F:GTGGTGAGTGTGAGGTTTGAG  R:TGTGAGAACTCGTCATAAGCGG | 57.5 |
| scaffold791_5158 | JN901129 | F:TCTGGTGAGTCCAGAAGTTGC  R:CCCTTAGCCGTCACTGGAA | 57.5 |
| scaffold915_69571 | JN901130 | F:CCTTGACAGCGCTGAGA  R:GTGAGTGTTGATCAGATTGTGCTG | 57.5 |
| scaffold791_5157 | JN901131 | F:TCTCTGTGATCTTTGACACCGG  R:GCAAGTGTCCTGATCCGTAGT | 57.5 |
| scaffold791_5155 | JN901132 | F:GATCACCTCTACCTTCGAACACC  R:AGAATCTGCTGAGTCGCTCC | 57.5 |
| scaffold1013_72464 | JN901133 | F:GCGCTGCTATGTAGACGC  R:CCTGAATCCTCGACTGCTGA | 57.5 |
| scaffold327_17053 | JN901134 | F:AGCTAAAGAGATGAACCCGAGC  R:GTCAGGACGACTCACAGAGAATC | 57.5 |
| scaffold1131_75238 | JN901135 | F:CCTGCACACAGACTCAAACC  R:GCACCAAACTGCACACACT | 57.5 |
| scaffold327_17066 | JN901136 | F:GGAGGAGTCGTGGTACTGAC  R:GCCGACAAGTTGTGTGTGAG | 57.5 |
| scaffold650_3947 | JN901137 | F:CAGACTAGCAGAAAGCTGCC  R:CCTATCAGAGGACACGAAGAGC | 57.5 |
| scaffold204_11374 | JN901138 | F:CAGAAACAAGACGTCGCACAG  R:GTAATTGTCGTCCTCGTCCACA | 57.5 |
| scaffold915_69572 | JN901139 | F:TCAGCCAAGATGTGTCTCAGTAGC  R:AACAGCATTTACAATCAACGAGCC | 57.5 |
| scaffold915_69566 | JN901140 | F:CTCCCTCTGTGCTCCTTTACACAT  R:TAGTGGCTTGTATGGCTTGTTCAG | 57.5 |
| scaffold746_4565 | JN901141 | F:AGAATGAGCAGCTGCAAGC  R:TCGTAAGTGCTGGTATGTGTGG | 57.5 |
| scaffold337_37043 | JN901142 | F:AGGATATGAAAGGGGGTTTGTGTT  R:TTACATAAGCAGTAGCCAGGCACA | 57.5 |
| scaffold458_45726 | JN901143 | F:CACACCTATTGATCCTCGGAGAC  R:AAAGACACCTCGGAACAGGAC | 57.5 |
| scaffold892_5130 | JN901144 | F:GGGATGAATGGTTGTCAAG  R:GCACCGGTGTAATCTCAAGATAGG | 57.5 |
| scaffold268_13117 | JN901145 | F:AGGAGGAGAGTCCAACAAGC  R:GTGTGTGCACTTGGTGTAACG | 57.5 |
| scaffold1343_78230 | JN901146 | F:TCTCAAGTACGACCCAAGTGG  R:AGCCCAAACTCTCCTCTCG | 57.5 |
| scaffold263_29884 | JN901147 | F:CCTGGTGCACTGGAAGTTG  R:AGAGCACGTTACCATACCAACC | 57.5 |
| scaffold1060_73810 | JN901148 | F:GTGCTCATTTCTGTGTTCCTAGC  R:GCTGTTATTGCTGCTCTGTAGG | 57.5 |
| scaffold268_13141 | JN901149 | F:GAGGTATCAATGGAGGGGAGG  R:AAAGACAGTCTGGCAACCAACA | 57.5 |
| scaffold1343_78231 | JN901150 | F:AACACCTGCAAGCTTCACTG  R:CAACCAGCTGGCACATTAACA | 57.5 |
| scaffold1343_78232 | JN901151 | F:TGTCGTCCAGCTCAACACA  R:AGGGTCCTGTCACATTGGATT | 57.5 |
| scaffold281_1004 | JN901152 | F:GCCATGGCTTACTACGAACTG  R:GCTGTGTAACCTAAGATGACCCT | 57.5 |
| scaffold281_1016 | JN901153 | F:GTTTTAGTGGAATGGCACGACAG  R:TCTGACATCGCCTGACATCTG | 57.5 |
| scaffold1154_75679 | JN901154 | F:GGTCTCTCGGTCGAGTTTTCCTAT  R:TTTTGGAAATATGCGTGTTCATTG | 57.5 |
| scaffold535_55676 | JN901155 | F:GTCCATCTGGACTTTGACGAAAC  R:AGGAACAAACATGTACCCGC | 57.5 |
| scaffold534_2941 | JN901156 | F:CGTCCAGTTATTACTACGTCTCTCCC  R:AACCGACAATGTGTGGAAATCAG | 57.5 |
| scaffold1147_75471 | JN901157 | F:GATCGGACATGCGAGTCTGT  R:GCACAACGACAAATGCTCAGTAC | 57.5 |
| scaffold1070_73967 | JN901158 | F:CCGTCTAAGGAATGTCTCAGGTG  R:TGTCGGTTCAAGCTGCTGATT | 57.5 |
| scaffold268_13116 | JN901159 | F:ACGAATCCACAGATACAGACAGC  R:AGAATAGAGAAGGATTGCTCCGC | 57.5 |
| scaffold501_2662 | JN901160 | F:TCGTGGTCAAATAACAGAGAGATGTT  R:TTCTTCTTCTTTTGGAACACCCTG | 57.5 |
| scaffold78_9895 | JN901161 | F:TGATGGTGGAGTGCACTGT  R:AGGGAACACTTGCAAGATCTCA | 57.5 |
| scaffold501_2663 | JN901162 | F:CAGTGCAGTAGACATTAGGAGGGG  R:AAGGCAGTGATGGAATTTGTGTTT | 57.5 |
| scaffold546_2975 | JN901163 | F:ACAAATGAGGCTCGACCGT  R:TGATACCACCTTGGATTAGCAGG | 57.5 |
| scaffold725_60932 | JN901164 | F:TCCTCTTGTTGTGTACTCCTCTTTCTC  R:CTCATCTCCACTGAATCCCTTGAT | 57.5 |
| scaffold511_54959 | JN901165 | F:CACAAACTGACCTGAGACCTGATG  R:CTTATTTCACCGGAGAGGACACAC | 57.5 |
| scaffold988_71725 | JN901166 | F:CACGCTCTTCAGGAAGTCC  R:CCACCTTGTTGACGTACACG | 57.5 |
| scaffold988_71734 | JN901167 | F:TGAACGAGCTGAAAGTCTCTGG  R:AGTCAATTAAGCCGCATCTCCA | 57.5 |
| scaffold546_2973 | JN901168 | F:CACTTCGAATGGGAGGCAG  R:CATGGAAGACGTCAACCCAC | 57.5 |
| scaffold211_24698 | JN901169 | F:GGCATCAATATTTCAGTGGACGG  R:TCCTAAAGACTCAAGCGACGAC | 57.5 |
| scaffold728_60506 | JN901170 | F:CACCTTCAAATATGGAAGCTCACC  R:CTGTAAGCAAGAAGCCAGCTCAC | 57.5 |
| scaffold470_46572 | JN901171 | F:CAGCCGGAGTGATATGGATGT  R:TAGGACACTGAGGAGCACG | 57.5 |
| scaffold1158_75822 | JN901172 | F:TGATTAAGGATTTCCCTGCCTGTG  R:TTAGTTGCATGCTTTGGCTTGG | 57.5 |
| scaffold1944_79162 | JN901173 | F:TGATTAAGGATTTCCCTGCCTGTG  R:TTAGTTGCATGCTTTGGCTTGG | 57.5 |
| scaffold84_10294 | JN901174 | F:GCCAAGATTCCCTGATGCTG  R:CAGAACACGTAGCCGCAG | 57.5 |
| scaffold604_3423 | JN901175 | F:GGGATTTACTCCTGCACCGTA  R:TCTTGCTGTCTGAACTCCACC | 57.5 |
| scaffold741_4639 | JN901176 | F:TTACTGTCTCAGGCCCTTATGCTC  R:ATGTAGAAATAGCTGACGTTCCCG | 57.5 |
| scaffold511_25695 | JN901177 | F:CCATTAGGCAGACAACCTGGA  R:ACAGCCAATCAGAGAAGGAGG | 57.5 |
| scaffold860_5128 | JN901178 | F:ACATCAAGGCTCGAGTGTGAG  R:CTGAAGACAAACAGCGACAGC | 57.5 |
| scaffold198_609 | JN901179 | F:ACCTGACAACACTGGATGAGG  R:CATGTTGCCCTGTGGATAACC | 57.5 |
| scaffold860_5129 | JN901180 | F:CCAGAGAATGTGACGGCTTG  R:CAGTATCAACTGTCAGTGTCACTCC | 57.5 |
| scaffold486_24150 | JN901181 | F:CTCCCATTAGAGTTGCCTGCT  R:CCATCCAGCAGAGTACAGCT | 57.5 |
| scaffold1009_72318 | JN901182 | F:GGATACAGTATGTCAATCGCCCA  R:TCCACGAGTTCTTTCACTGATCG | 57.5 |
| scaffold442_44562 | JN901183 | F:GAGGAAACCTGCTCATGAGGCCTG  R:GTGACTCCTAAACAAGAAGGG | 57.5 |
| scaffold18_1881 | JN901184 | F:GCTGCTTCTTTCCCCTTTAATG  R:TTTCCGAGAAGTGACACAAC | 57.5 |
| scaffold129_216 | JN901185 | F:CAGCTGAAGTTTGTGTGGATCG  R:CTTCAGTCACTGTGCCAAAGG | 57.5 |
| scaffold861_5185 | JN901186 | F:TGTCAGAGCCGGAGTCAC  R:GCTTCAGTGTGATCCATGCTG | 57.5 |
| scaffold659_57281 | JN901187 | F:CTGAGTTGCTGAACCACTTCG  R:GGTTATCTCATCCAGCAGACGT | 57.5 |
| scaffold520_25100 | JN901188 | F:TACTCTCTGCATGTGACCACG  R:AGGCATCGGATATACAAGCCC | 57.5 |
| scaffold333_37444 | JN901189 | F:CTGACTCACACACTCATGATCGC  R:TTTATAAGCCATCAACCTGCCAAT | 57.5 |
| scaffold771_63294 | JN901190 | F:AGTTGGTGCATGTGTCTGC  R:TATCCCACCATCCCAGTTGAG | 57.5 |
| scaffold621_55418 | JN901191 | F:CGGTCTGTGTAACTTCATCAGC  R:AGTGGCTGGAACACAAAACC | 57.5 |
| scaffold520_25094 | JN901192 | F:TGGTTAGAGGATGACCTGCC  R:CCTGCAATTACAAGCTCTGCTG | 57.5 |
| scaffold621_55384 | JN901193 | F:TACATGCTGTACATGAGGGGG  R:TATCCAGCCTCATACAGGGGA | 57.5 |
| scaffold158_7542 | JN901194 | F:TACACATCTTATCAAGCCCACTGC  R:CTGTTTTCTACTGGCTGCAACAC | 57.5 |
| scaffold470_46577 | JN901195 | F:TGATGAGAATCTACACAGCAGT  R:CACATGATCTCATGGGGC | 57.5 |
| scaffold1724_79116 | JN901196 | F:ATCATACCTTCCCCTGCTAACACA  R:GCACGCACACTTTATGTCATTTCT | 57.5 |
| scaffold336_38631 | JN901197 | F:GCTTCCAACTGCATCCAAATTAAC  R:GCTGTACAACCAACCAATCAG | 57.5 |
| scaffold241_32740 | JN901198 | F:CTCAAAAGTCACGCCTCAAAGTCT  R:ACCTGCTTGATGCTAGTGTAAGGG | 57.5 |
| scaffold129_16059 | JN901199 | F:CATCATCCCACACAGGGTG  R:TGGTCATCAGGATGAGGAGC | 57.5 |
| scaffold334_37809 | JN901200 | F:TGTTCTTTCTCTCGTGGTGGAATC  R:AGGGCAAGAAATACAAAGGAGACC | 57.5 |
| scaffold334_16323 | JN901201 | F:AGTGAACTCAATCTGGAGCTTTGG  R:TTTAACCATGGGAGAGGTGGG | 57.5 |
| scaffold179_25109 | JN901202 | F:AGTCCTTCAAAATTTACGCACGTC  R:GGAAAGCCATGTTATTCCAACTGA | 57.5 |
| scaffold843_66608 | JN901203 | F:TGTGTGAGGGCGTGTAAGG  R:CCAAAGGAACCAGACACCTACAG | 57.5 |
| scaffold179_24828 | JN901204 | F:AAGAGAGGGAATCAGGACAGCAG  R:TTACAGATGGATGGAAATGTGGTG | 57.5 |
| scaffold60_6605 | JN901205 | F:TGTAAACGAGTTCTTCTTACCAACAGGA  R:ACGCCGCTGGTTTCATTGAT | 57.5 |
| scaffold1030_72976 | JN901206 | F:ACTTCAGGGTGCAGTTTGC  R:CGATACGTAGGAACAACAGTGGT | 57.5 |
| scaffold541_56017 | JN901207 | F:ATGACAGGAACAGAAAGGGAGTTG  R:GTACAGTGTTGTAAGGCGAAAGGC | 57.5 |
| scaffold471_23859 | JN901208 | F:ACAGCTCGACAACATGGCT  R:GCGAGGAACCTACAGAGACAG | 57.5 |
| scaffold749_27846 | JN901209 | F:TTTTGCCTGCTAATGGATGCC  R:ACTCAGAGGCAGGGATGTG | 57.5 |
| scaffold749_27845 | JN901210 | F:AGCCCTGCTATATACTGGCG  R:ATTGTGTGATCGAACGTCCAAGT | 57.5 |
| scaffold749_27843 | JN901211 | F:TCAAGGTCATCCTGAGCCATG  R:TCTCTGCTGTGATGGTTGTCTG | 57.5 |
| scaffold749_27847 | JN901212 | F:ATATGTCAGATGCATGCCAGGT  R:GTGGGAACTGTTGGGTTTCTC | 57.5 |
| scaffold471_23857 | JN901213 | F:GGAGGAGACAAGCTCTCATCAAGA  R:AGCCCAGAGTTTCTGCCTTTAGAT | 57.5 |
| scaffold397_1752 | JN901214 | F:GAGACAAGACGAGACAAGGAGGAC  R:TCTCCTTAAAGCTACCTGGCTCCT | 57.5 |
| scaffold533_50338 | JN901215 | F:AAGTGTTTGCTGCAGGGTTC  R:GCTGTCAAACATGGAAACTAGCC | 57.5 |
| scaffold395_41445 | JN901216 | F:CAAAGTGGAGATTGGACGCAG  R:CCAAGCCTGAAGAACAGCTC | 57.5 |
| scaffold395_41352 | JN901217 | F:GGCGTTCTCTCAGCCAAG  R:CACATTCTCATCAGCGTGGG | 57.5 |
| scaffold611_55043 | JN901218 | F:TAATCCACCCAAGGCCAGAG  R:TGGCAGATTGTTCTGACACACA | 57.5 |
| scaffold64_6455 | JN901219 | F:TGATGAAGTCAACAGTGTTTTAAGAGTC  R:CCCTACGAGTAACACACAGGTCAG | 57.5 |
| scaffold611_55030 | JN901220 | F:TCAGGTTGAGGAGGGAAGTG  R:TCCGAACAAATACCAGGTGTGT | 57.5 |
| scaffold345_39354 | JN901221 | F:CGCCGCACATTAGGAAATGAT  R:CCCTCCCTCTCTTGTCTTTAATGC | 57.5 |
| scaffold471_52013 | JN901222 | F:TTATTACTGAACCTGACACAGCCG  R:ATGTTTGTCTCCCACGCAGAG | 57.5 |
| scaffold843_66583 | JN901223 | F:CTGGTGGAAATGGATGGTTTAG  R:GTTTGGAAGCTGACCTCATCG | 57.5 |
| scaffold667_57614 | JN901224 | F:CTGTTCAGTGCCTGTGAAGC  R:CTCCTCAGCAGCAGTTTGG | 57.5 |
| scaffold972_5340 | JN901225 | F:ACACATCACCTGCAGTTTTGAATG  R:TTGGGTGGATTATGTAGGTGTGTG | 57.5 |
| scaffold833_66211 | JN901226 | F:CCAGGTATCGGTCCAGGT  R:GCTGCTGTCAGACATTCACTG | 57.5 |
| scaffold843_66624 | JN901227 | F:CCAGCATGAGAAGAGGAATG  R:CACTGAAGGAAGGAGGCAG | 57.5 |
| scaffold890_68715 | JN901228 | F:ATGAAGCTGTTTGTCAGTGAGCTG  R:ATATGTGTTTGCCTTCCAAGCTGA | 57.5 |
| scaffold1143_75442 | JN901229 | F:GGTATAGCTTGGCAGCACAATG  R:CCTGTGGATGAAGGCTAATGC | 57.5 |
| scaffold1304_77809 | JN901230 | F:TGGGAAATGTCAGTCCACTCAG  R:AATCCAGTGGAAGACGAAGGG | 57.5 |
| scaffold473_56994 | JN901231 | F:GATCACAAACCATCAGAACTGAGC  R:CAGATCAGCTGACTCCCTATGATG | 57.5 |
| scaffold425_22250 | JN901232 | F:ATGTTTGAAGGAGCACCAGCT  R:GTACTTGTTTGAAGGCATCGCTG | 57.5 |
| scaffold321_35322 | JN901233 | F:CGTCACTCAGAACACGCAG  R:TGGAAGTAGCAGGTTGAAGCA | 57.5 |
| scaffold609_54901 | JN901234 | F:GTATTTCCCATGTTGCTTTGCTGT  R:AAGTGATTCAAGTGAACTTCGGGA | 57.5 |
| scaffold1211_76655 | JN901235 | F:TCTCCATCTTGGTGTCCTGTG  R:TGTGAAGGTGTCGCTTCAGT | 57.5 |
| scaffold117_14552 | JN901236 | F:AGAAGAGGCCAATAAAAGAGCAGAG  R:ACATTTCAATTTGCCCCATAAGCC | 57.5 |
| scaffold419_43528 | JN901237 | F:CTCTGCCAGTCTAGCCAAC  R:TTGTGTGGCTTATAGTGCTGC | 57.5 |
| scaffold419_43505 | JN901238 | F:AGTGGAACATTATGAAGTCTGGT  R:TCGGCATAATGGCTGTGA | 57.5 |
| scaffold419_43475 | JN901239 | F:CCAGCCACCAGTTATTAATGCG  R:CTTTATGGCTCCCTGTGGAAAC | 57.5 |
| scaffold641_3823 | JN901240 | F:ATGCATGCTTTTTCTCAGAAGTCC  R:TCTTGATGAACACTCAAAACACTGC | 57.5 |
| scaffold641_3822 | JN901241 | F:CAGCACCGACATCTCTACTCAAAG  R:ATAAAATGTCCTCAGTGTTTCCCC | 57.5 |
| scaffold452_45014 | JN901242 | F:GCAAGCTGGACACTGATGG  R:GGGTGCTCTGAATGATTGCAG | 57.5 |
| scaffold103_3162 | JN901243 | F:GCTGACTCTGTCTTCCTACAGG  R:AGTGAAGGCAGGTGTCTCAC | 57.5 |
| scaffold1260_77375 | JN901244 | F:GGGAACTATGGCTGACCACA  R:ACATACCACACCATCCAGTTTCAG | 57.5 |
| scaffold945_70598 | JN901245 | F:GGATCGGCCATTAGATGCAC  R:ATGTGGTCACATTCAACCAGGA | 57.5 |
| scaffold200_23990 | JN901246 | F:TGTCGCTCAAAAGTCCAGACA  R:CAGAGGCACGCTGTGTTAG | 57.5 |
| scaffold103_11003 | JN901247 | F:GGGTCAAAGTAAGACAGGACTCACA  R:TTATCCTTACATCAGGTTTGCACG | 57.5 |
| scaffold34_4071 | JN901248 | F:GCTCTGTTCTGTCGCCTTC  R:ACATAAGCGCAACAAAGCTGG | 57.5 |
| scaffold280_32222 | JN901249 | F:AGTTTAAGCAATCAGCTGTCGTCC  R:CCAAGGTCTGAATCCAAATCATCT | 57.5 |
| scaffold164_7266 | JN901250 | F:GTTCACAAGAATGGGGCAGTG  R:CTGCTTCGTCAGTAGCAGGA | 57.5 |
| scaffold443_22400 | JN901251 | F:GCATCAGAGTGAGCTACAGGT  R:TGATGCTTCTCACTTGATCCACAC | 57.5 |
| scaffold725_4613 | JN901252 | F:TGGTGGTCATCATATACAGTCAG  R:CCTGAATCCATGATAGATGTAGATGTC | 57.5 |
| scaffold725_4620 | JN901253 | F:GAGAGATCCTCTGAGGGACTGA  R:GTGTGGTATACGCCATGTTTGTG | 57.5 |
| scaffold657_59041 | JN901254 | F:CAAACACTGCTCTCTCCCTCTCTC  R:ATCACGGGTGTTTGCCTAAT | 57.5 |
| scaffold430_21371 | JN901255 | F:TACCTGCCAGATGACACCC  R:CGACAATGACGGAGTGACCTT | 57.5 |
| scaffold668_57772 | JN901256 | F:CTGTGTGTGTGATGACGTTGC  R:TGAATGGACTGAGTCATCAGCATG | 57.5 |
| scaffold433_44248 | JN901257 | F:CGGATCGGATGAGCAGATAGAG  R:TGATCCCAAACAGTCTGGAAGAC | 57.5 |
| scaffold531_25324 | JN901258 | F:AGCTATCTGTTCACCCTTCATGG  R:TTGTCCACATGCTCTCAGCA | 57.5 |
| scaffold151_6576 | JN901259 | F:TGACATGAGGGTACGAGCG  R:GGAGGATACTTGGAAGCAGCA | 57.5 |
| scaffold151_6583 | JN901260 | F:TCACTGGACCATCACCAAGG  R:CCATCGCCTGTGGTGAATG | 57.5 |
| scaffold151_6580 | JN901261 | F:GCAACACGATGTTTATAGCAGCG  R:AGTGGACTGAAGACCTGCTG | 57.5 |
| scaffold951_70894 | JN901262 | F:CACTGTTGTCTCTGTGCGTG  R:GTCATTTCCCCGACCTGC | 57.5 |
| scaffold1601_79046 | JN901263 | F:GCGGACTGATCTATCTCTGTGTG  R:CGCGTATTTGGAACAAGTGAGTG | 57.5 |
| scaffold282_15132 | JN901264 | F:AATTCCGAATCAGCCGAGTTGA  R:TGACCACGTCTGAAGGAGG | 57.5 |
| scaffold17_1776 | JN901265 | F:GCGACATCTGTACAAGCACG  R:AGCATGGTTCTGCACATTGG | 57.5 |
| scaffold221_673 | JN901266 | F:TACAACGGGAGTATTTACGTGCCT  R:ATTTAAGGGTCCCCTCCTTTC | 57.5 |
| scaffold147_15618 | JN901267 | F:GCCTTCACGATCATTCTCATC  R:GCCAGTGATCTATGACGTCTGG | 57.5 |
| scaffold280_32215 | JN901268 | F:AGGAACCTAAACGTGGAACC  R:CCCCTCTCTGCTTTCTTTTTCTCT | 57.5 |
| scaffold577_56325 | JN901269 | F:CTCGACCTCGATCTGCATAC  R:GGCTGGTGAATACCACTGC | 57.5 |
| scaffold1114_74878 | JN901270 | F:TTTCCACGTAAACAAAACTGGAACT  R:CAGGAGACGACGTACGAGATGTT | 57.5 |
| scaffold766_62965 | JN901271 | F:GGCTGCTAACAAAGAACGACAG  R:CACACACACACAGCGAACTG | 57.5 |
| scaffold766_62971 | JN901272 | F:TAACACGCGTGACGTCTTGT  R:AGCTGTGGCTGCTTTTGTC | 57.5 |
| scaffold707_4384 | JN901273 | F:GATTCACCGAACCAGAACGTG  R:CTCAAAGTGCATGAAGACACATCC | 57.5 |
| scaffold707_4386 | JN901274 | F:GCTGTGGTGTACGGACTG  R:TCCAGGCAAATGGTTTGCTG | 57.5 |
| scaffold905_69394 | JN901275 | F:ACAATGCATGAGACGACTGAATGT  R:ATCTGTGTTTTGTGCATGACAGGT | 57.5 |
| scaffold117_14494 | JN901276 | F:CGACTCAAACCAAAGCTGCATATC  R:GCGATAACCATGGGAACAGC | 57.5 |
| scaffold198_23689 | JN901277 | F:GACGATAGAGACACTTTGGCTGT  R:CCCACAGATCACGTCTGC | 57.5 |
| scaffold1114_74931 | JN901278 | F:TGCACATGTACAAAGGTTTGATG  R:GGGCCGTTAATATCCGTTAATG | 57.5 |
| scaffold1114_74869 | JN901279 | F:TAAACACCAAATGGTGCCTGTTTC  R:CGCTTTAATGAATCTCAAACTGCC | 57.5 |
| scaffold24_239 | JN901280 | F:GGTTTCAACCAGATGTTCCCC  R:AGGATGTGAACAGCGTCTGTATC | 57.5 |
| scaffold314_1157 | JN901281 | F:AGGTGTTCACAGGATCAAAGTCC  R:ACGACAGGAACGACAGGAAC | 57.5 |
| scaffold298_33403 | JN901282 | F:ACACCAGCCTTATGACTATGGAC  R:ACACTTATTATCTCCAGCTCATGCAC | 57.5 |
| scaffold61_6409 | JN901283 | F:GGTGCCAGACAAATTGAGG  R:TGCCAGGCTGTGTTCTAAATAC | 57.5 |
| scaffold115_14087 | JN901284 | F:GATCCAACCAACCTGCTAGGT  R:ACAAACCAAAGGAGCAACACC | 57.5 |
| scaffold694_59453 | JN901285 | F:GGAACAGCTGAGGTCTTTGTGTCT  R:ATATGCCTTGGCGAATTAGTCATC | 57.5 |
| scaffold694_59459 | JN901286 | F:ATGCTTCAATGCTTCCACACTGTA  R:CTAAATGACAGGAATTCACCCGAG | 57.5 |
| scaffold694_59485 | JN901287 | F:CAGAAGATAACGGTGCAACCC  R:AGTTTGATAGCTCTGCATTGACGA | 57.5 |
| scaffold249_28703 | JN901288 | F:CATCTTTCTTGGCAGAAACCACAC  R:GGACAGAACTGCAAGCTCTTTG | 57.5 |
| scaffold249_28678 | JN901289 | F:GGCCTGTGGTATTGTACATGTGTG  R:CAGAGAAAGAACATGCTGTCATGG | 57.5 |
| scaffold580_52916 | JN901290 | F:TGTGGCTCTTATCTGTGGAAGAC  R:CCACAGCAGATGGTTTGTACC | 57.5 |
| scaffold1548_78986 | JN901291 | F:GGAATGGAGGAGCAATCAACC  R:GTGTGATACAGTTCCAATGCTGTG | 57.5 |
| scaffold61_6403 | JN901292 | F:TCCACTGGGTTACAAGACACAG  R:CCCCCACTTTCACTTTGATGT | 57.5 |
| scaffold809_65154 | JN901293 | F:TAGAGCATCCAGAGCCACAC  R:GCAGTAGCAAAGCCTGAGTC | 57.5 |
| scaffold992_71865 | JN901294 | F:GGAAGCGACAATGAGTGAGTCT  R:ACAAAACACAGCAGGTGCATG | 57.5 |
| scaffold250_28714 | JN901295 | F:CAATGCGTGAGAGATGGCTG  R:ATGTCTTTGATGAGGCGGTTCT | 57.5 |
| scaffold498_24265 | JN901296 | F:GTGTGGTGTGGTTTGACTCTTG  R:TATGATACAAGGTCACTGCTGGC | 57.5 |
| scaffold485_47120 | JN901297 | F:GGGACTATTTCTGCCGTATGGA  R:AAAGGATCCCACATCGCTTAGAG | 57.5 |
| scaffold987_71723 | JN901298 | F:GCATTTAGGAGGATTGCCACTG  R:TGGACAAAAGCAGCAGCAC | 57.5 |
| scaffold1262_77386 | JN901299 | F:TGGAGGTCAGTGTGCTTCTC  R:CCATGAGGACACTCGCAGA | 57.5 |
| scaffold1069_73962 | JN901300 | F:TGTGATTGGTCGACGTCATATCC  R:CACGTGTTGGTGAAGGATGAG | 57.5 |
| scaffold646_3833 | JN901301 | F:TAATCAACCTCAACTGACAGGCC  R:GCATGAGCATTTAGTGGAGTGG | 57.5 |
| scaffold389_1656 | JN901302 | F:GAGAGTGTGAGGAAAATTTGCTTGT  R:TTAAGTCACTGCTGGTCCTTGTTG | 57.5 |
| scaffold569_3015 | JN901303 | F:GCGCAAAGTTGATCCAG  R:CTATGATTGGGAGCGAATGG | 57.5 |
| scaffold687_4190 | JN901304 | F:AACTACAGAGCCTCCAGTGTG  R:TGACGCTTTTGTGGACATGC | 57.5 |
| scaffold687_4181 | JN901305 | F:TCAAGCATAACCTACGATCACGC  R:TCGTTGGAATATCAGACCTCCATG | 57.5 |
| scaffold646_3835 | JN901306 | F:GGTGAACAATTTCCTTGGTGGA  R:TGGTATTTCATCCAGTCAGCTACG | 57.5 |
| scaffold680_27079 | JN901307 | F:GAAATGTTATCCTCATGCCAGCG  R:GACCGTTTCCGAGGTGTG | 57.5 |
| scaffold482_2517 | JN901308 | F:TCTCTCATCACCTTCACCACA  R:GTCCTCTAACCACAGAGGAAACGA | 57.5 |
| scaffold662_4024 | JN901309 | F:CGTACTGACCTCTCTGTGTCTTGG  R:AGGAAGTTGGCTCTATGACGG | 57.5 |
| scaffold662_4022 | JN901310 | F:CACAAAGTCCAGACCCTGC  R:ATCTGCTCTCTGTCCAACCAC | 57.5 |
| scaffold433_2068 | JN901311 | F:AAACACTAAGGAAATTGTTCTCCCA  R:TGAAAAGTGATTTTTGAGCCCTTA | 57.5 |
| scaffold900_69203 | JN901312 | F:ACCAGGAGACCTGACACC  R:AGCATCTGCTGCACAACAAC | 57.5 |
| scaffold152_243 | JN901313 | F:ACTGAATATTACGAGCTGAGCCAC  R:TGTGAGGATAGCCACTGTGAAC | 57.5 |
| scaffold545_2959 | JN901314 | F:GATCAGAGGACAAACATGGCATTG  R:CGTTAGCTCGTGTCTGTGGT | 57.5 |
| scaffold1216_76693 | JN901315 | F:AGTGCTTCCTATAGCCACTAGC  R:AGAATGGGGTTTGTCTCAGATCAC | 57.5 |
| scaffold903_69232 | JN901316 | F:AGTGGTTATCCATCATGCCTCAGT  R:GATATTCTCAGAGGTGGCAATCGT | 57.5 |
| scaffold1531_78950 | JN901317 | F:TCAGGGTACATGTTTATAAGGCCA  R:TCCTCAGAAGTCATGAGATAAGACTG | 57.5 |
| scaffold734_61267 | JN901318 | F:TCAGCATTATCAGCCGCTCA  R:TGAGTGTTTTTCAGCCCAGAGA | 57.5 |
| scaffold464_46166 | JN901319 | F:TTTCCAACGTCTCGATCCATCTAT  R:CCTGCAAATGTAAATCTCACCAGA | 57.5 |
| scaffold656_59185 | JN901320 | F:TTACTCTACACCAACCCACAGTGC  R:ATCTGTGTTTGGGTTGTCTCCAG | 57.5 |
| scaffold550_25984 | JN901321 | F:CGGAGGAACTACAGTGCATCT  R:GTTGCTTTGTCTCTGTGCGAC | 57.5 |
| scaffold388_40956 | JN901322 | F:AGCCACACATGGGGATTAACT  R:GTTCATCCGTTCACATGTCCTC | 57.5 |
| scaffold1332_78071 | JN901323 | F:CAGGGACAAGCTCATCACC  R:ACATCTCTGACACGGAAGGG | 57.5 |
| scaffold72_8838 | JN901324 | F:GGACAGTTCCAACACAGAATGG  R:TCCCTGACACCACACTCG | 57.5 |
| scaffold558_51720 | JN901325 | F:CGAGTTCAACCTCGAAACACAC  R:CTGAGTGGAGCCAGGACT | 57.5 |
| scaffold274_30813 | JN901326 | F:AAGGATGGAGCAAATGTAGACAGG  R:GCAGCATGTGTGAGGAGATTGATA | 57.5 |
| scaffold727_61030 | JN901327 | F:CTCGTCACTGCAGAAAGCCTTAG  R:CTGTTGGACCAGTAAGACGTGATG | 57.5 |
| scaffold1697_79096 | JN901328 | F:ACACACAAACATCCTCGTCAGAG  R:TAAGAGGAAACCAGGCCAGTG | 57.5 |
| scaffold262_29740 | JN901329 | F:TCTCTCCAAGTGTGGAGCATATTG  R:AAGCTGGAAATTGCCATAATGTGT | 57.5 |
| scaffold727_61047 | JN901330 | F:TGCAAATATTCACCCCTGTCTC  R:AAACAAACAGAAGCAGCGAGAATC | 57.5 |
| scaffold727_61032 | JN901331 | F:ATCAGAGTTAATGTGGAGTCCCGT  R:CGATTCATTACGACGTTAACTCCC | 57.5 |
| scaffold866_67929 | JN901332 | F:TCATTACGCAGCTTCACCGA  R:CAGCGTTCAAGTGTCCGTC | 57.5 |
| scaffold472_2464 | JN901333 | F:GTCATCAATACAACCACCTCCCAT  R:AGCTGAGACGAGAGGAGAAGGAG | 57.5 |
| scaffold472_2461 | JN901334 | F:CCTCAGTGCAAACTGAAGAC  R:GTGGTGGTGTTCTGGATGATGTAG | 57.5 |
| scaffold1443_78713 | JN901335 | F:ATCTAATCTGGATGTGAGAGCAGC  R:CTCTCACAGAAGCGCAGATG | 57.5 |
| scaffold426_43972 | JN901336 | F:TATCGTGACGAGGTTCCAGC  R:GTTGTGCTTACAGGATCCGC | 57.5 |
| scaffold719_60576 | JN901337 | F:CGTTGCTATGGAGACTGGATCA  R:AGTGTCAGACTGAGCAGAACG | 57.5 |
| scaffold426_43957 | JN901338 | F:GTTGTGATCCAGGCCAAAGAC  R:ACAAAACCCAGAGCACCCT | 57.5 |
| scaffold3_352 | JN901339 | F:TCAGCTTCAGACTGCAGGTTATTCT  R:GGTCAACAAAACAAAGCACATTGA | 57.5 |
| scaffold727_61025 | JN901340 | F:CCGTCAAATGGATTCAATGCTAAT  R:AGTGCATGTCTCTGGACTTCACAC | 57.5 |
| scaffold274_30815 | JN901341 | F:ACGCTGTACACAGCTACACACGA  R:AGCGTCAAAGAGTGGGGTTATATG | 57.5 |
| scaffold125_15817 | JN901342 | F:CTGTCCTATTGTCACGAGAGAGG  R:CGGTTTGTGATCAGGGCTTG | 57.5 |
| scaffold798_64331 | JN901343 | F:GTCGTCCCAGGTTCAGAGA  R:CTGGCAAATTTAGGAATGCTGGT | 57.5 |
| scaffold869_5083 | JN901344 | F:CAGCTCTGTCTCTGAGCTCCTTCT  R:ACATATTGAACGGACTGGACATGA | 57.5 |
| scaffold1194_76439 | JN901345 | F:CTCACCACTGGATGTCGC  R:TGTGGTTTCTGTGTCCCACT | 57.5 |
| scaffold512_49115 | JN901346 | F:GTAAAGATGGCCGACACTTCCT  R:TGAGAGGCTGGTAAACCACAC | 57.5 |
| scaffold557_2993 | JN901347 | F:CATACGTCCACCAAGGCC  R:CTGTGTGAAACGCCTGTGATG | 57.5 |
| scaffold426_2034 | JN901348 | F:AATGTCCGCTGAAGTTACGGAG  R:CTACTGATCTCTTGTCAGGCCG | 57.5 |
| scaffold639_56229 | JN901349 | F:CTCAATGTGAGAGGCTGAAAAGC  R:GAGACATCTGTGGTGGGGGT | 57.5 |
| scaffold1338_78201 | JN901350 | F:TGTGGCTTTATCCATCCAGCA  R:CCTGCAGAGGGAAACATGG | 57.5 |
| scaffold1338_78200 | JN901351 | F:CTGATGGAGGGGAAGTGTGA  R:GAGCGGCGTTTCATTCTATTGG | 57.5 |
| scaffold334_36814 | JN901352 | F:CCACAGCTGTCAGAACACATC  R:AGTACCACCTTAATCCCCCAAAC | 57.5 |
| scaffold557_2986 | JN901353 | F:ACAGCAGCTTCCGTAGATCC  R:CACAGCTTGTCCCCAAGAG | 57.5 |
| scaffold542_25413 | JN901354 | F:GGCTTGGAAAACATACTCAGGC  R:ACTCAGGACTGAAGGTGGAGTG | 57.5 |
| scaffold111_12997 | JN901355 | F:TCATCACGTATCAGATAGTTTGGCG  R:GGACGTCTAAAAAGTGCATGTGTG | 57.5 |
| scaffold483_24211 | JN901356 | F:CCTGTTGGTTCTTTTAAGCTGGG  R:ATGTGGAATTGACTGGCTGCTA | 57.5 |
| scaffold769_4767 | JN901357 | F:GTGGAAATGACATCCTCGCATG  R:CAATCTGATTTGGAGGCAGTGG | 57.5 |
| scaffold811_27602 | JN901358 | F:CAAATCTCTGTCACCCTGTTGC  R:CAGCGTCACTGAACTTGACC | 57.5 |
| scaffold811_27593 | JN901359 | F:TGGTTCTTCTCCAGAATCTGCG  R:GTACACGCCTACATCCATCTGAG | 57.5 |
| scaffold811_27591 | JN901360 | F:CCCTAATGGTTCTGGATGAGCT  R:ATTGATCTCTCTCAGCCACCG | 57.5 |
| scaffold572_56628 | JN901361 | F:CATCCATGAATGAGCTTTCAGA  R:CACCAATTTGAATGAGCTCTCCC | 57.5 |
| scaffold602_26430 | JN901362 | F:GCACTTATCTGTGCTTGGCTG  R:GACCTTTGCGAGATGTTGAGC | 57.5 |
| scaffold574_3077 | JN901363 | F:CATGAGAAAGTGGCTGGCATC  R:CATGCTACATTAGCACGCTGC | 57.5 |
| scaffold874_5143 | JN901364 | F:TACACTGGAGAGAATTGGCAGG  R:GCCCTTGTGTTGCTGTAGTC | 57.5 |
| scaffold568_52341 | JN901365 | F:AGATAGTGTGAGCATGTCCATCG  R:ATGTCAGACCCTCATACACACG | 57.5 |
| scaffold774_63384 | JN901366 | F:GTGACCCTGAGTAGGATAAGCG  R:GTGTTTACGTGACAAGCACCAC | 57.5 |
| scaffold434_21744 | JN901367 | F:GTATCTACCTCACAGCTTCTCCG  R:GAGATCTCAGCTGTCAGAGTGAC | 57.5 |
| scaffold402_42030 | JN901368 | F:GTCAATTAGTGGGGACGACGA  R:TGACCTATACAGCCAGCCAC | 57.5 |
| scaffold56_6340 | JN901369 | F:TCTGTGCTTGCTGCATTTTTATGT  R:TACTCATGCAGTCACAACATGCC | 57.5 |
| scaffold534_50348 | JN901370 | F:TGAGCCAGAAGACTCGTAGACT  R:GCTGCAGTAATGAACCCTCG | 57.5 |
| scaffold47_828 | JN901371 | F:GTTCACACACAGCAATACGTGG  R:GCAAGCCTCCCAGAAATAATGTC | 57.5 |
| scaffold47_834 | JN901372 | F:CCTGAGAGTAGCTGGAGACAC  R:CATCAGTGCATATGAGAGGGCT | 57.5 |
| scaffold22_2742 | JN901373 | F:CAACAAATGCCAAGGCGACT  R:CAATAGCGGAGGCAGAGGA | 57.5 |
| scaffold177_7589 | JN901374 | F:CGTCTGCATGTCTGCATCG  R:ACCATATGAGCTGGGCTCTC | 57.5 |
| scaffold1088_74361 | JN901375 | F:GCTGCAGGAGCCTATGAAG  R:GCAGAGTAAGCAGAAAGCTGG | 57.5 |
| scaffold362_18421 | JN901376 | F:GGAACCCAAAGGGACGTCTA  R:TGCTGTGTTCTTCCTACCAGG | 57.5 |
| scaffold111_12434 | JN901377 | F:GAGAAATTAAACCTGTCCTGTGCC  R:GGAAGCCACACAAACACAC | 57.5 |
| scaffold111_3880 | JN901378 | F:TGAGTGCACAAGAGATCCTTGG  R:AGCTAGTCAGCAGGCAGATG | 57.5 |
| scaffold444_2127 | JN901379 | F:TGAACTTTACAACCTCTGCACCAC  R:CACTGTGTTTGTTAATGGCGTTGT | 57.5 |
| scaffold444_2123 | JN901380 | F:AACCGATTTCATCCACAGTCAGAT  R:GTGATACAAGTAAACACCGGCTCC | 57.5 |
| scaffold518_49305 | JN901381 | F:AGTCTGCGTCTTCATGTCAGAG  R:TGGTCAAAGTCTCCACTCAGC | 57.5 |
| scaffold402_42035 | JN901382 | F:AGCAGAGAGGCAATTTCACCA  R:GGAGCCGTCCAGTGTTTATCT | 57.5 |
| scaffold547_2979 | JN901383 | F:CTTTCACACGTTTCTGGCTGG  R:AGTGGTCCGAACAGAGAGC | 57.5 |
| scaffold106_12660 | JN901384 | F:AAATCTGTCCGAGCGAGTCC  R:GGCGCAGTTAGTGAATAGCG | 57.5 |
| scaffold1094_74483 | JN901385 | F:CTGCAGGATGACAGTTGTTGG  R:CACCTGAAATGCAGAAACACGTC | 57.5 |
| scaffold561_3049 | JN901386 | F:TGCTCATTACCAGCCTCTACG  R:GATCAGGCCTCCTATCAGGG | 57.5 |
| scaffold474_23585 | JN901387 | F:CGACACATTGACTTTGAGTTGCC  R:CCAGCCTACACTGAATGGTGAA | 57.5 |
| scaffold34_4084 | JN901388 | F:TGCAGTGCTGGCATTTCTG  R:CAAACAGTCGCAATCATGGCT | 57.5 |
| scaffold243_27804 | JN901389 | F:CCCTCAATTGGCATAGTGGTG  R:GGGACATTCTTCAAACCCACG | 57.5 |
| scaffold1249_77184 | JN901390 | F:GCTAGAGGAGATGGTGTGAGC  R:ATATGTTTACCTTCAGGGCAGGTG | 57.5 |
| scaffold284_32111 | JN901391 | F:GATGGGTCTGGACAGGAAATTG  R:CACAATTTTTACATGAGGACAGCG | 57.5 |
| scaffold134_16598 | JN901392 | F:TCTGGTAGAGTAGCAGCAAAGTG  R:CATTCACGGGATCTTGGGTTG | 57.5 |
| scaffold1249_77192 | JN901393 | F:TCAAAGTCGGTTTTATAGCCAAGC  R:TCATAAGAAATCGGGACAATTTGG | 57.5 |
| scaffold145_17470 | JN901394 | F:CTTTGAGATGCGTTGACAGGACT  R:TGGAAGGGTGAGATTAAGGAG | 57.5 |
| scaffold322_35342 | JN901395 | F:CTGTTCCCAGTCTGCAGTTTG  R:TGATGGACATGTGTGTGTCAGT | 57.5 |
| scaffold1249_77187 | JN901396 | F:ATTTCTTTCATCACAGAAGCCCTG  R:AACCCACATCCTTCTTCACTCTTG | 57.5 |
| scaffold815_65508 | JN901397 | F:GCAAAGGTTTCTTAACTGAGTCTGGA  R:GCAGGATTTTTAAGGAATCAACTGG | 57.5 |
| scaffold815_65481 | JN901398 | F:ATCAGAGGGAGATGGAAAGGGTAG  R:TGCCTCTGAAATCCCAAATC | 57.5 |
| scaffold815_65496 | JN901399 | F:GTGGGCCTGCTCTATTTATACCCT  R:TCATTTCAGTCATTGCAGAGAAGC | 57.5 |
| scaffold418_43453 | JN901400 | F:TGGGGCATTAACATTAAAGCTGC  R:CTAGGCTTCAACTGCACAACC | 57.5 |
| scaffold1097_74602 | JN901401 | F:GGTGTGTGTTTGTCAGTCTCG  R:GCGTAAAGGATGTGGATGCAG | 57.5 |
| scaffold586_3215 | JN901402 | F:TGTAGTAACCTTTGTTCAGAGGAGC  R:TCTCCCAGTGTACGCAACAG | 57.5 |
| scaffold871_68129 | JN901403 | F:GAGTGTGTGTACACTTGCACTTG  R:GTGCTCATCACAGCCACTG | 57.5 |
| scaffold421_43656 | JN901404 | F:TGGTGCAAAACACACCCAC  R:GCTTCAGCTGAGCAATCTCC | 57.5 |
| scaffold138_16872 | JN901405 | F:AGGTACGTGAAGCGCTGA  R:CATCTGGACCACAGAGTGC | 57.5 |
| scaffold138_16878 | JN901406 | F:ACACAGTGCACATTTCACACATG  R:CGGGTCGATGCTCACATC | 57.5 |
| scaffold138_16871 | JN901407 | F:CAGAGAAGAGCCACGGCTA  R:GTGCTTGTGTGTACCCCTG | 57.5 |
| scaffold152_17971 | JN901408 | F:TCATGTAGGGGGAGTAACAGCTTC  R:ATTTGACAAAAGTCCTTCTGGGCT | 57.5 |
| scaffold152_17972 | JN901409 | F:GGACTTTTGTCAAATCGTCACTTCT  R:TCTTACCTCCGCTCCTGAGACTTA | 57.5 |
| scaffold1038_73254 | JN901410 | F:GAGGGCAACTATGCCACAAC  R:GAATCAGACCTCAGCCCATACA | 57.5 |
| scaffold247_28440 | JN901411 | F:CAAGTGCCAAGCTCTATGCTG  R:AGGGCTGACATCTCTGTGTG | 57.5 |
| scaffold227_28657 | JN901412 | F:CTGCTGTAGCGTAGAGTCTGAGCA  R:ACCGTAATAACACGTATACAGCACAGTC | 57.5 |
| scaffold480_2615 | JN901413 | F:TCTTCATCCTCCGATGCGATG  R:TGACTCGAGAAATCAGGGCAC | 57.5 |
| scaffold150_23522 | JN901414 | F:TTTGTGTTAGTGGTGACATTATTGCAT  R:ACACTTTGTTTGCTCTGCTGTGAC | 57.5 |
| scaffold64_7105 | JN901415 | F:AAGTAGCTCATGGACACCGT  R:CCTCTTGAACTGAGCTGCC | 57.5 |
| scaffold490_53932 | JN901416 | F:TCATCATGTCCGAGATCTGTAACG  R:TTTATCTGTTGTTCCCAAGTGGCT | 57.5 |
| scaffold599_54404 | JN901417 | F:GACAGGAAAACAACGTCAAGCTG  R:AAGGTCACGTTTACGGCTGT | 57.5 |
| scaffold779_63661 | JN901418 | F:ACACTGTTGACTCCTCGACGTACA  R:AGGGAAACAACGACACCTTCACT | 57.5 |
| scaffold969_5370 | JN901419 | F:AGTGGATAAGTCAAGGCATAGGG  R:GCACGACTTTCACCTGAGAC | 57.5 |
| scaffold779_63660 | JN901420 | F:TGTGTGTATGCGGTACTGTGTGC  R:CTGAATAAATGCGTCAGCGTCAT | 57.5 |
| scaffold410_1885 | JN901421 | F:GTAACAGCGACGTAATGGAGC  R:TTTACTTCGATCACACGACATTG | 57.5 |
| scaffold152_17980 | JN901422 | F:GGTGTGTCGCTTGACCTAGC  R:CAGCACTGTCACACTCGTACACAG | 57.5 |
| scaffold292_35193 | JN901423 | F:CAGTGGACTGGTTCTCCGCTAT  R:CCTGCTCGTTCTGTTGTGTCTC | 57.5 |
| scaffold926_5280 | JN901424 | F:CATTAGGCATCGTGCTGTCC  R:GGGCTTTGTACAACAGTCTCG | 57.5 |
| scaffold926_5277 | JN901425 | F:TGAGTTATCTGTCGTCACAGCC  R:GCGTTCAGTCACGATGTCAC | 57.5 |
| scaffold622_3760 | JN901426 | F:AGTGCTGAGGGTTGACTCTG  R:TCACGGAACAGGGACAGTTAC | 57.5 |
| scaffold926_5285 | JN901427 | F:TGGAGACCATAAGAGGCGTC  R:GAACCTCAAAGCGACCTGTC | 57.5 |
| scaffold354_1397 | JN901428 | F:GTAAGTTCACTGTGTGTTGGTGC  R:CATGACTTCCCTTCAAAGCAGG | 57.5 |
| scaffold307_34397 | JN901429 | F:AGGTCATCAGGGGTCAACATG  R:TAAAGAGGCCCAGTGTCAGAAC | 57.5 |
| scaffold1123_75040 | JN901430 | F:ACAAGACTGGCTCTCACACAG  R:GCGAGGAAATCCAACGAGC | 57.5 |
| scaffold275_31357 | JN901431 | F:ACACACATCAAGAGTCAAACCGTC  R: TGCATCATTACCATCAAAACTTC | 57.5 |
| scaffold314_34890 | JN901432 | F:GAAAGCATCAGCAGAGCCG  R:CCTGACAAGTTCGCCAGTG | 57.5 |
| scaffold500_2689 | JN901433 | F:AGCGATGGGCTTAGAGTCAC  R:ACTGAACTTGCTGACTGAGAGC | 57.5 |
| scaffold770_4690 | JN901434 | F:CTGCTCAGATGGCAACCTC  R:CTGCTTACTTTGCACGAACACAG | 57.5 |
| scaffold691_59119 | JN901435 | F:ACGTCTGAGGACTGAAGCAC  R:AGGGTTAGTGTTGTGATGGAGC | 57.5 |
| scaffold500_2695 | JN901436 | F:CCCCATGTTAATACCAGCAGC  R:GGGTTAGGGTTAGGTGTGCA | 57.5 |
| scaffold525_49690 | JN901437 | F:TTCACTGAATTTTGACTGGT  R:GCAGCAACAGAAACATTG | 51 |
| scaffold525_49696 | JN901438 | F:CGTCCAACATGAAAACATTTCACA  R:AACTGACAAGATCCATCCAACCTG | 57.5 |
| scaffold152_17986 | JN901439 | F:GCTTCAGCTCTTTCTGCACTGAC  R:GCTGGACATAAGGACAGTAATCGC | 57.5 |
| scaffold1774_79125 | JN901440 | F:GTCTGTCCTCATGCTCTTCAGT  R:AGGCACAGACACTTTCAGGT | 57.5 |
| scaffold165_22225 | JN901441 | F:TGATCAGCAAAATACTGTTCCCTG  R:TAGAGTACCTCTGACAGGCGCAG | 57.5 |
| scaffold442_21904 | JN901442 | F:CAACATTCCCTGTAGCAGCAG  R:GTCATCGACAGCAGTGATCTGT | 57.5 |
| scaffold991_71823 | JN901443 | F:TGTAATAGGACGTGGGTGAGAGGT  R:AAAGACGAGTGCAGCTCTCAAAAT | 57.5 |
| scaffold904_5174 | JN901444 | F:AGCGTTAAGGTTTAGGGTGAGG  R:TAAGGCAACAGCGCTTCAGA | 57.5 |
| scaffold167_20097 | JN901445 | F:GTTACTGGCTGTGCACACG  R:GATGCATCTGCATGAAAGAGCTG | 57.5 |
| scaffold1000_5318 | JN901446 | F:GGTGATACTCAGCCTCAGACTC  R:GTGTGGACATGATGCAGATCC | 57.5 |
| scaffold616_26453 | JN901447 | F:AGACTTGAGGAAAGCTCACGAC  R:TGGCTTTGTCCCTCAGACTC | 57.5 |
| scaffold664_58753 | JN901448 | F:AACATGAGACAGGAGACGTTACGG  R:TGTGTTATTACTGAAGCAACTGGCA | 57.5 |
| scaffold1050_5346 | JN901449 | F:ACTGTGAAGGACACACACTCC  R:AGGGATCAAAAATATCCGCGAGAG | 57.5 |
| scaffold361_1415 | JN901450 | F:TACCCTACCACCACACAAACACAC  R:TCTTTTTAGGAACTGCCAAGAAGTG | 57.5 |
| scaffold272_13998 | JN901451 | F:TGACAGGATGTATAGCTGAAGGGG  R:CTTCACATCCTCAAAAAGTGCTGA | 57.5 |
| scaffold278_994 | JN901452 | F:CCAGATCACACCACACTACGAAAT  R:TCTGCATTAGAAATTAAAAATCCCTGA | 57.5 |
| scaffold374_1504 | JN901453 | F:ACATATCCAGTAACGGAGCGAAATC  R:CTGTCAGAAGGAAGTGGGC | 57.5 |
| scaffold452_2329 | JN901454 | F:TCAGTTCCTCAGTTACTTGGGTCT  R:GCATCACTATCACGACTGTGGT | 57.5 |
| scaffold637_26984 | JN901455 | F:GTTGGATGCATCTCTGTTGAGG  R:GGCATTTAGTTTCGATGGTGAGG | 57.5 |
| scaffold374_1503 | JN901456 | F:GATTGTTGTGTCGCCACTAGC  R:ACTGAGGATGAGCCAATTCAGG | 57.5 |
| scaffold803_4929 | JN901457 | F:ACCCAAGAGTTAGCGAGAAGG  R:TTAACACCACTGCTAGCTCCAG | 57.5 |
| scaffold736_4503 | JN901458 | F:GCAGTGGCTATGCAGTATTTTGTG  R:TTTTCTTTGCAGCTCACTGATGTT | 57.5 |
| scaffold622_55485 | JN901459 | F:TCATCGCGCTTCTACAGACAC  R:CAGACATTTTGAGGAGCCAGTG | 57.5 |
| scaffold625_26661 | JN901460 | F:TTGAAGTGCTGAATGCCAGC  R:CTCGCACGCCAAAGTTTAATAGG | 57.5 |
| scaffold381_19770 | JN901461 | F:GATCGCAGGATGTGATTGCG  R:GTCCCTTGTTCTTTTGGAATGGG | 57.5 |
| scaffold272_14025 | JN901462 | F:CCTGTGACTATTAGGTTAGGTGGC  R:TGGGTGTGTAGCTGGATGAC | 57.5 |
| scaffold492_25316 | JN901463 | F:GTTGCGACTGTAAATCCGAAACTC  R:TCTGTAAGACCTTTGCCTCTGG | 57.5 |
| scaffold447_23367 | JN901464 | F:GTGTGTGTGAAGGTGTTCAGC  R:AATTAATGTGAGGGAGGGAGTGG | 57.5 |
| scaffold492_54554 | JN901465 | F:TGAACTGTAGTGGATCAAACAGAGGA  R:GCTGCTACAGCAAGTCTGTGTGTC | 57.5 |
| scaffold637_26994 | JN901466 | F:GTGGAGCAAACATGAGCCTG  R:TCAAAGCTAGTTCCCTCCGTG | 57.5 |
| scaffold849_66937 | JN901467 | F:AGAGACGGAAACTTCATCAGGG  R:AACCCTGTCTCAGCTCCTG | 57.5 |
| scaffold616_26465 | JN901468 | F:CTCTTGAGTGTGTGTGTGCAAC  R:TTCGCTGGAAGTAGAGTGGG | 57.5 |
| scaffold625_26663 | JN901469 | F:TACCACCAATGTGGAGCTGAGATA  R:ACACTCAGTAGAGCGCAAACCTCT | 57.5 |
| scaffold293_34662 | JN901470 | F:AGACAGGTGTGCATGCATG  R:ATCTCAAAGTAACGGACACTGCTG | 57.5 |
| scaffold1339_78222 | JN901471 | F:TCTCCATGACAACGAGGCTTC  R:GCAGCTGTGACCCAACTC | 57.5 |
| scaffold796_64292 | JN901472 | F:GTCAGTGTCTGGCAACAGC  R:CGTATCGCAAAGAATCGCATCC | 57.5 |
| scaffold1339_78219 | JN901473 | F:GATTGGTGCAGAGGTAGGC  R:TGTTCACACAGGCATTTCAGG | 57.5 |
| scaffold637_26992 | JN901474 | F:GTTTTCTTGCAGTGGTTCAGACG  R:CTGTTCACACCTCTACACCTTGAG | 57.5 |
| scaffold651_3900 | JN901475 | F:CGAGCTTTGAAGAACCACAGAGAT  R:TGAAATCGTTTGCAGTACATCTGAA | 57.5 |
| scaffold362_39387 | JN901476 | F:AGAACTTTGGAGGCCAGAGAG  R:AACCAGACTGAAGGCTGAGG | 57.5 |
| scaffold509_2718 | JN901477 | F:TGTCACACATAACACTTTGTCACCA  R:CTGTGAGGATGTGAAATCAGGATG | 57.5 |
| scaffold803_4934 | JN901478 | F:ACACAAATGGAAAATTGCGTCGTC  R:CAGCAGTTTAACCCCGATTAGC | 57.5 |
| scaffold1045_73528 | JN901479 | F:TAATGTAATCTCCGCTGAAGCCAC  R:CCCAAAATTCAATCAGGGGTGTAT | 57.5 |
| scaffold228_676 | JN901480 | F:CAGAGTGATGGCAGAGATCCAT  R:CCATTTACATGGAGAAGTTTAGTGACC | 57.5 |
| scaffold560_51836 | JN901481 | F:TCGTTTGAGCAGTGTAGGCAT  R:GCTGTTCTCAGTAACACCAGGAT | 57.5 |
| scaffold338_37065 | JN901482 | F:ACAATGGTTTAAGTGCAGGCG  R:ACAGAAGTCGGATGAACAGGG | 57.5 |
| scaffold848_66921 | JN901483 | F:CTCCGGATGTCAAAGTGCTG  R:TGAGGAGGTTGTCTCCAAGG | 57.5 |
| scaffold115_13516 | JN901484 | F:CGCTTGTATTTAAACATGATATTGCC  R:TGAAGTTAGAGCATTCAAAAAGGGA | 57.5 |
| scaffold269_30349 | JN901485 | F:CTGCTGAGGGAAATGGTAGAAAGA  R:TCAACTCAAATATGAAGCCACAGC | 57.5 |
| scaffold761_62829 | JN901486 | F:AACAACAAATCCTTCAGGGGTGT  R:ACTAATAGCGCACCTGGACCC | 57.5 |
| scaffold912_69534 | JN901487 | F:GGCAGGCTAACCTAACTGAAGTGT  R:GCTCAGCTCAGATCCGATTACTTC | 57.5 |
| scaffold619_55335 | JN901488 | F:TGATGACCTGATCCTCGTGTG  R:TCACCACAGTCCACTGATGTC | 57.5 |
| scaffold965_71222 | JN901489 | F:TCTTACAACCACAGACGTCCC  R:TGGTCGTCTCAAAGACAGAACC | 57.5 |
| scaffold173_482 | JN901490 | F:CAGTTCCTAAAGAGCTGCTGC  R:GATCAGATTTCTTTCTCGTGCCG | 57.5 |
| scaffold173_480 | JN901491 | F:AGCCAAGAAGTGGACTACAGC  R:AGAGAGCGATAACCCACGAG | 57.5 |
| scaffold1095_74504 | JN901492 | F:CACACAGGTTAGGTAGATCCGTTG  R:ACAAGGAAGTGGGAGAACACAG | 57.5 |
| scaffold457_23716 | JN901493 | F:TGAGTGCAATGAACAACACAGC  R:ACAGTCCATCTGTCCAGGAAC | 57.5 |
| scaffold841_66516 | JN901494 | F:TGAAAACTGCAACTGTTCACAAGA  R:TGAAATTAACCCATTAGCCAGGAA | 57.5 |
| scaffold1089_5391 | JN901495 | F:CATCCTGCTGCAGATGAACG  R:AGACAACAGCTCGACTCTGTG | 57.5 |
| scaffold457_23736 | JN901496 | F:TCAGGATGTCAGGACGCTG  R:CATTTCTCTGGGTGGGCTG | 57.5 |
| scaffold582_53159 | JN901497 | F:CGACAGACATTGGTCCCAC  R:GGAACCAACATGAGGCGTC | 57.5 |
| scaffold158_18859 | JN901498 | F:AGCGAGACATAAAGTTTCAGACGG  R:TAGAAAGCTGATTAGGCCGAAGTG | 57.5 |
| scaffold21_2496 | JN901499 | F:TGCATACAAAGAAGATTACGTGGTGT  R:CTCAGGTCATTAACACAGGGGT | 57.5 |
| scaffold408_42926 | JN901500 | F:GGACCTTCTGTGGCTGAC  R:GCTTCACCGTGTTCACTGTTG | 57.5 |
| scaffold408_42933 | JN901501 | F:CTGGTTCTATCTCCACTGACCG  R:AAGGTGCACACATGCAAATACAC | 57.5 |
| scaffold244_12553 | JN901502 | F:ATATGAGGGCATAGACGCACAC  R:GTGATGCAGAAGGCACTAATGG | 57.5 |
| scaffold357_42337 | JN901503 | F:TATTACCGCACACATGAGGAACAC  R:ATATACGGCTGTGTTGAACTTGCC | 57.5 |
| scaffold858_67466 | JN901504 | F:TGAGAACACATCTGGGGTATGG  R:TATTTCGAGCCTCATGACCACC | 57.5 |
| scaffold33_4047 | JN901505 | F:CTTGCAGCTGAGTGAACCC  R:CAAAGTGTGGATCACCAAGCC | 57.5 |
| scaffold635_56137 | JN901506 | F:GCTTCAGAAGGAGCTGCAC  R:CGATGTGAGAAAACGAATGTCGG | 57.5 |
| scaffold635_56116 | JN901507 | F:AGCTGGACACATAGTGACCTC  R:GAAATGCAAACTGCAGGCAC | 57.5 |
| scaffold336_37020 | JN901508 | F:AGGCCTCTTAAAGTGACCAGAC  R:AGCCATGTTCAGTCTCAGTCAG | 57.5 |
| scaffold906_5409 | JN901509 | F:ACCTCCAGTGAACTCTAGTGCT  R:AGACCCACAAGCCATCGTATC | 57.5 |
| scaffold456_22788 | JN901510 | F:TACACAACAGAGCCCAGAGG  R:TCGCTGTCAATAACCCACTACC | 57.5 |
| scaffold1023_72837 | JN901511 | F:ATACCTTAGAACAGCCACACAGC  R:GAGTATGTGCATGGTTTCACGC | 57.5 |
| scaffold1465_78781 | JN901512 | F:GGAACATTGCTGCTCGTTTCA  R:ACAGGTGCTCAAATTGTTCCAC | 57.5 |
| scaffold523_49655 | JN901513 | F:GCCTTATGAACTGCACAGCATC  R:GTTTCCCAGGTTTAAGCAACCC | 57.5 |
| scaffold1304_77821 | JN901514 | F:AGGTTGACGTAATCCAGCAGT  R:TCGTCAGTTAGAACCCGAGG | 57.5 |
| scaffold636_27023 | JN901515 | F:CTTTTTCCCTGGCAGCTTGAG  R:TTGCAACGACCACGGTAAGA | 57.5 |
| scaffold350_38170 | JN901516 | F:ACACACGCAAGAGGGACT  R:TGCAAGACTCACCTCAAGCA | 57.5 |
| scaffold21_2927 | JN901517 | F:AGCGGATACCTATCAGTTTGGTCTT  R:TCACATTTTTAGCTGATAGCTGAATCC | 57.5 |
| scaffold797_4865 | JN901518 | F:CCAGCTGTGAGTGAACAAGC  R:ACTTCACCCTCCATGACGTATG | 57.5 |
| scaffold469_23105 | JN901519 | F:ATTGGTTCCAGTCCCTCACAG  R:AACCCATCCCAACCCTAAGTAC | 57.5 |
| scaffold956_70993 | JN901520 | F:AGGTGCTCTGAGATGTGTGTG  R:CCACTTGTTTCCTGTTGGGATG | 57.5 |
| scaffold956_71004 | JN901521 | F:CATGGGACAGAACAGGTGTG  R:AGTGACTCTGAGCCCAACTG | 57.5 |
| scaffold220_25817 | JN901522 | F:CTGTGAACACACTCACCCCTGTA  R:TGTGTGTATGTGTCTGTGGGTTTG | 57.5 |
| scaffold226_26507 | JN901523 | F:AGCACAGCCAAGCATGAGAAA  R:GAACAAAGAGCGTCTCACAGACAA | 57.5 |
| scaffold1134_75326 | JN901524 | F:TCTTCAGTAGATGTGGCATACACG  R:GTGCCTATATGTGGCAAGAAGC | 57.5 |
| scaffold1160_75862 | JN901525 | F:GCAAGATTTAATTTCCCGGCCT  R:TTGCAGCGAGGTCTGATGA | 57.5 |
| scaffold710_4311 | JN901526 | F:TAGTCCAGAGCCCTGCATC  R:CAATCTTTCCGGGCAGAACG | 57.5 |
| scaffold216_656 | JN901527 | F:CTCCACCATCTCTGATCCCTCTTA  R:ATGTTTGTGGTGATGGTCTCCTCT | 57.5 |
| scaffold721_4477 | JN901528 | F:CTCTTGTCCAACGACCACAGATT  R:AGAGGAAGAAAAAGCCCAAAGAGA | 57.5 |
| scaffold721_4480 | JN901529 | F:TGATGGTTGTTTGTTTGTTTCTGG  R:AGATTTTGAGAGTTGGATGGATGG | 57.5 |
| scaffold710_4316 | JN901530 | F:TCTCAAAGACTCCGATGGCC  R:AAGGATGGACCGACTCCAC | 57.5 |
| scaffold540_2950 | JN901531 | F:GATAATTCGTGTGACAACACGTCG  R:CGTCACGGAGCTGCTCACTAGTAT | 57.5 |
| scaffold540_2954 | JN901532 | F:TCAGCCCTTTACCTGTTCATATCC  R:GGTTGAGTTTCTACCTGAGGACCA | 57.5 |
| scaffold744_4559 | JN901533 | F:ATGCACTTAAGGTTTGGCAGC  R:ACGTGTTATATCTCAAAACTCCGGC | 57.5 |
| scaffold493_47487 | JN901534 | F:ACTGAACCAGGATTAAAGTACAGCT  R:ACGACCCAGAGACACAACA | 57.5 |
| scaffold1348_78233 | JN901535 | F:GTCCTCAGATGATCGGGCA  R:TGAGGACACGAGTCATGGAAC | 57.5 |
| scaffold673_4142 | JN901536 | F:GTTCAGCCAATCACGCTTCTG  R:ACTTTCACAGGGAGATGGTGG | 57.5 |
| scaffold103_12047 | JN901537 | F:ACAGCTGTCTCTGGAAAACCCAG  R:TATGAAACTTGTAGCCGAGCCTGT | 57.5 |
| scaffold678_58232 | JN901538 | F:GTAACAACCTTGGAGCATGTCTGA  R:ATAGAATATCGATCGGTTTGCCTC | 57.5 |
| scaffold103_12085 | JN901539 | F:CTTTCAGATGTTGCTCTACGCAAA  R:TCGTGTGTCAACCCTCCTCTG | 57.5 |
| scaffold1022_5332 | JN901540 | F:CCTGTTCGGACTAACAGCAGA  R:CGGCATCGTCAACACCATC | 57.5 |
| scaffold1179_76240 | JN901541 | F:CGTCAGCATATCAGACCGTGT  R:ATATGACGAAGACGAGAGCGC | 57.5 |
| scaffold1727_79118 | JN901542 | F:TCAGCCATTATCAAAGACAGGGTT  R:TGACACGTCGTCTTTACACACCTT | 57.5 |
| scaffold467_46349 | JN901543 | F:CATGCGTCTCTCCTTTACGC  R:ACCATTCGACAACTGAACGTGA | 57.5 |
| scaffold386_1705 | JN901544 | F:CGAAAACATCTGAGTGCGAGC  R:TGTCTGTTTGACCTCGACTGTG | 57.5 |
| scaffold386_1706 | JN901545 | F:CAGAGAAAGGTGACCGTCGAT  R:CCTGAGACACAAACACAAGGGTA | 57.5 |
| scaffold30_3929 | JN901546 | F:ACAGACTTTAATCAACAGCGAGGC  R:AAGATTGTTTTTCCACCTCTTGGC | 57.5 |
| scaffold60_6618 | JN901547 | F:GGACTGTAAAATGGGAACTCATGG  R:GCTGATCACTCACAACCGC | 57.5 |
| scaffold200_10638 | JN901548 | F:TGTCCTCCTCAAAGCCTGAG  R:TGACGAGGATGACGTGTCG | 57.5 |
| scaffold297_33298 | JN901549 | F:ACAGAGATGACGTCCAGGTC  R:CTTGTGAAACACTGGACGGAG | 57.5 |
| scaffold200_10602 | JN901550 | F:CCTCTGTAGACCCGACCATC  R:TCCTGAACCTCAGGTAACACAC | 57.5 |
| scaffold297_33307 | JN901551 | F:GGAATCTCCTCAGTGTGGGA  R:AGCTCGGTGGTTCACTTTCAT | 57.5 |
| scaffold122_4728 | JN901552 | F:CCAGTAGTGTGTATGTGTGTGCT  R:TGAGGTAAAAGTCGTTGGCAGAG | 57.5 |
| scaffold557_51574 | JN901553 | F:TGGACAGGAGGCTAATGTGG  R:GCCTGAGTGCAAGTCAATCC | 57.5 |
| scaffold792_64200 | JN901554 | F:CAGGCCTCAGACGTGAGT  R:CAACTTTTCAGCAGTTGGGCA | 57.5 |
| scaffold450_2404 | JN901555 | F:ACTCCGGACTGGAAGAGACAGAC  R:GCAAATGTCCCTGCTGTTTACTCT | 57.5 |
| scaffold722_4488 | JN901556 | F:ATGTTTGTGTTGGGCTCTGC  R:CATGTTGTGAATTTGGAGCCAGTC | 57.5 |
| scaffold810_5030 | JN901557 | F:ACGACCTGGTTCAGGTAACAC  R:TCAGGAAGTGGAGACAGCAAC | 57.5 |
| scaffold450_2401 | JN901558 | F:ATGCAGAGCTTCTCTCTGGTG  R:CCCTGACATGTTGTGAGGAGT | 57.5 |
| scaffold722_4485 | JN901559 | F:ATGTTGCGTGTTGCAGACG  R:TCAGGTTGATTCCCCACAGTC | 57.5 |
| scaffold498_48139 | JN901560 | F:GTCCTGCAAAGTCAGGTGG  R:GGATGGAGGAAGAGGAGTCAC | 57.5 |
| scaffold692_4207 | JN901561 | F:ATCATACACGATGACCAGGCC  R:CCTGTTGACAGGCTACATGC | 57.5 |
| scaffold277_31098 | JN901562 | F:GCTCTCACTCCGAGCAAATG  R:CGTGACGTGTTTCAGAGTCC | 57.5 |
| scaffold497_48029 | JN901563 | F:GCTACAGTATAAGCACAAATGCGG  R:AGCTGAAATGTCATCGCTTAT | 57.5 |
| scaffold185_22112 | JN901564 | F:TTGTTTTTAGTTCACAATGATGGGC  R:CAGAGCTTTGCACCACATGATAGA | 57.5 |
| scaffold583_3134 | JN901565 | F:GAACCTGCTGTTTTGTGAAGGATT  R:TGTGATGGCTACACAACTGTTCCT | 57.5 |
| scaffold823_65878 | JN901566 | F:GGGCCTTTACGATCATCTGTG  R:CCCATTGTATGCTGGGAAGTC | 57.5 |
| scaffold338_1352 | JN901567 | F:GTGTTTCAGCGAGACGAGC  R:GATGACGCCACAATGTCAACAC | 57.5 |
| scaffold77_144 | JN901568 | F:CCTGCTCAAGAGGAAACCTCAG  R:CACTGAGGACACATACTGTACCGC | 57.5 |
| scaffold11_1122 | JN901569 | F:ATATGACACTGTGCCTGCCT  R:TGTGATCTACCTTGGCCTCC | 57.5 |
| scaffold804_64865 | JN901570 | F:GCCCTATAGGCTGAATCCTGATCT  R:TCAGGTCAACTACAAGATGGTTGC | 57.5 |
| scaffold391_41147 | JN901571 | F:CTCTCAGCACTTCCTACTGTCATGT  R:CTGATTGGCTACTAACGTCAAACG | 57.5 |
| scaffold771_60639 | JN901572 | F:CCATAACAAATCCTCCTCCAGATG  R:TTACGCTAAACGGCTTTTCTCTGA | 57.5 |
| scaffold563_56175 | JN901573 | F:AAGTAGAAGAAACATAAACATGCGGA  R:TGAGCCTTTCGACTCATTTAGACA | 57.5 |
| scaffold563_26075 | JN901574 | F:TTCTGGACCAGTCGAACTGTC  R:AGAGCCTTAACTGAGCGATTTCAG | 57.5 |
| scaffold563_26099 | JN901575 | F:CTTCAATTGCCCATGGGACAG  R:GTTCTGTAGGGAAGACGCTCTC | 57.5 |
| scaffold1151_75592 | JN901576 | F:CAGGTAACTCGAAAGTGAGTGCA  R:TGATGAACAGAGCAGGAGGG | 57.5 |
| scaffold615_3473 | JN901577 | F:AGTCTAAGGTTATGGGGCAGAGT  R:CAACCGGTGAAGTGATGATTGG | 57.5 |
| scaffold338_1351 | JN901578 | F:GTTTGTGTGCTGGACCTCG  R:AATGTGGTTTGACCGGGTTTG | 57.5 |
| scaffold338_1350 | JN901579 | F:TTGCCATCATAGATCGAGTGTCC  R:CAGCCAAAGGTAAGAGCAAGC | 57.5 |
| scaffold762_4683 | JN901580 | F:AAGGTTCTAAGGTCTGATGAGCC  R:GTTTGTTTGGCTGCATGTGG | 57.5 |
| scaffold643_3816 | JN901581 | F:ATATTCGGTCAGGTCAGGACG  R:GATGATTTCTGCGTTCCCTGG | 57.5 |
| scaffold762_4679 | JN901582 | F:ACACTGATCTCTGACCTGTGG  R:CTGTGTGTCAGCAGCTCATG | 57.5 |
| scaffold643_3815 | JN901583 | F:CATTAGTCCAGGGTGCGTTG  R:CTCTGATGGTTTGGCAAATGTCG | 57.5 |
| scaffold178_428 | JN901584 | F:GGACATGTGTCGTCAAGCC  R:GACTAACAGGGTTACCTCCAGG | 57.5 |
| scaffold1334_78084 | JN901585 | F:CTGGTGAAAACAGGGAGCG  R:GTCAGCGGGGTCAGTGTA | 57.5 |
| scaffold186_22422 | JN901586 | F:GATGCTTTTCCTAATGGGGTGAG  R:GATTGAAGCTGAGGGGGTG | 57.5 |
| scaffold762_4682 | JN901587 | F:ACATGTACCCATTAGCAGAGAGG  R:GCTCCAATCAAGTGTAGCAGC | 57.5 |
| scaffold77_9702 | JN901588 | F:CTTCAATGTGCGGCTCTCAAAG  R:AATTCAGTAGCACATGTTTTGGCC | 57.5 |
| scaffold699_4200 | JN901589 | F:TAACATCCATCCACGACTTTCTGA  R:GTGTTCCAACATGAGGACACTCTC | 57.5 |
| scaffold767_63016 | JN901590 | F:ACCTGCTGACTCCAAGAGAG  R:GGCACCACAATGCTGG | 57.5 |
| scaffold1615_79059 | JN901591 | F:CTTCCGAAGTTTGGTCCAGG  R:ATTTCGCACACAGGATGGG | 57.5 |
| scaffold1163_75931 | JN901592 | F:CCTTGACAGAGGTTCGGACT  R:CAGAGATGGACGGAGACATACC | 57.5 |
| scaffold320_38827 | JN901593 | F:ACTTTTCTGGGACAGCGTGCTT  R:CCCGACCTTTGGAATGTC | 57.5 |
| scaffold998_72045 | JN901594 | F:CCAGCATAAGTAGGCCCTGA  R:CTGCTGTCTCCGTATGAGGTAAAG | 57.5 |
| scaffold704_59923 | JN901595 | F:TCAGGTCTGTGAAGTCAGGATTG  R:CTCGGTTGCCACGTAAATCAC | 57.5 |
| scaffold704_59895 | JN901596 | F:GACACTATTGCGGGTCACCATA  R:CACAGGAACAATCCATGGGC | 57.5 |
| scaffold299_33764 | JN901597 | F:AGTCCATTTCCTGGTGTAAAGGGT  R:TGAACTGCCTAATCAAGAGGAAGG | 57.5 |
| scaffold791_64165 | JN901598 | F:CAGCACATATGAGGAGGAAAGG  R:GCATTGACAGAGAAAACAGC | 57.5 |
| scaffold356_40874 | JN901599 | F:TGTAGCTGCAGCCAGTTATAC  R:GGCTCCCTATTTGTTCTCTTGTCA | 57.5 |
| scaffold114_6863 | JN901600 | F:CACTGTTTCATCAACAGACATTGGA  R:GCAGCTCTGCCAGTATTTTTGACT | 57.5 |
| scaffold114_7180 | JN901601 | F:GGTTCTTTCGACCAAGGGC  R:GAACCTGTCTATCACCGCAAACT | 57.5 |
| scaffold224_11538 | JN901602 | F:CTTATTGACCTGACAGGGTGGT  R:GCAATGCTGTTTAGCCCTGAG | 57.5 |
| scaffold252_13429 | JN901603 | F:ACATGACGGGAGTGGAAGG  R:GACCCAGAAGTCAACAAAGGC | 57.5 |
| scaffold487_47127 | JN901604 | F:CATTTGGCCTCCTCCTGAAC  R:TGTGCTCATTCTGCATGCTTTC | 57.5 |
| scaffold291_14055 | JN901605 | F:TCACGCAATGTACAGGGTGT  R:CCACTTGATTTATAGCCTGCGC | 57.5 |
| scaffold291_14047 | JN901606 | F:GGTAAGAGCCATTGTGTGAGGA  R:GGAGAGGACAGGGTGAGTC | 57.5 |
| scaffold349_38041 | JN901607 | F:GATGAAAGGACTTAGTGAGACGGG  R:GTTGTGGGACGAACACAGTC | 57.5 |
| scaffold349_38068 | JN901608 | F:CCATTGTGTTAAGCCATGTAGCC  R:TGACATTAGTAACCAGGCAGACC | 57.5 |
| scaffold291_14075 | JN901609 | F:CTGCTGAGAACTCGTCTCTAGG  R:ATTTCTTCTCCTCACCGCATCTG | 57.5 |
| scaffold658_57211 | JN901610 | F:CCTGCTTGCTTTCTCACTCC  R:CCGCTTGGCAGACAAAAGT | 57.5 |
| scaffold281_31690 | JN901611 | F:GGTAGACACATACACAGTGCCA  R:TGGCAACTGGAGAGAGGAC | 57.5 |
| scaffold647_56668 | JN901612 | F:CTTTTGTTACATGGACTGGAGCC  R:TCCAGGTCATTGGACGACAG | 57.5 |
| scaffold28_458 | JN901613 | F:CTGTGTCCTGACTCAGACCAG  R:CTTTACTGCAGCCAGCCAC | 57.5 |
| scaffold92_10931 | JN901614 | F:CTGTGTTGTCAAATGTGCCGT  R:GAATGCTCTTGACTTGCGTGG | 57.5 |
| scaffold277_13195 | JN901615 | F:CAGGTGATTCACGCAGAACTTG  R:CCGGCTGCTATGACGAC | 57.5 |
| scaffold257_29444 | JN901616 | F:TGAGACCACACTGTCTGTTGG  R:GCACAATGGAGCGTTGAGTC | 57.5 |
| scaffold257_29469 | JN901617 | F:GTATCGACATCTGCCTCCGT  R:ATCCATTCAGGTGCAGTAGCA | 57.5 |
| scaffold86_10449 | JN901618 | F:ACGCTGAGGTGCTGTATAGG  R:TTTGGCAGTGTGGTTGTGG | 57.5 |
| scaffold1170_76103 | JN901619 | F:CGTCGAGGTGGAAATCTGC  R:TGAGGCCTCGTGCTGAATAAT | 57.5 |
| scaffold257_30957 | JN901620 | F:TAAACGTCACAGAGACAGAGCAGC  R:ACAGGATCACACCAGTCACAG | 57.5 |
| scaffold739_4710 | JN901621 | F:GATGAAAGTGAAGCTGGGCG  R:ACAACCTGATGTTTGCACGTC | 57.5 |
| scaffold749_61883 | JN901622 | F:CACTTGAGAGCAAGCTGTTGG  R:TCGGGAAATTAACCCAAGTC | 57.5 |
| scaffold526_49786 | JN901623 | F:TGGTGGCAACTCTTCTGGT  R:GGTACTGTTTGTCGGCCC | 57.5 |
| scaffold581_3651 | JN901624 | F:CTTTTGTTTTATTCTCCTTCGCCC  R:TGAAAATGAGTGTTTGAAGGGTCC | 57.5 |
| scaffold246_28153 | JN901625 | F:TCAGTGACAAACCGTTGACGT  R:GTCGTGTTTATATCAGGGGTGAGT | 57.5 |
| scaffold671_4090 | JN901626 | F:CGATGACATGCTAGTTTTCAGGC  R:TGACCACAAACTGATGTGAAAGTG | 57.5 |
| scaffold570_3141 | JN901627 | F:AGAAGGTCGTCAGTTCAATCCC  R:TGCTGGACAGATGTGAGACAG | 57.5 |
| scaffold888_5302 | JN901628 | F:GACGAGTGAAGCTGTCACCT  R:ACTCAGCGTCAGAGAGCTG | 57.5 |
| scaffold1167_75960 | JN901629 | F:GTGAGATGGTTGAACGCACTG  R:TGAACTTCTGCGTTCTCTCTGG | 57.5 |
| scaffold864_67794 | JN901630 | F:CGACCACTATGGATGTCCTGAG  R:CAATTAGTGGACGACCTCCTGAG | 57.5 |
| scaffold570_3149 | JN901631 | F:TCCCTGACATCAGTGAACCAC  R:CAGGTCTTGGCACCTTGAAAC | 57.5 |
| scaffold739_4726 | JN901632 | F:TGACCAGTACCTGAGCTGAAC  R:CCATGGTCACTGCCAGAC | 57.5 |
| scaffold611_59861 | JN901633 | F:ACTGCATCACAACGTACATCCTC  R:GTCTATTCAGGACTTGAACTGAACCT | 57.5 |
| scaffold581_3652 | JN901634 | F:CCATTAACATGGCTGACGTTCC  R:GTTTACACTCACACATCACTGGGT | 57.5 |
| scaffold1874_79149 | JN901635 | F:TGTGCCATTAACGCTGTGTG  R:TCATTCACTCTGACAGGACGG | 57.5 |
| scaffold578_52877 | JN901636 | F:CTTTGTAGCCCCACCCCTTATTAC  R:TGAGAGCTTTGAGACATTGACACC | 57.5 |
| scaffold295_33190 | JN901637 | F:CAAACTTCTCAATCAGCATGACGT  R:CTGCTAAGATCGGATACCCTGT | 57.5 |
| scaffold502_48282 | JN901638 | F:TCATTACAGGCAGATTACCTTACTGC  R:CTGGACATGATGACAAACCACAG | 57.5 |
| scaffold1005_72254 | JN901639 | F:GTGGACTCGAGCTAGTCACAACAA  R:CTTAAGTGTATGTGCACACGGAGC | 57.5 |
| scaffold89_2240 | JN901640 | F:GTTGACAAAGGCGTCAGGTC  R:CGAGTCCAAGTGTAGGAGTGG | 57.5 |
| scaffold89_2229 | JN901641 | F:TCAGCTGACATACTGTGTGTGAC  R:CAAACCTCGGCTGTACGTG | 57.5 |
| scaffold237_12191 | JN901642 | F:AAACAGTCTCTAACTGCGTCCG  R:TCTGTTTTAGTCCGTCCAGCAG | 57.5 |
| scaffold160_332 | JN901643 | F:ATTTGGACCAGAAAGGGATGAGAC  R:TGATGCCGCTCACACTTTACTTTA | 57.5 |
| scaffold396_45547 | JN901644 | F:CAACAGTTGAGGGATTTTTCCTGT  R:AGCCTGTGCACACTGAATTTAAGA | 57.5 |
| scaffold1066_73956 | JN901645 | F:TGCTGCCTGTTGACTTTGTG  R:GCGATGGTAAACCTGTCTGC | 57.5 |
| scaffold396_45575 | JN901646 | F:ATAGTTGTTGGGGGTTGTGTGTCT  R:CAGCCTGACAACTATAGGCTTGAA | 57.5 |
| scaffold368_20042 | JN901647 | F:TCAGACATGATAGCAGCAGGAC  R:TTAGCTGGTGAGCCGAACTC | 57.5 |
| scaffold237_12187 | JN901648 | F:ACTGTGTGACGTGTGAATGCA  R:TCAACCGTCACATTACAGGAGG | 57.5 |
| scaffold510_48896 | JN901649 | F:CCTTAACCATGAGTGTGATGCTC  R:GTAAGTGGTCAAACACCCAGTG | 57.5 |
| scaffold1066_73955 | JN901650 | F:TCGTCAACTGGTGGTTGTTAGAG  R:GGCTGTCATCATTACGCTGC | 57.5 |
| scaffold658_59018 | JN901651 | F:CAAGCTGTCGCTGATAATGGTG  R:CACCCCTAGTCTATGTTGTTGGCT | 57.5 |
| scaffold160_333 | JN901652 | F:GCCCTGTCCTGTGTCTTACTGT  R:CTTTTTCCCTCAGCACAGAC | 57.5 |
| scaffold553_51374 | JN901653 | F:TGATGTGGCTGTTAGCGTTGA  R:TGGCCCAATACAAATCTGACGA | 57.5 |
| scaffold1183_76308 | JN901654 | F:TGGGAATTACGAAAGCCGC  R:ATGTTCGGAGACAGCTAGGC | 57.5 |
| scaffold494_47606 | JN901655 | F:CCACCATGATTCATTCCCGTC  R:GCAGCCAGAAGTCTCAGC | 57.5 |
| scaffold368_20032 | JN901656 | F:GTGTGCTGTATGGATGGCC  R:GGATCCAAATGCTGGATCTCCT | 57.5 |
| scaffold189_22532 | JN901657 | F:GAAACTGATGCTCCATGATCCC  R:AGAGACTCACACAGTCGGAGCAG | 57.5 |
| scaffold97_11382 | JN901658 | F:ACATATATGGGACACACACCCC  R:GAGGTCAAACGTTATCCACGGT | 57.5 |
| scaffold285_16871 | JN901659 | F:GGTGTTTCCCTCACTTCAATGG  R:CTGATGATAGAGCCTGCGATGT | 57.5 |
| scaffold285_16864 | JN901660 | F:ACTTGACGAGGCTTCATCTGAC  R:TCTGATGACAATAAGCAGTGGGG | 57.5 |
| scaffold138_14871 | JN901661 | F:TGGTCCCCACAACTTCAGTTATAC  R:GACCAGTATTGCCATCTGAGTACC | 57.5 |
| scaffold878_68370 | JN901662 | F:TGGAGGTGAAGCAGTCAGTC  R:ACTGACACCTGGATGTTCTGTG | 57.5 |
| scaffold215_25088 | JN901663 | F:TGTGTATTGTGTAAGCATTTGCAGC  R:TACTCAGCAAAATGCAAATGGAAG | 57.5 |
| scaffold1028_5334 | JN901664 | F:GGATTTCACAGCTGTTCCTGG  R:TCAACATGAAGATTCTCCTCAGACG | 57.5 |
| scaffold254_33077 | JN901665 | F:CAATAAAACAGGCCTCAGTAGGCA  R:TGACAGAGGGCCAGAGATATAAGG | 57.5 |
| scaffold1028_5335 | JN901666 | F:TTTGAACTCCAGGGTCTACGTC  R:GCGTGTTACGTCCAGTGTG | 57.5 |
| scaffold868_67940 | JN901667 | F:CATTCCACAGCAATTGGCAGT  R:CAACACCTGTCTCACTGCAC | 57.5 |
| scaffold681_4098 | JN901668 | F:GACTTCTTCAGTTGGTGGCAAG  R:CGATCGTAAAGCAGTGAGGGT | 57.5 |
| scaffold1058_73722 | JN901669 | F:CTCTGACAGCTTTAGCGGC  R:TGTTGTGCTCAGAGGGTAGAG | 57.5 |
| scaffold260_31838 | JN901670 | F:ATCTGGCGATAATCTTTGTTTGGT  R:GGGATGTTAACTGTATTGTTGAGTCTG | 57.5 |
| scaffold638_56171 | JN901671 | F:CCGTCTAATCTGTGCGTGAAAGA  R:CTGCTGCAAACATGAGTCTGAG | 57.5 |
| scaffold634_56015 | JN901672 | F:TGACTAGCCCCATTTCACCA  R:TGCGTGTGTGAGTCTCCAT | 57.5 |
| scaffold638_56169 | JN901673 | F:GATGACCCCTGAACTTTGTCG  R:GCAAGTGCATCACCAAACTGT | 57.5 |
| scaffold1354_78310 | JN901674 | F:CTGCTTATTTGGCCGAAGATCAC  R:TCTGGAATACTCTGCCGTCAC | 57.5 |
| scaffold213_24768 | JN901675 | F:GAGACGTCAAACACGGATGGA  R:GGGTAAACTCGGACAGATGGAG | 57.5 |
| scaffold367_18844 | JN901676 | F:GGCTTATTGAACATGTGGACGAC  R:AGAAGGTTGGGACCAGAGGCT | 57.5 |
| scaffold459_45861 | JN901677 | F:GTTCTGGGCATATGAAGCGAC  R:GAGCTGAATGGGCAGAAACC | 57.5 |
| scaffold195_10329 | JN901678 | F:GGTAAGGTTTAGAGGCTAGGGAC  R:CAGGTGAGAATGATTTGTTGGAGC | 57.5 |
| scaffold367_18839 | JN901679 | F:TTCTCTGCTCACGGCTTATGAC  R:GGATGGATGGAAATGCCAGAC | 57.5 |
| scaffold1133_75278 | JN901680 | F:AGGATGAAGAGCTGGTGGAC  R:CAATGTTGCCAGGAGCTTGT | 57.5 |
| scaffold285_16821 | JN901681 | F:CAAATCAATGAAGCGTCTGCTCC  R:GAACACGCTCAGAAATGTCCAC | 57.5 |
| scaffold1133_75280 | JN901682 | F:TTGTTGTCAGAAGCCACTCCA  R:TCCTGCAGGGTAATCGTCC | 57.5 |
| scaffold526_25118 | JN901683 | F:TGCTTCTCCTGAAAACCTCAGTC  R:ACACCAGCTCACTCTGAAGTC | 57.5 |
| scaffold1350_78279 | JN901684 | F:TCACGTTCTAAACTGCAAGACAGC  R:TAACGGAAGGCATTCACCAG | 57.5 |
| scaffold552_51132 | JN901685 | F:GGCCCCATTAAACTGCATTAAAAC  R:TGAAATACAGGGCAAGAGGAAGAG | 57.5 |
| scaffold299_36547 | JN901686 | F:AATCTACATCCACACCAAAATTG  R:TGACAGCTCGTATATCATTCC | 57.5 |
| scaffold634_56016 | JN901687 | F:AGTCCTCATCATGCTTGGTCC  R:ACAAACACCACAAAACTCACACG | 57.5 |
| scaffold58_8863 | JN901688 | F:ACTTTGCGTATTCACCCAAAACAC  R:AAGTTTGGATCCTCGCCTTTAGAC | 57.5 |
| scaffold946_70606 | JN901689 | F:ATGCTACAAATTCTGGCCCCT  R:ACTGCACCACTACCTCCAG | 57.5 |
| scaffold1004_72228 | JN901690 | F:AGATCACTTTGTTTGCACTGCTC  R:GCCTCCAAAATCTGAGACAGG | 57.5 |
| scaffold513_49143 | JN901691 | F:CCCAGAGCAGCTTAAACATAGATTC  R:CATTTCTCTTGTTCTCCCTTAGCTTT | 57.5 |
| scaffold513_49203 | JN901692 | F:TGTGTCTGGTGAGCGTTTTAACAT  R:GACATAAGGCAGAGACGGGAGTAA | 57.5 |
| scaffold1047_73552 | JN901693 | F:GTCAGACTTCGACATCGGCT  R:GCATGAAGGTTATATCGCACGAGT | 57.5 |
| scaffold814_65354 | JN901694 | F:ACACACTAAGTAGTGGGCACATC  R:TGAACCCTCTCGCTAGCAC | 57.5 |
| scaffold443_44729 | JN901695 | F:CCTTGCAAACCAAGGAGTCAG  R:TGAATAGCAAGACCCCTGCA | 57.5 |
| scaffold141_4926 | JN901696 | F:TGGTGATCCAACATCAGACACG  R:TGCTTTTCTAATGGAGCGAGAGAG | 57.5 |
| scaffold587_53479 | JN901697 | F:CACATTACTCTGCATGGCACAAC  R:CACAGTTTACTTGCCACACACAAC | 57.5 |
| scaffold78_8899 | JN901698 | F:CATGTGTGTTCACATACCTGGC  R:TCCCATAAGTGTGAGGTTGGG | 57.5 |
| scaffold78_8905 | JN901699 | F:GCATTCCACACCAGGTTCAG  R:TCAGAATGGAGACCAGCACTG | 57.5 |
| scaffold492_47464 | JN901700 | F:CCTACTATTCCTGTCCCTTTAGCC  R:GGTCCTGTTTGTTCGCTTTGAA | 57.5 |
| scaffold222_27448 | JN901701 | F:CTGACTGCTCGCGGTTAAATG  R:GTATGGGAGGAGGGAGAGATG | 57.5 |
| scaffold614_55175 | JN901702 | F:AGCACCTAAACCATCTGGGT  R:TGAACCACACGTACAGCCA | 57.5 |
| scaffold801_64588 | JN901703 | F:GAACCGATGGATGCTACGTG  R:GTGGGCCTCACTAGATGTAACT | 57.5 |
| scaffold761_62825 | JN901704 | F:GTCAGCCCTCTGCTCTGTA  R:CTGGCAGCCATCTTTTACTCAG | 57.5 |
| scaffold725_60936 | JN901705 | F:GCCTTATTGGCGGATAGAAAACT  R:TGATGTTCTCTCTATCCTGTGGTG | 56 |
| scaffold937_70411 | JN901706 | F:TCTGGCAAAATTACAGTGAATGGA  R:CCCACCTTCTTATTACTGAAACCCA | 56 |
| scaffold578_52892 | JN901707 | F:AGAAGATGTCCATCAGGCAAG  R:ATGACGTCGCACAGAAACA | 55.5 |
| scaffold420_43574 | JN901708 | F:TGCCAAATCAAGTTATCTCACTG  R:CTCTGTGCTAACCAAGATGC | 57.5 |
| scaffold113_4049 | JN901709 | F:GATCGTAGACGAGTTGCCATGA  R:GGAAATGTTATAAACCGGAAGGGGA | 57.5 |
| scaffold449_2147 | JN901710 | F:ACACAGTGCACATTTCACACATG  R:CGGGTCGATGCTCACATC | 57.5 |
| scaffold725_4612 | JN901711 | F:TCAGTGTGCTCCGCTAACA  R:CTGCCAGAGAGGGACAGAT | 57.5 |
| scaffold799_64361 | JN901712 | F:GTGAGCCAGAGTGAGTGC  R:CGACGACTTCAACGAGACCT | 57.5 |
| scaffold799_64400 | JN901713 | F:TGACGTATGTGTTGCATCTGTGT  R:CCTTTGTATTATCCGGGCGTACA | 57.5 |
| scaffold8_991 | JN901714 | F:ATCAGATGCTAGAGGCTGCTC  R:CTCACCAGCATTGTATTTGGACAC | 57.5 |
| scaffold414_43382 | JN901715 | F:ACCAGTGGTTGAAGCTGTAGAGG  R:CTTCCTCATCTAGTTGTTCCCCC | 57.5 |
| scaffold832_66192 | JN901716 | F:CTGGCTGCTAAATACCTGGACAA  R:CACGGCCTAGTAACTGGATCTG | 57.5 |
| scaffold156_235 | JN901717 | F:GGCATTGGAAACCGTGTAGTC  R:TCAGAGGAAGAAGCAGAAGCAG | 57.5 |
| scaffold310_15659 | JN901718 | F:AATCTTGACAAAGCGTGAAAAC  R:TGTGTCTGATCCCCCTGTCT | 57.5 |
| scaffold39_2929 | JN901719 | F:GTAAAAGTCTGGCAGGGGATTG  R:GCGTCACTGAACTTGACCAC | 57.5 |
| scaffold533_2930 | JN901720 | F:GGTAGAGTGAGGTACCGTGTG  R:TCACTTCCCAATTGTCAGTGAGTC | 57.5 |
| scaffold674_4051 | JN901721 | F:TGGCCACAGTGTTGCTAATTC  R:GGCTCAGTAAGGAAGGTGC | 57.5 |
| scaffold1035_73111 | JN901722 | F:CGCAGAGTTGGATCTGGTG  R:CTTCTCTTTTGCATCGTGGTCTC | 57.5 |
| scaffold20_91 | JN901723 | F:GTGATGTACTTGTGTACAGCTACGT  R:TCTGTGTTTGAACAGACGAGCA | 57.5 |
| scaffold293_32994 | JN901724 | F:GGGCTAATGAGTGGTTGCAG  R:CTTGTGTCTCCATGGTAACAGTG | 57.5 |
| scaffold293_32897 | JN901725 | F:GAGTCACCTTGTTTCAAACCCTG  R:ACATGAGGAGAGTCGCACTG | 57.5 |
| scaffold807_65101 | JN901726 | F:CCACCTACCTTCAAGCTCTGA  R:AACTCCCAACATGCTCTGTCA | 57.5 |
| scaffold807_65106 | JN901727 | F:CGACCTTACATGCATCGTTGG  R:ACACAGTTTCCCCTCCATCAG | 57.5 |
| scaffold1078_74104 | JN901728 | F:ACTCTAATGCTTTTCAACATGCCC  R:GATAACTCAGTGCAACTGGCTG | 57 |
| scaffold186_9468 | JN901729 | F:AAAGTTTGATTGGGAGGCAGC  R:CATTACTCTCCAGCCACGC | 57.5 |
| scaffold291_32671 | JN901730 | F:CCTTTATACGTGAGGGTCTGGG  R:TCTCTCAGGAACATGTCACTGC | 57.5 |
| scaffold360_39285 | JN901731 | F:CATCACATGGCCCAAGAAAC  R:TCCCTCTTGAGGAGAATTAAGGA | 57.5 |
| scaffold391_1095 | JN901732 | F:GGCAATCCAGATGTTTTGGCT  R:ACTTCACGTTGCTGAAGCG | 57.5 |
| scaffold391_1034 | JN901733 | F:CTGATCAGTGCAGAGATCCTGT  R:TCCTCATCGTAGCTGTAGGACTAG | 57.5 |
| scaffold414_21274 | JN901734 | F:CCATCCATCTCCTGTCGATCTG  R:GATAGTCCCAGACTTTCACCTCAG | 57.5 |
| scaffold414_21261 | JN901735 | F:CATGATGATAGGAACGGCCAGT  R:TGGTCACTCTTCTGGTGCAG | 57.5 |
| scaffold414_21207 | JN901736 | F:ACGCTCAATACCTTGGAGGATG  R:CTTGAGGATTACATCATTGCACAGC | 57.5 |
| scaffold41_1313 | JN901737 | F:GCCGCATCTTCTCACAAACAG  R:GAAATGTTTCCAGCAAAGGTCTGG | 57.5 |
| scaffold420_1897 | JN901738 | F:GTTATCTGTCGTAGTCAGGAGGC  R:GCCTTGGACATAAATCCACCC | 57.5 |
| scaffold13_1985 | JN901739 | F:TGGCCACAGTGTTGCTAATTC  R:GGCTCAGTAAGGAAGGTGC | 57.5 |
| scaffold1671_79088 | JN901740 | F:ACAGAACTCCACACACCTGTC  R:ACCATCTCACCTCACAACTCAG | 57.5 |
| scaffold306_16082 | JN901741 | F:CATTTCCTCTGTAAAGTGCACCG  R:TAAGTTGAGAGCTCCTGGGACT | 57.5 |
| scaffold85_8283 | JN901742 | F:AGAAGTCCTGACTGTGTGCC  R:AGTAAACAGACTTGGCGACCAG | 57.5 |
| scaffold873_5042 | JN901743 | F:GAAGAGTCACCTGTCTGGTCTG  R:CTTGACCATGTAGCCAGCTTCT | 57.5 |
| scaffold1000_72180 | JN901744 | F:TGGTGATTATTACAATAATCTCACAGC  R:GCATCCTCAAAATGAGCTGC | 57.5 |
| scaffold1108_74785 | JN901745 | F:CCATGTCCGAACAAACAAGTACAC  R:TCGGTTACACCGCATATAGCTC | 57.5 |
| scaffold136_16795 | JN901746 | F:GTTACAGCAACCCCAAGCTG  R:TCACAGCAACCCCAAGCTA | 57.5 |
| scaffold398_41601 | JN901747 | F:CACCAGCATGAAAGCAACCT  R:GTCCACAGACAGACGGACA | 57.5 |
| scaffold398_41622 | JN901748 | F:ACAGAGCATGTCTGGCATCT  R:GACTAAACCACAGAGTGGTGACA | 57.5 |
| scaffold428_44072 | JN901749 | F:TCTCTGTCTATCGGCAAGGC  R:GCTCGCACAAGTTTATAGAGCC | 57.5 |
| scaffold44_4937 | JN901750 | F:GACACACAATTTTCCCAGTGACC  R:CTACATCAAAGCGTGGTAGCG | 57.5 |
| scaffold63_1656 | JN901751 | F:CCATAGAGGGAGGTCACAGTC  R:GATGACCTAATTTCCCTTTGGGGA | 57.5 |
| scaffold63_1632 | JN901752 | F:GGTTCTGTGTACACTCCCGT  R:GGGTGATAGGGTGGTAGATGG | 57.5 |
| scaffold681_58439 | JN901753 | F:TCTGACTGTGTGCTGTGAGG  R:CAGATCTGCAGTGTGTGTGC | 57.5 |
| scaffold697_59562 | JN901754 | F:AATGTTGCCAACCCTTTAGGC  R:ACCTGTAGCTCCCAGTTCAC | 57.5 |
| scaffold743_61690 | JN901755 | F:GCACACAAGGTTATTGGATTGCTC  R:CTTGACAATTCAGGTCCTTGGC | 57.5 |
| scaffold743_61681 | JN901756 | F:TGCTCAGTCTTCTCTGTTCCC  R:AACCACCTCAAGGTAAACCCA | 57.5 |
| scaffold817_65532 | JN901757 | F:TCACAGAGCCTTAAACACAATGGA  R:TATCCTCAGATGGGTATGCAAGG | 57.5 |
| scaffold935_5320 | JN901758 | F:CAGCCTTGTCATATTGTCACAGC  R:CTTTCACACGTATGCAGGGG | 57.5 |
| scaffold944_70593 | JN901759 | F:CTCAACCAACCTGCCTGAC  R:CAATTTACCATGGATGCTAGGACAC | 57.5 |
| scaffold1102_74666 | JN901760 | F:GTTTGTGGTGTGGAAGTAAAGCA  R:CTGTTCTGCCTGTTGTGGATG | 57.5 |
| scaffold147_17514 | JN901761 | F:GCAAAACCTGTTTACCTCACTG  R:TGCCATTAACAGCCACAATG | 55.5 |
| scaffold192_24189 | JN901762 | F:AGGAGTCAGAGGATGGTGC  R:GCCTCAGACCTCAGTCCAT | 57.5 |
| scaffold192_24152 | JN901763 | F:AGCCATCTACTAGGGCAAGAC  R:GTGTAGCTCATTGCTTGTGCA | 57.5 |
| scaffold192_23107 | JN901764 | F:GTGAGAACTCAAACCTAATGGGC  R:CACATACAGAGTGCACAGCATC | 57.5 |
| scaffold192_23105 | JN901765 | F:GCATTATCTTCTTCGGCAGACCT  R:GACTAAATGTCAGCTCCAGGCA | 57.5 |
| scaffold231_26679 | JN901766 | F:AGGAAATTGCTGTGTATCGCTGA  R:CCACCACAAAACACACTGGAG | 57.5 |
| scaffold24_2845 | JN901767 | F:AACCCTGTGGACCTGTTGA  R:CTCCCTGAGCCGTAAGGT | 57.5 |
| scaffold262_29837 | JN901768 | F:GTGTCCTGACAGCTCTCTC  R:AGAAATCAATAGCTGAATCCCTGC | 56 |
| scaffold493_2529 | JN901769 | F:GAAAGCTGATGTCCTGGCTG  R:TGCATGTTGATGAGACAACTCTAGG | 57.5 |
| scaffold582_3194 | JN901770 | F:CGGGATTTGCTGACATCATCC  R:AGTTTCAATCTCCTGACGTGTCG | 57.5 |
| scaffold642_56492 | JN901771 | F:ACCCAATGTCTGTCTCCAAGAC  R:AGAATGACATTGATGGGCCTGT | 57.5 |
| scaffold642_56463 | JN901772 | F:CACTTGCGAACATTGCCATGATAG  R:TCTTCACGCAAACATGCACAC | 57.5 |
| scaffold648_56682 | JN901773 | F:CTGGCCACTTTCATGGAGTC  R:GAGTGAAAGTAAACCAGAAGCCGA | 57.5 |
| scaffold778_63585 | JN901774 | F:GTCTGTTCAAACAACCCCGTC  R:GAAGGAGATAAAGGCAGAGTGAGG | 57.5 |
| scaffold79_8198 | JN901775 | F:GTTTTCCCTCCCTGTGTTTGTC  R:ATGCAGCCAACCTGTTAATCC | 57.5 |
| scaffold863_67732 | JN901776 | F:ACAGGTCACAGAGGATGAGC  R:CGAAACTGTCGTCATAGGAGCA | 57.5 |
| scaffold112_3873 | JN901777 | F:GGGATACAGGACACCCTAAGTG  R:CGCCCACATTGAGCCTATTG | 57.5 |
| scaffold112_3864 | JN901778 | F:CCAAGTTCATCCAGCGTGTC  R:ATGTACAGGCAGCTTTCACCT | 57.5 |
| scaffold1188_76351 | JN901779 | F:GGATGAATGGCAGGAGTCGA  R:TGATGGAGACACACATCCAGG | 57.5 |
| scaffold54_7399 | JN901780 | F:AGGTACAAGCATGCACACTGA  R:GCAGGAGTGCAGTTCAGTC | 57.5 |
| scaffold920_69839 | JN901781 | F:CACCTGACATTTCTTACATTGCTGTG  R:TATTGTGGCAGTCTGGCTGT | 57.5 |
| scaffold1166_75957 | JN901782 | F:TAGGCTGGGTCAGCTCTTC  R:GCTGTTGGAGTTGCTGGTT | 57.5 |
| scaffold125_5738 | JN901783 | F:CAGTCATGCACCATAACGCAC  R:TCAGAGTGAATGGGCTTCTGTC | 57.5 |
| scaffold172_24561 | JN901784 | F:AACGGGAAACAGATGACAATGA  R:GCAGCAGATCTCTGTATGGG | 56 |
| scaffold373_41742 | JN901785 | F:GCAATATCACAGTGTGCAGTCTC  R:GGGTCTGTGTGAGTGTCTGT | 57.5 |
| scaffold682_58456 | JN901786 | F:GTGGAAACTAGAACTGATGGGC  R:GATGGTCAGACCTGTTGTGC | 57.5 |
| scaffold1056_73698 | JN901787 | F:CTCGTTCAGCTCCACAAACAC  R:CACTGCGTTCATGTAGCGTTG | 57.5 |
| scaffold1083_74266 | JN901788 | F:TGGCCCAGAAGAATAGGTAGGA  R:GCTGTTGTGAAGCAAGGTGT | 57.5 |
| scaffold1083_74260 | JN901789 | F:CTGTGAGAGCTGGTTTCAACC  R:ACACACTGATCACAGGGGAG | 57.5 |
| scaffold337_37032 | JN901790 | F:CCGGTCTATAGAAGGCCAC  R:TATCTCTACATCACCCCATGGACA | 57.5 |
| scaffold119_13331 | JN901791 | F:TCTACATCACCCTGGCTTCAC  R:GAAAGGTCATTGAGATTGTTCCCG | 57.5 |
| scaffold1380_78437 | JN901792 | F:CATGATGATCCATCTGTCACTGC  R:CAAACTGGGAGTGACTGTTCC | 57 |
| scaffold150_17758 | JN901793 | F:CTGCAGGTCTGCAATAGGAGT  R:TGAGAGGATGTGTGTGTTGTCC | 57.5 |
| scaffold150_17698 | JN901794 | F:GTCAATGAGCCCAAGACACAC  R:GTTCAAACAGGCCCATAGGTC | 57.5 |
| scaffold230_680 | JN901795 | F:TGGCAAATACATCCTTGGCTCA  R:TCAGCAATCTGAGCAGGTGTT | 57.5 |
| scaffold575_56471 | JN901796 | F:TCCAGTGAGTCATTAAAGGAGC  R:CACACTCTGCAGCTCAAAC | 57.5 |
| scaffold799_5018 | JN901797 | F:TGAGGTGCCAGACGAGTTC  R:GTACGACCTTCTACTGGTGACG | 57.5 |
| scaffold97_165 | JN901798 | F:GTACTAGGTCATGGATGAACCTCC  R:GAATGAAGGGTATGGGTGGC | 57.5 |
| scaffold1159_75853 | JN901799 | F:CTGCCAGTAACTGAATTTGTGCTG  R:AGCTGAATGATTACATGTGTGC | 57.5 |
| scaffold159_18917 | JN901800 | F:CATGCTGTAGAGTCCTGCAATG  R:TCAGACTCAGAGGTTGGAAGC | 57.5 |
| scaffold161_321 | JN901801 | F:ACCCATTCTACCCAAGGACTG  R:ACAGTGAATGTTTCCTTGTTGGG | 57.5 |
| scaffold173_20744 | JN901802 | F:GGTATTCAGCCGACGCTG  R:CCTAATGACTGTTGCTTGATGGC | 57.5 |
| scaffold176_20907 | JN901803 | F:TGCTATGAGCAGTTGCTGTTGA  R:AGGGTAAAAGTGGGGGTTAGG | 57.5 |
| scaffold329_37433 | JN901804 | F:GTCAGGTCCGGATTGACTAGG  R:ATGTACTTCGCCTGAGCTACC | 57.5 |
| scaffold433_2077 | JN901805 | F:CTCATTGTGGGAACTGTTGGG  R:CGCTGTAGAATGCTGTGGTTG | 57.5 |
| scaffold826_5002 | JN901806 | F:GTGGAACACTAAGCCAAACATGG  R:TCCATTAGCAGTGTCCTTCAGC | 57.5 |
| scaffold880_68444 | JN901807 | F:CTGTTCAGTGCTGTGAGAGC  R:TCAAGTGCTTACGTTGCCCT | 57.5 |
| scaffold96_11232 | JN901808 | F:AGGTGTGGAAATAGAGAGAGAC  R:GTCATAGGTCTAGGACAGCATAGC | 57.5 |
| scaffold96_11231 | JN901809 | F:GCTGGAACACAACCATCTTGTC  R:TGGTCAAAATCTGTCCCTGCAA | 57.5 |
| scaffold96_11226 | JN901810 | F:ATTCCACTTGCTGATTGATTACCAC  R:GCCCTCCCACTGGTTTATCT | 57.5 |
| scaffold337_37032 | JN901811 | F:CCGGTCTATAGAAGGCCAC  R:TATCTCTACATCACCCCATGGACA | 57.5 |
| scaffold1130_75208 | JN901812 | F:CCTGGAGAATTCAGTTTGGGC  R:CAGAAACACGTCCAGTCATTCC | 57.5 |
| scaffold207_23021 | JN901813 | F:CCCAAACAAAGTAGTGGGTATGC  R:CACTGGACAAATACCTCCGTCA | 57.5 |
| scaffold207_23035 | JN901814 | F:GAACAGATGTTGTTGCCCAGAG  R:TGCATGGAAGGAGCTAAACACA | 57.5 |
| scaffold241_32555 | JN901815 | F:GTAGAAGGCATCCAGCGC  R:TCATCTGTCAGAGTCCCGTG | 57.5 |
| scaffold453_45037 | JN901816 | F:GTCTCACCAGAAACAGGCC  R:GACACATTATTTCCATCCGTGCG | 57.5 |
| scaffold718_4402 | JN901817 | F:TCAGCAGGTCTGACTGCAA  R:CCGTCATCTTTTGTGTTGCTTTACTC | 57.5 |
| scaffold718_4401 | JN901818 | F:CTGGTGCTGATCCTGAGC  R:CAACCCATTTGTAGTGGAGGC | 57.5 |
| scaffold731_61197 | JN901819 | F:CACCAATCAGACCTCACAAAACAG  R:TGAGTGTTTTCCCCACACATACTG | 57.5 |
| scaffold1029_72973 | JN901820 | F:GGCCCTTGTCACATTGGTAG  R:GTGCAATGAATTGTGTTGGCATCT | 57.5 |
| scaffold317_37380 | JN901821 | F:CATCGTTGCTGACTTCCTGTTC  R:GTCTTCGTCCCACAGATAGAGAAC | 57.5 |
| scaffold249_28563 | JN901822 | F:AGCACCTTTATTGAAATCGTCAGC  R:CTGATTGGCCGAGATAACTTATGG | 56.5 |
| scaffold389_1657 | JN901823 | F:AGTGAGGAAGATGAGGCTTTCC  R:CACCACAAGCAGCAGTAGC | 57.5 |
| scaffold864_67778 | JN901824 | F:TGGCCTCTCTATGCTTGTGAG  R:TCCGGACATCCATCATTGCT | 57.5 |
| scaffold1057_73711 | JN901825 | F:TCAACATCCATGAGGACAGTCAG  R:TGATGCATTGACATTCAATGAGCC | 57.5 |
| scaffold1101_74644 | JN901826 | F:ACCTGATGATGAAGTGCACACA  R:CCGCTGCTCCTCTGTATTTG | 57.5 |
| scaffold32_4004 | JN901827 | F:CAGGTCTCCTTTGTAAGCTTTTCAG  R:GCATGTTGTGGTTTGCAGTG | 57.5 |
| scaffold665_57470 | JN901828 | F:TTCAGACACCAGAACCAACCA  R:GCCTGAATCAACCAGACACC | 57.5 |
| scaffold699_59679 | JN901829 | F:CTGAGCATTGTGAATGGAGATTCC  R:CTTACTTTGGGGCCTGTGTAC | 57.5 |
| scaffold866_67926 | JN901830 | F:CCAACGGACACAACAAGCT  R:GTCAGCCTATCAGAGGGCA | 57.5 |
| scaffold1189_76355 | JN901831 | F:TCACAGTAACGCTCCTCACATCTC  R:GGTCTGATTTACTGTGTGGCTGAA | 57.5 |
| scaffold1201_76594 | JN901832 | F:ACCTGACATCACAATGTGTCTCC  R:AAAACAGTACGGGGCGTTTCT | 57.5 |
| scaffold1219_76726 | JN901833 | F:ACCTGCATACAATCCATACTCGTG  R:CAGAATTTGAGGGGAATTTCAGACC | 57.5 |
| scaffold145_17427 | JN901834 | F:TGTGGCAAATAAACCAAGTGCTC  R:TGAAACATCTCTGATGTGCATGTCA | 57.5 |
| scaffold353_40093 | JN901835 | F:CTAACACTGTTGGTTGCTCCG  R:TCTGAGTTTGAAGATGTGTCTGATGA | 57 |
| scaffold489_24067 | JN901836 | F:TCTTACCCAAGGGAACCACAC  R:TCTGACGGCATAGTGAAGCTG | 57.5 |
| scaffold525_49695 | JN901837 | F:ATCACAACAAGGTGACGTGCT  R:CATGAGAGTGTGTCGCTCTGT | 57.5 |
| scaffold57_6518 | JN901838 | F:GGATCCACACACTTAAGGTTGACA  R:GTCTTCAATCCGCCTCAGTCA | 57.5 |
| scaffold654_57104 | JN901839 | F:CACTCATTAGACACAGCAGCAATG  R:CGAGATCCTCAATCAGGGGTAG | 57.5 |
| scaffold71_8411 | JN901840 | F:TCTGATTCTACTTTAGGCGATGA  R:CCTTTGTCAGATGATAAGAGCAG | 54 |
| scaffold1085_74303 | JN901841 | F:TTGTATCCACGTCCGCACT  R:CAACGCCATGATGTTCACACT | 57.5 |
| scaffold173_479 | JN901842 | F:TGAGGTCACTCCATCACACC  R:GACCAAATATCCACCCAATGTCC | 57.5 |
| scaffold55_6429 | JN901843 | F:GTCTCCTTCCCTCTTGAACTGT  R:TTCTCCGGCTGACCTCAATAAC | 57.5 |
| scaffold651_3890 | JN901844 | F:TATACTGCTCATGCCCCACG  R:CTGGAAACGCCTGTGAACC | 57.5 |
| scaffold19_758 | JN901845 | F:TCTATGGAACACACGCGCT  R:GATTTGATGAACAGAGCAGCAGC | 57.5 |
| scaffold29_2656 | JN901846 | F:ACACATGTCCTCTGCCCA  R:TGTCTGAGCAGGGAACCTC | 57.5 |
| scaffold432_44220 | JN901847 | F:CTGGCCCAACTCTTTTCCTC  R:GGCCCAGTTCAGACTGGTA | 57.5 |
| scaffold678_58263 | JN901848 | F:ATCACGACATGTAAAACCTCAGC  R:GAGACATCTGATTTGCATGGAG | 57.5 |
| scaffold901_69220 | JN901849 | F:TCGTAGTTCCATTGTTCAGACCC  R:TCACCAATCAGCTTTGCAGC | 57.5 |
| scaffold1082_74230 | JN901850 | F:TGGTGCATCCTGTCAGAGAG  R:CATGCTCCTCAGAGTGGC | 57.5 |
| scaffold3_6091 | JN901851 | F:TGAGATGTTTACTGCGGAGCT  R:TCGAGCAACAATCACGCAC | 57.5 |
| scaffold153_5996 | JN901852 | F:TCACATGCTGAGGAGTGGAAG  R:TGACCATCGGATTTGGGAAGAG | 57.5 |
| scaffold442_21899 | JN901853 | F:CATCAGCTTCAGTGGCAGAAAG  R:ATTGAGGGTTAAGGACAGAGCC | 57.5 |
| scaffold633_26733 | JN901854 | F:GCCACACAGATTAAGACTGAGGT  R:AGGCCAAACTTATTTTTGCCACC | 57.5 |
| scaffold633_57652 | JN901855 | F:CGCATCTGCCTTATTGTTGATCG  R:CAATGTGACACCTGCAGTCC | 57.5 |
| scaffold123_15748 | JN901856 | F:AACAATCTGTGACTGCTACTGCTT  R:CTGAACAACACACATCCACATCAC | 57.5 |
| scaffold215_25019 | JN901857 | F:GACCTTGCATCATCTGCTTCAG  R:TACATCAACGGCTGCCAGTAT | 57.5 |
| scaffold384_43780 | JN901858 | F:GCTTTCACTTTCTGTCAGTGCC  R:GTCAAATGAGCAGGATGGGG | 57.5 |
| scaffold45_4452 | JN901859 | F:GACATGGACCTGCAGGGATA  R:TATCATGCCACAAAACGGGC | 57.5 |
| scaffold644_60142 | JN901860 | F:TCTGCTCCCAGAGGTGAATC  R:GGTGGAGAACGACTGAGCT | 57.5 |
| scaffold142_5900 | JN901861 | F:AGCATTCATCTAGCTCACAGTCC  R:GTAGCGCTGTGTAGCATGTG | 57.5 |
| scaffold142_5906 | JN901862 | F:CTCAGCTTCTGCTCTGGGA  R:GCAGGCCTATATTCAGTACCCTC | 57.5 |
| scaffold343_40722 | JN901863 | F:AGAAGGATTGATGGAAACCTGT  R:GGGAATAATTCATCCCACTATCCA | 55 |
| scaffold12_1224 | JN901864 | F:CATGAGTCTGGCTGTTGTTCG  R:TTCCATTGCTGCAGCTCTTC | 57.5 |
| scaffold166_9308 | JN901865 | F:GTTAACGAGCCACAAAAGGTCAAG  R:CTACTCCCACAAAATGGGCC | 57.5 |
| scaffold240_27581 | JN901866 | F:TGTCCTTGGTTGTAACAGTGCA  R:TGACACATACTGCCTCATTCACATC | 57.5 |
| scaffold1583_79027 | JN901867 | F:ACATGGAGCGAGATGAGTTCAG  R:CTCGTCTGACTGACGTGACAA | 57.5 |
| scaffold41_1317 | JN901868 | F:AGAGCTGGAACTGAGCTTCC  R:ATAGACACAGAACGCCGACC | 57.5 |
| scaffold66_7460 | JN901869 | F:CTGAAGGAAGAAACATGTCACAGC  R:CAGAATAACACTACCAGAGCGCA | 57.5 |
| scaffold359_38901 | JN901870 | F:CACTGTTGGGGAGCAGATG  R:GGTGACGTTAACTGGCGAG | 57.5 |
| scaffold817_65518 | JN901871 | F:TGATTGTGGATGTTAGTTGATGATGA  R:TGAACAATCACAGCCTCTCC | 55 |
| scaffold154_18733 | JN901872 | F:CTCTCGTGGCTGATTCTTGC  R:CAACAGCAGCCTTGAATGACTT | 57.5 |
| scaffold852_67256 | JN901873 | F:ATCGATCTCAGGTCCGGTTT  R:TGACCGAAACTGTGAATGTCC | 57.5 |
| scaffold11_37 | JN901874 | F:TCTGCACAGGAGTGAACCTATTG  R:CGAAGATCAGAAGATCGCTGTGT | 57.5 |
| scaffold11_39 | JN901875 | F:CAAACTATACCAAACTGCACACGG  R:GTAGGCTTTCCAGCTCTGTCT | 57.5 |
| scaffold970_71274 | JN901876 | F:CAGGCAGGTCACAAACTGATG  R:ACAATGCAACAGATGTCACACG | 57.5 |
| scaffold781_63723 | JN901877 | F:GTCAGATTTGAATGTGCAGCTTTG  R:CTCATTGACTGACTAAATGCGGAA | 57.5 |
| scaffold1531_78955 | JN901878 | F:ATACTGCACTGACCAGGCAT  R:GCAGGCAGAATTGAGGAGATG | 57.5 |
| scaffold3_346 | JN901879 | F:GAACTCAGGCCTACGTTTTCCAG  R:TTCACATGGTAATCAGGGTGTACG | 57.5 |
| scaffold497_48101 | JN901880 | F:CTATGAAAGACTGTAGGGCGAGGA  R:TGTCAAAGCTGTCCCTCTTC | 57.5 |
| scaffold299_33520 | JN901881 | F:AATCTGGTCAACTCCTTTAGCC  R:GCAAGTTCATGTGAATCCCTATTTG | 55.5 |
| scaffold289_32583 | JN901882 | F:TCCCAAGTGAGATACTAAGATC  R:CTTGTGAGTGATTTCTGCC | 53 |
| scaffold145_17330 | JN901883 | F:AAACCTCAGGCGTAAATCCACT  R:CTGAATCACAAGCCTGTCAACAG | 57.5 |
| scaffold150_17697 | JN901884 | F:CCTCCACCAATCAGGTTAGTGTTT  R: ACAGCTCAATGCAATTACAAGCAA | 57.5 |
| scaffold233_27183 | JN901885 | F:CCACCTTAACTAACGGTTTGCTGT  R:TGTCTTAGTTGCTCATTCAGTCCC | 57.5 |
| scaffold1504_78863 | JN901886 | F:GGCCCAAACAGGTTAGTCTGA  R:CAGAGAGGAACGACCAAGCA | 57.5 |
| scaffold1148_75487 | JN901887 | F:AACGTGTGTTGAATCCTGTTGC  R:CTCAATTGATGGATGGATGGATGGA | 57.5 |
| scaffold324_35369 | JN901888 | F:TATTAACGATATGCCCACCCAAAC  R:GTCGTTCATTTCTCCATTTCCATC | 57.5 |
| scaffold1327_78019 | JN901889 | F:GTGTAGGACAAAGCCTCCTCA  R:CGTCGCCTCCAGTCAATG | 57.5 |
| scaffold391_41140 | JN901890 | F:GCCTTCCATAGCTTATTTATCCGC  R:AGTGTTTCATTTTAGTGGGGGACA | 57.5 |
| scaffold807_65092 | JN901891 | F:CCAGAAGGATGATGGTCGTCT  R:GGTGCTCTGTAGGAGGATGAA | 57.5 |
| scaffold857_67425 | JN901892 | F:AAAGGTGGCAGTGTTTTGG  R:TGTGATTTCACTGCATGTGTAGGAT | 57.5 |
| scaffold1162_75882 | JN901893 | F:AAATTCCTCTCTAGCTTCCACACG  R:AGACAATCGCTGGATTTCAAGAAG | 57.5 |
| scaffold1085_74305 | JN901894 | F:AGACAAAGCTCGTCATTATCCTGC  R:AATCAGGTTTAGCAGTTGCCACAT | 57.5 |
| scaffold44_5286 | JN901895 | F:CCAGCATGCATAGTACATCCC  R:GAGATTCCCAGTGAGGGGTT | 57.5 |
| scaffold1015_72650 | JN901896 | F:TCACGTCATGGATAGAGAGATG  R:AGTTTCCCTTTGCAACATAGAG | 54 |
| scaffold147_17515 | JN901897 | F:CACAATGTTATAATTGCAAACGGC  R:GGCATAATTATTCTTTGTTGAGCTTTC | 57.5 |
| scaffold37_4241 | JN901898 | F:TAGTGCCATGGGCTTTTTAGTTGT  R:CTTTTGTCAAGCAAAGCATGTCTC | 57.5 |
| scaffold176_20941 | JN901899 | F:CAGTGCTCTTCAGGGCATATTAGC  R:AGGAGCATGCAAACAGGTCTACTC | 57.5 |
| scaffold663_57372 | JN901900 | F:TACCAGGACTCAGAGGAGAACAGG  R:TGCTGTTGTTCAGGCAAAATACAT | 57.5 |
| scaffold34_5087 | JN901901 | F:CAGAGTGAGTGACTGCCAACACTT  R:CACAAAGACGCCAGATTTTCATT | 57.5 |
| scaffold301_33889 | JN901902 | F:AGCAATTTCTTCCTTGACACACC  R:CCTGAGGAAAGAAACATAATGCCA | 57 |
| scaffold301_33874 | JN901903 | F:AGTGCTTCTTTTGTAAAAGGATGG  R:GTCTGGGCACTCATTTTACTC | 55 |
| scaffold448_44855 | JN901904 | F:TAAAACCATTCTCTCGGTTTTGCT  R:TTCAAATAGACGCAAGGAAGGAAG | 56 |
| scaffold776_63391 | JN901905 | F:TGGGGTGAAGAATGTTTGC  R:TTAGGCTCCTAGTGAGATTGC | 55 |
| scaffold66_7487 | JN901906 | F:CATGTTGTTACCAAGTGTAATCCTGT  R:TGAACAAATATGTATTTTCTTTTTCCAA | 53 |
| scaffold370_39896 | JN901907 | F:CAGATACAAACAAACACGCTCACG  R:ATGTAGGTGAGAGTTGGACCATG | 57.5 |
| scaffold454_45063 | JN901908 | F:GGAAGCAGAGTGTGTTAGTGTGGA  R:CTCGTGCAGTTCAAATTCTGTGTC | 57.5 |
| scaffold636_58399 | JN901909 | F:AACGCCAAAGATAACAAGTGGTTG  R:GGACTGAAGGGAGTCACCAGAGTA | 57.5 |
| scaffold1197_76522 | JN901910 | F:TCGGACTGTGGGATTTTCTCTAAG  R:CTATCTCCATGGGAAAAAGGCTTC | 57.5 |
| scaffold343_37429 | JN901911 | F:CCCTGAATGATGGACTGACAGTAA  R:GAGAGGGACAGAATATACCAGGGG | 57.5 |
| scaffold1362_78376 | JN901912 | F:TCGACAAGGAAAACAAGCATATCA  R:CACAGCCTACCATCATCATTTATCA | 57.5 |
| scaffold370_41021 | JN901913 | F:ATACAAGCAGAGGCATCAATGTCA  R:ACTGACGTCTTGCTGATAGCAGTG | 57.5 |
| scaffold1033_73073 | JN901914 | F:GTAAAGCTGTGCACAATGATCTGC  R:AGCCAGACTGATATGACGGAC | 57.5 |
| scaffold296_33264 | JN901915 | F:GACAGCTGAAGCCTGTTAACGA  R:GATCCTGTGACACAAACCAGC | 57.5 |
| scaffold660_57301 | JN901916 | F:GAGTAATCGTCCTGTTTCGCTCC  R:CTGTGACATGGCATTTATCTCCAG | 57.5 |
| scaffold632_57812 | JN901917 | F:TGTCCAATAGATGGCAGCAGAG  R:ATTGGACAAAGTGAACAACTGGC | 57.5 |
| scaffold77_372 | JN901918 | F:TGCCCTCTTCCTCTCACAC  R:CCCTCTAATCCTGCAGTGTACC | 57.5 |
| scaffold395_46627 | JN901919 | F:TGTGCTGTACTCAAGTGTTTCCTCTC  R:TGATCACAAACTACAAACGTCAGACA | 57.5 |
| scaffold345_39382 | JN901920 | F:AAATGTTTCACATTCCTCCTCCCT  R:AGAGGAAAAAGGGGATTGAGAGTG | 57.5 |
| scaffold304_44662 | JN901921 | F:CTCCCTGCCTACTCCTGCTCT  R:TCAGAGGAAAAAGAGAGATGAGG | 57.5 |
| scaffold505_53887 | JN901922 | F:TGATGATGTGAATCTTGACAGATAGC  R:AAATTATTGAGCCTTTTGGAAACAA | 57.5 |
| scaffold514_55541 | JN901923 | F:TGACGGGAAATGTGAATATGTGTT  R:CGATTCATCCTCTACTCTCATGTCAA | 57.5 |
| scaffold1044_73520 | JN901924 | F:AAAAGGACACACACATCGTGG  R:GTATGCATGTGTACCGAGAGC | 57.5 |
| scaffold927_70003 | JN901925 | F:TCAAACACATACGTTGTGCAATAGC  R:TCACTTCTCTGAATGGGGGAATG | 57.5 |
| scaffold79_10045 | JN901926 | F:GAAATACACAGAGCAGGGGAAC  R:GAGAGCACAGATGTCGTGC | 57.5 |
| scaffold75_9579 | JN901927 | F:GCTGATGCAACTTCAGCCT  R:AGTGTGGAAGCGTCAAGGT | 57.5 |
| scaffold966_71247 | JN901928 | F:CATCCACATGACTGCCACTG  R:CGCACAACCTGCAGAAGTT | 57.5 |
| scaffold1110_74816 | JN901929 | F:ACAGCTTGTAAGACTCCCTGAC  R:CTGCGGTGTGCAGCATAAAT | 57.5 |
| scaffold968_71269 | JN901930 | F:CTGCACCTGCGGAGATAAGA  R:TGTGTCAAACCAGACGACGA | 57.5 |
| scaffold1598_79036 | JN901931 | F:AGCATGAACACCAAGGTACACA  R:GTGCAGATTCAGCCAGGAG | 57.5 |
| scaffold536_50439 | JN901932 | F:AGTAGTCTTGTTATCCACGCCAG  R:GTGCACGTTTCTCACACATCTG | 57.5 |
| scaffold1419_78626 | JN901933 | F:TGATGAAGCTTTGAGGCAACTCT  R:CTGTTGCTGAAACCCCATTGAT | 57.5 |
| scaffold1211_76670 | JN901934 | F:GTTCCTGCTTCAATCCCTGAC  R:ACCGAAGTCGCATGAAACTCT | 57.5 |
| scaffold893_68883 | JN901935 | F:GACCACTGTGACATTTCCCTG  R:AAGACGTAACACCGTGCTGA | 57.5 |
| scaffold15_7596 | JN901936 | F:GGTGCTCACAACACTCCC  R:TGACTCCAGACTTCGTGTGG | 57.5 |
| scaffold21_2896 | JN901937 | F: ATATTTGTGAAGCCGCCGC  R: GACAAAGCGAATACAGGCGTG | 56 |
| scaffold212_29511 | JN901938 | F:TGTGACAGCCAGGGTCTAC  R:CTGCAGCAGGGTTTCAGG | 57.5 |
| scaffold99_12690 | JN901939 | F:CATAGTCAGGGTTTTAGTGTGGTTC  R:TTTTTGTCTAAGTTGGAAAGCGA | 57.5 |
| scaffold380_42370 | JN901940 | F:CTCCATACTGGAGTTGCCTGTGTA  R:TTTAATTGACCCTTCCTTCCTGCT | 57.5 |
| scaffold846_66755 | JN901941 | F:CGATACTGTCTGAATGTCCAGGTT  R:GACGAGGCTGATCACACTTTG | 57.5 |
| scaffold253_29287 | JN901942 | F:CCTCCATGTTAGTGGATGGGA  R:TCACTGACAGATCCACCCTG | 57.5 |
| scaffold362_39390 | JN901943 | F:ATGTTTGTGTTTGACTGTGGGTC  R:CCCTACTATCACATCTAGGGAATGG | 57.5 |
| scaffold1580_79021 | JN901944 | F:ACACCTTCATGTCCCTGTAGAG  R:CCATTCATCCATGTGAGCCAC | 57.5 |
| scaffold1200_76580 | JN901945 | F:GCTGATACCTCGGTCCTGAAG  R:CTCTGCGTAAAAGTGGTCCAGTAG | 57.5 |
| scaffold386_40808 | JN901946 | F:ATCCAGGCACAGACGGATAG  R:AGTGTTCTGACTGAGTGGTTCC | 57.5 |
| scaffold1697_79099 | JN901947 | F:CGGCGAGATGAACAACCTG  R:TCGGTCGTTACCTATGACGG | 57.5 |
| scaffold855_61118 | JN901948 | F: CTATTGCCACCCAACCCTC  R: AGGCCTCAAGACTCTCACG | 57.5 |
| scaffold332_47850 | JN901949 | F:TGGGACATACACAGCTGACC  R: TGCATACAGAGCAGTCCCTC | 57.5 |
| scaffold474_51719 | JN901950 | F: CGAGGCTGAACAGATGCTG  R: GTGGTGTGAAAGAGGAACGTG | 57.5 |
| scaffold89_8557 | JN901951 | F:GAGGAAGGAGGAAGTTGCTAGGAC  R:GTGAGGTCAGGGCAAATTAGACAG | 57.5 |
| scaffold434_49783 | JN901952 | F:CAGCAAAATGCTGCATAGAGAGAA  R:CATGGAATCATCACTCACATGGAT | 57.5 |
| scaffold86_10649 | JN901953 | F:GGCAGACACTAACACAGCTTTTAC  R:CCAAGAACAACGACCTGGG | 57.5 |
| scaffold288_32413 | JN901954 | F:ACCAGGGAGTCGGTTTGATAC  R:CGGTCGATCTCATTCACACACT | 57.5 |
| scaffold925_69991 | JN901955 | F:CCTAGGACTTGAGGGAAAGGT  R:ACCAACTGAACCCACCATCT | 57.5 |
| scaffold80_10075 | JN901956 | F:TCCCTAGTCCCTGGAGAGT  R:CCTCATGCAGGGATCACAATG | 57.5 |
| scaffold1411_78584 | JN901957 | F:GCGTAAATCGGAGGAAACATTCG  R:GCATTTCATCGCAGGCTACAG | 57.5 |
| scaffold902_69225 | JN901958 | F:TGATGGTTTAGGTGTCCAACGT  R:TGAGCTAGTCGTAGCTTGAGC | 57.5 |
| scaffold1412_78595 | JN901959 | F:TCTGAGCAGTGTGTGAGGAG  R:CTGTGTGCTGGGTGTGATG | 57.5 |
| scaffold1557_78997 | JN901960 | F:GACAGGAACCTTCAGGCAAC  R:CGTCTGTGTCAATTGCTCTGG | 57.5 |
| scaffold699_59691 | JN901961 | F:ACTGTGGATGAGGCCAAAACA  R:TGCACACAAACAACATCAAGCTG | 57.5 |
| scaffold669_57803 | JN901962 | F:AGGGTTTTTGAAGCCAAAGTCAC  R:AGCAATCTCTGAAATGCAGTCGA | 57.5 |
| scaffold166_19955 | JN901963 | F:GCCTTAAAGACAGCCAAGAGC  R:TGAACTGGCATACGTGTTAGCA | 57.5 |
| scaffold967_71261 | JN901964 | F:AGAAAACAGACTCGGTGACTGG  R:GGATCCTTCTCAAAGCGTCAGT | 57.5 |
| scaffold652_56830 | JN901965 | F:TTCACAAACATGCACACAATCCTG  R:TGGAGGAACAGAAGAAGCCTG | 57.5 |
| scaffold868_67968 | JN901966 | F:GGGTGATCAGTAAACTTGGCG  R:ACACCTTCAGTCTGAGTCCAAG | 57.5 |
| scaffold670_57883 | JN901967 | F:CAAATTTCTGATCCTGCCTTCAGC  R:CGTCAATCAGGAAGAGTGCGA | 57.5 |
| scaffold573_52741 | JN901968 | F:ACACACACTCAATATGTGCCAGT  R:ACTCAGTGGTCTCGTGAGC | 57.5 |
| scaffold618_55289 | JN901969 | F:CTCTCCAGTCCTGTTCCTCATTC  R:AGAGTCTCCAGCCACCTTG | 57.5 |
| scaffold931_70316 | JN901970 | F:GGCTGTTCATTAAACTCTGTGCC  R:ACACTGTATCATGTAAACAGAGCGG | 57.5 |
| scaffold1484_78802 | JN901971 | F:TATCACAGGTCACACACTGGG  R:CAGGCGTCCTCTGGATTACTAC | 57.5 |
| scaffold1268_77444 | JN901972 | F:AACGAGGTTACGAGGGGAAAC  R:CCTCAACTTCGTCTTGCACAG | 57.5 |
| scaffold1237_77013 | JN901973 | F:AGATGCAAATGCAGGGGAATG  R:CACATTTTGAGACTCCCCCTCT | 57.5 |
| scaffold565_52180 | JN901974 | F:GATGACAGGAGGACCTCTAATGC  R:TGTCAGTCCACTGCATCTGG | 57.5 |
| scaffold1040_73380 | JN901975 | F:AACGCTGACAGGGGTCTTAC  R:GATAACCAGCTACAGGGGCAT | 57.5 |
| scaffold285_32129 | JN901976 | F:GCTGACAATGTCTCCCACG  R:GGAACAGATTCATCCCAAGGC | 57.5 |
| scaffold380_40402 | JN901977 | F:TATCATGAAGGAGGCACCAGC  R:TTGAGACATTACAGCGCCACTT | 57.5 |
| scaffold949_70835 | JN901978 | F:GTGTAGGTAGTACTCATGTTGTGAGG  R:GCCAATGGCAGAGATACAGC | 57.5 |
| scaffold553_51199 | JN901979 | F:AGGATGAGACCTCACCACAG  R:CACAACCAGAGGAAGCCAATC | 57.5 |
| scaffold553_51257 | JN901980 | F:GAGCCTGACCAATTCCTGTTC  R:TCAGCTGTTTGTTGGCATGAG | 57.5 |
| scaffold350_38166 | JN901981 | F:TCTGAAGCTGACAACAGGCT  R:ACTCAGCCCCATAATCTTGCA | 57.5 |
| scaffold593_53878 | JN901982 | F:ACCACCATCCAACTCATGCT  R:TCACGACCTGTCCCTTCAC | 57.5 |
| scaffold1272_77518 | JN901983 | F:GAAAGCAGATGGCAGACAGAC  R:TGTAGACAAACCGGTGGACTG | 57.5 |
| scaffold1473_78787 | JN901984 | F:CTAGGAAACACGTCTCCTGCA  R:TCCTACTATGCCCACAAATCCAG | 57.5 |
| scaffold432_52502 | JN901985 | F:CCTGCAAAATCAGCCCTTAGC  R:TGGTCCAGATAGACTGTTGCATTG | 57.5 |
| scaffold695_59522 | JN901986 | F:GTGTGACGGAAAGCACATAACG  R:TCTACCCACTCTTGGTGGC | 57.5 |
| scaffold1481_78796 | JN901987 | F:TCGACACAAACATCTGGACATTCAG  R:AAGGGACAGTCGGTGTACTG | 57.5 |
| scaffold782_63754 | JN901988 | F:CTCAATACCTCTGGGGACTCTG  R:TCTGCTCTGGAAGCTGTGATC | 57.5 |
| scaffold1065_73943 | JN901989 | F:ACGACGAGATCACAGCTCAC  R:TATCATGCCCACAACCTACCAG | 57.5 |
| scaffold603_54567 | JN901990 | F:TTCTGGTGGCTACATGACTGG  R:AAAGCCAGGTCTCATGTTGGT | 57.5 |
| scaffold883_68559 | JN901991 | F:TGCGGAACATCGTGGTTTG  R:GAAGCACAAGGCCACATACAG | 57.5 |
| scaffold788_64026 | JN901992 | F:CAGGATCTACCAGGAGGAACTG  R:CCGATAAACCAGCGCATGAATC | 57.5 |
| scaffold14_1332 | JN901993 | F:CCACTCACTAGACGCTGTGT  R:ACACTTGTGTACGGGATGAGAC | 57.5 |
| scaffold208_24348 | JN901994 | F:CTTCTGGATGACACAGAGAGGAG  R:CAAGGCTAAGTGATTGTTTTCAGGG | 57.5 |
| scaffold557_51607 | JN901995 | F:CTTCTGGATGACACAGAGAGGAG  R:CAAGGCTAAGTGATTGTTTTCAGGG | 57.5 |
| scaffold1397_78546 | JN901996 | F:TTACGTCACCGTTAGCAGACTC  R:GTTTTCAGCAGATCCATCAGAGC | 57.5 |
| scaffold1334_78095 | JN901997 | F:ACTGAGTCTTCGCTGAGTGG  R:GCTGAGGTCAGGTACATCCA | 57.5 |
| scaffold308_34515 | JN901998 | F:CACAGGACATTTCGACTGGC  R:CAGAAACATCCGCACTCACAC | 57.5 |
| scaffold730_61157 | JN901999 | F:ATCCACTGAGTGATAGCGCC  R:GATTAGGCAAACCTGAGTGCAG | 57.5 |
| scaffold661_57326 | JN902000 | F:TTGGAAAACACGTCCGTCCA  R:AGAGTCAAGATCTGTCCGCTG | 57.5 |
| scaffold1357_78359 | JN902001 | F:CTCGGCTAATCACTTCCTACAGG  R:TGTTGCACTACCTGAGGCATT | 57.5 |
| scaffold655_57146 | JN902002 | F:GAGGTAGGGTTCTACCGCTG  R:AAGAGGCCACATACACGACTG | 57.5 |
| scaffold1403_78572 | JN902003 | F:AGCTCCAGGACATTCCCAG  R:CCCTTCACTTACAGTCGAGGAG | 57.5 |
| scaffold17_1771 | JN902004 | F:TCTATACAATGGTGGCAAGGAAGC  R:TCCACCGAAGCATAACTCACAG | 57.5 |
| scaffold27_3060 | JN902005 | F:CCACTGAATGAGACAGGTTTGC  R:CGGAATTGGAATTGTGGACTGG | 57.5 |
| scaffold45_5399 | JN902006 | F:GGTCATGTGACGTGTTTTCAGG  R:CAAAGACGTTCATCGTGTCGC | 57.5 |
| scaffold434_21719 | JN902007 | F:AAGCTCAGCAAAATGCTGCATAG  R:TCATTAGGCGGAAGCATGACTC | 57.5 |
| scaffold558_26196 | JN902008 | F:TCCTATGATCCTCCAGGACACA  R:CAGTTGCTCTCGCCTACAGA | 57.5 |
| scaffold511_25688 | JN902009 | F:CTTCGGTTCTGTCAGCAGC  R:GTTGATGTCTTCACTTGCCTGG | 57.5 |
| scaffold355_17668 | JN902010 | F:TGCTACTGAGTCCTGGTTCAC  R:GCACACAGAGAGCACATGG | 57.5 |
| scaffold59_1611 | JN902011 | F:TCTGGTAACTGTGGTGAACAACC  R:AGTGTTTGGCAGCGTGTAAGA | 57.5 |
| scaffold135_4875 | JN902012 | F:ACTGTCGTCCAAACTGGCA  R:TGTCCCATCTGCTAACATGGAG | 57.5 |
| scaffold343_17656 | JN902013 | F:CATTGTACACACATCGTGAGTCCT  R:TTGTCCAGTTTCCAGTTGGGA | 57.5 |
| scaffold636_27020 | JN902014 | F:CTTCACTCACCCAAGGAGCT  R:GGGACAGAAAACAGTGTTGCAG | 57.5 |
| scaffold483_24205 | JN902015 | F:GTTGTTTTGAGGAGGCCAGG  R:TCCCACGTAACTTGTGGAAGG | 57.5 |
| scaffold181_12069 | JN902016 | F:CACAGCCATGTATGAGGTGACT  R:GATTGACCCGGAGTGAAATCGA | 57.5 |
| scaffold381_19761 | JN902017 | F:GCTGGAATTAGCTCAAGCCC  R:GATCATGGCCAGCTTGTCC | 57.5 |
| scaffold196_10495 | JN902018 | F:CAGGTCAGCTCAGCAAAGC  R:CCAGGTGACTAATCCAGACAGG | 57.5 |
| scaffold576_26595 | JN902019 | F:AGGCTGTACAATGTCCCTGATG  R:TTAGCTGATAGGAGACGCGC | 57.5 |
| scaffold20_126 | JN902020 | F:ACTGGGAAATGATTGCAGGTCT  R:TGGCCCTAAATGCTGACGT | 57.5 |
| scaffold20_119 | JN902021 | F:AGTCAGCAACATGGGCTAAACA  R:TAAATCAAAGCATGGCCGCAAG | 57.5 |
| scaffold441_24747 | JN902022 | F:GTTTGGCATGAACAGTGTAGTTGG  R:GGAGGAGGTAATAGCACGCAT | 57.5 |
| scaffold326_16280 | JN902023 | F:AGGAAGCAGAGGATCATGGG  R:TGTCACGTACGAACAAGTGGAG | 57.5 |
| scaffold745_61779 | JN902024 | F:GATTCATTCCGTGTCGAAGCTG  R:AGAGCACACAAATCATCGAGTGT | 57.5 |
| scaffold265_30001 | JN902025 | F:AGCACAGAGAGCACCTGAG  R:CACACAGATCTGCCAGCC | 57.5 |
| scaffold499_48170 | JN902026 | F:TTTCCAGAGTGAAGGTGCAGAG  R:TGTCAAGTCTGGATTAGCACACC | 57.5 |
| scaffold703_4284 | JN902027 | F:ACTTCTTCAGACGGGACATCC  R:TCATCATAGAGTGTATACAGGCAGCA | 57.5 |
| scaffold803_4937 | JN902028 | F:TTATGCAACACATGCCAGCC  R:GTCATCATCATCCTTTGGGCAG | 57.5 |
| scaffold803_4932 | JN902029 | F:AGCTGGTAAGCCTCTCGATG  R:CAGACAGACAACGGAACAACC | 57.5 |
| scaffold727_4460 | JN902030 | F:CAACCAGAGACGACGATGC  R:GATGTCACACCTTGTTAAAGCCC | 57.5 |
| scaffold935_5323 | JN902031 | F:GAATCTGTATCTTGCACACAGGC  R:CGTAACAGATTCAGTCATGCGTTG | 57.5 |
| scaffold208_10886 | JN902032 | F:GAAGAAAGCTGCAGACGAAACC  R:GTGAGCCCAAGTCATTTTAAGCG | 57.5 |
| scaffold288_14923 | JN902033 | F:CACATCAAGCTGTCACTCAACC  R:GTCTAATGTGCAGCACCCTG | 57.5 |
| scaffold563_26094 | JN902034 | F:ACTGTGAACGAATCCCACAACA  R:TGAGACAGTAATGGGGAGGC | 57.5 |
| scaffold252_13433 | JN902035 | F:GGATGTTGGAATGTGAGCAGG  R:GGGCCACTTATACTAAGTCGGG | 57.5 |
| scaffold416_20702 | JN902036 | F:TGCTGCTCTCTGATGGGAG  R:AACCACTCAAAGCTGTTGCC | 57.5 |
| scaffold481_23900 | JN902037 | F:GCTGGTCTTGGGTAATGTGC  R:TGTCACTTGTTCAGTGGCTTCA | 57.5 |
| scaffold242_12682 | JN902038 | F:CTGTTGTCTCAAGCCACCC  R:CCTGAGAACAACTGTCCATGC | 57.5 |
| scaffold41_1320 | JN902039 | F:ATATGCCCTTCACAGTCAGGG  R:GTGTAGCCAATGACAAGGAAGC | 57.5 |
| scaffold855_27657 | JN902040 | F:CACTCTGTCACTTCCTCCTCC  R:GTTCCAATTAACCAGCCCACAAG | 57.5 |
| scaffold855_27659 | JN902041 | F:CCTGGTCTCGGCTTGATATGTA  R:CTGTTGCCAGGAATCGTAGGA | 57.5 |
| scaffold248_13005 | JN902042 | F:GATGAGACAACCTGCACAGC  R:GCATTCAGGTTCCAGGAAGTG | 57.5 |
| scaffold502_24311 | JN902043 | F:TTGTGGTCCATACCTGTTCAGG  R:CTGCGTGACAGTTCATCAAAACC | 57.5 |
| scaffold78_8903 | JN902044 | F:GTCTCAGATGAGGCCCGAAA  R:CTCCTCCTTAGCAGCTCTCC | 57.5 |
| scaffold519_24943 | JN902045 | F:GGCTCACATTCACATGCAGAG  R:CATTGTTCCGCAGATTAGGAGC | 57.5 |
| scaffold380_18591 | JN902046 | F:TGGAGATCACAAGAACCTACTGGT  R:GGAGCATGTTGTCGTAGCG | 57.5 |
| scaffold203_10567 | JN902047 | F:CATCAGCATCCAGATCGCAC  R:AGGCTGAACCTTCTCTGTAGC | 57.5 |
| scaffold145_6259 | JN902048 | F:TGTGATGGTTAAACTGCTCCGT  R:GGCTTACTCGAGGTTAACTGCA | 57.5 |
| scaffold347_17272 | JN902049 | F:GCTGAAACTCTGCTTACACCTGA  R:TCATGCTTGCTCGATGTGGAA | 57.5 |
| scaffold244_12590 | JN902050 | F:CATCGGGAATTTCACTGTGGTTG  R:GTGAGACAAAGCTCTCAGGCT | 57.5 |
| scaffold261_13086 | JN902051 | F:ACCTTTTGCAAAGTAAAGGCCC  R:TGAGTTTGTTGGACGGGTTTCA | 57.5 |
| scaffold261_13063 | JN902052 | F:CAGAAACTGTGTGACAGCGAC  R:CAGAGACGATGGGCACTATACC | 57.5 |
| scaffold781_27375 | JN902053 | F:GAGAGCTAATCACTGTCTCTGGC  R:CACTTCTGCAACACGATAGCC | 57.5 |
| scaffold724_4569 | JN902054 | F:CATGTCCCTGTCTGCACG  R:CTGTGGTGGTATTATCAGCTCCA | 57.5 |
| scaffold678_4445 | JN902055 | F:TCATTTCTAATGTAATGGCACACTG  R:ACATGGATGAAGATCCAGACACG | 57.5 |
| scaffold480_2612 | JN902056 | F:AGTCTTGTTAGGCTGCTCCC  R:AGTCACACTTACCACAGAGTTCC | 57.5 |
| scaffold648_3978 | JN902057 | F:CGAAGGTCAGATATCCAGGTCATG  R:TGGGTGTCTAGAACAGGGC | 57.5 |
| scaffold297_1038 | JN902058 | F:AGGAGCCTCGCACGTATC  R:TACAGACAGACGACGGCAC | 57.5 |
| scaffold410_1887 | JN902059 | F:GGTAAAAGGTTTAGCCAGCAGC  R:GGTTCTTGTCTCCGCACAATG | 57.5 |
| scaffold418_1962 | JN902060 | F:TGAAGAGAGATTGAGGCAGAGTGA  R:GTCTCACAAACCGTCTCTCACAAA | 57.5 |
| scaffold221_676 | JN902061 | F:TCTTTAGGAAACAAGGTCCACGG  R:TAATGAACGACCGAGGAAGGATTA | 57.5 |
| scaffold652_3843 | JN902062 | F:AGTTGCCATTTCCACCACATG  R:CAGACACAACAGTGGAGCAC | 57.5 |
| scaffold709_4343 | JN902063 | F:GCTCCAGTTTGACTTATGGCC  R:GAGTGAACTATAAGCTGCTGCCA | 57.5 |
| scaffold660_4030 | JN902064 | F:TCCTGACACCGAGTTGTTCC  R:TGTGCACAAGACAAGTTGCTG | 57.5 |
| scaffold719_4555 | JN902065 | F:TAGTGACGACACGAGCAGC  R:GCTCGAACTGTGCCATCAG | 57.5 |
| scaffold330_1300 | JN902066 | F:CTGATCAGAGACGAACGACCA  R:GTCCAGCTACTAAAAGAGGGACG | 57.5 |
| scaffold779_4855 | JN902067 | F:GAACAGTAGTGGGCAACAGG  R:CGAGTGTGATTATCTGACGGGAG | 57.5 |
| scaffold232_935 | JN902068 | F:GAGTCTGAGACATCCAGTTGGTC  R:AGCTAGTGGTGTGTGAGTCTG | 57.5 |
| scaffold800_5109 | JN902069 | F:GAGTCTGAGACATCCAGTTGGTC  R:AGCTAGTGGTGTGTGAGTCTG | 57.5 |
| scaffold853_5017 | JN902070 | F:AAAAGAGAGACCTGCTCCAGG  R:TGGTGTGTTTCCAGAGAGGG | 57.5 |
| scaffold993_5355 | JN902071 | F:GCTCCGTTGGGTTCAGAC  R:TGTCAGTAGGGGTTCGTGTC | 57.5 |
| scaffold437_21639 | JN902072 | F:GTGCAGAATGACACCGACAG  R:GTCCAGGAGTTTGTCATGTTGC | 57.5 |
| scaffold44_1181 | JN902073 | F:TGTACTGTGAGTTCGTGTGCTTGT  R:TTACTCTCATGTGACCCAGTCGTC | 57.5 |
| scaffold457_23641 | JN902074 | F:TCAAACACCGTTGGGATGTACTC  R:CTGCGAAGGTAAACTCAGGTCA | 57.5 |
| scaffold634_3794 | JN902075 | F:GAGTCGCTCAGTCTCTTGACAG  R:ATTCTGTATGCACCTGGAGGAC | 57.5 |
| scaffold617_3510 | JN902076 | F:ACTGCAACATGACACTTCACCACT  R:GTCGTTATACCAACGCTGCTCTTT | 57.5 |
| scaffold185_597 | JN902077 | F:AGTCCATGTCGAGCAGGTAAAC  R:ACTCAGTGGATTGAAGTTGGGAG | 57.5 |
| scaffold580_3242 | JN902078 | F:TGTTTGCAGTGTTGCCGTC  R:GAGACCTGGCACTTTGTTTGG | 57.5 |
| scaffold631_3692 | JN902079 | F:GTGCTTTCAATCAACCTCAGCTC  R:AGCAGTAGAGGGCCATGAG | 57.5 |
| scaffold564_3012 | JN902080 | F:CCTGGAGACCGAACAGTAGAGAGA  R:CACACTGAGACTAATTGGCCCTCT | 57.5 |
| scaffold435_2112 | JN902081 | F:CCTAAATCAAGCTGACTGCGC  R:TTGACCTGCTAGTTACTGTCGC | 57.5 |
| scaffold741_4648 | JN902082 | F:CAGGACGTTCATGATGTTGGTG  R:TCTGCAAATCGGTGTGAATGC | 57.5 |
| scaffold314_1152 | JN902083 | F:GTTTCAGCGTGTCCATTAGACGA  R:CTGCATTTCACTGTGACCATCTG | 57.5 |
| scaffold568_3025 | JN902084 | F:ATTCAGACTGAGCCTTGGTCC  R:GACTTCATTTAGCTGGGTTGCAC | 57.5 |
| scaffold639_3779 | JN902085 | F:TGCCTGAGGTTACAAACAACTCAA  R:TGCATGAATATGAGCTTGTCAATG | 57.5 |
| scaffold588_3265 | JN902086 | F:ACAGACAGTGCTGACAGTTCTG  R:AGCAGTCATATGGACATAAGGGTG | 57.5 |
| poli1258TUF | EF113014 | F:GAACACACATGCTTAATGGGATGG  R:GTGCACCTGTGTGTGTATCAC | 57.5 |
| poli847TUF | EF112802 | F:CCACATAACGAGCCACTGTGATA  R:ACTCCTCCTTGATGCGGTC | 57.5 |
| poli1853TUF | AB459133 | F:AGTCTGGAACTTTTGAGTGGGAG  R:CAGGGTAGAGCCCCATCAT | 57.5 |
| poli1502TUF | DQ889062 | F:TTTTCAACGTCGCCCAATCC  R:CAACGTGACAGAGGTGTCATCA | 57.5 |
| poli1410TUF | DQ888975 | F:TGATCTGCTCCACAGTTTAGCTG  R:CGTTCTCAGGCTGTTTGAAGG | 57.5 |
| poli1432TUF | DQ888997 | F:CCCATTGTTGTCCCAGCTC  R:AGAACAGGGGACTGAAGGTG | 57.5 |
| poli151TUF | AB459425 | F:AATACTGCGAGAGTGTACTGCG  R:GTGCGTTTGTACAATAGTGGAACC | 57.5 |
| poli473TUF | EF112671 | F:CCAGTTTGCTCTGCAGGATG  R:CTGTGATTACGCCAGGTGC | 57.5 |
| poli466TUF | EF112665 | F:CAGTGACTCTGTGAGTGGACAA  R:ACCAGGAAGTGTGCCATATCTC | 57.5 |
| poli1323TUF | EF113069 | F:GGTCCAACGACCAAGGAGA  R:GATCTACAGCTTGTACCCCTCATG | 57.5 |
| poli2030TUF | AB459266 | F:TTCCTTCCATTAGCAGCGGAAT  R:GGATAGAAAAGCATGACAGCGC | 57.5 |
| poli964TUF | EF112890 | F:CGGTGGATTTGGATCAGCAC  R:TCTTTATCTTCGGCCACAGTCATG | 57.5 |
| poli1352TUF | DQ888926 | F:TCGTCAGTAGCAGGATTTGTTGG  R:GATAGTCCCCTTATTCGCGTGAG | 57.5 |
| poli1188TUF | EF112959 | F:GCACTTCATTTCTCAGCATCGTG  R:CCTTCCAAGCCAAGGTGATG | 57.5 |
| poli16-37TUF | AB459364 | F:ACAACTCCCATCAACTCAGGG  R:ACCGAGCGAATAAAGACAGCTTAG | 57.5 |
| poli845TUF | EF112800 | F:CTCTTAGTTCCCAGAGTTCCCG  R:CCGAGGTTTGAGAGATAGTCCC | 57.5 |
| poli1216TUF | EF112981 | F:CAATTCAGAGCAGATGTGAGTGC  R:TGGCTGGAATCGATAGTCACG | 57.5 |
| poli1259TUF | EF113015 | F:GCCCTCACTGAGTCATCCA  R:CAACTACAACTCACCTCAGTGCT | 57.5 |
| poli1387TUF | DQ888955 | F:TCACAGGTGCTCATTGAGAGG  R:GAGGTGAGTGAATGAATCGTGGA | 57.5 |
| poli475TUF | EF112673 | F:CTGCACCAACGTGTGTGTTAG  R:TATCCAATCAGCTGTCAGGGAAC | 57.5 |
| poli1321TUF | EF113068 | F:TCCAGGTTCAGGTCAATCTCAC  R:TGATATCAAACAACCGAGAGCGAG | 57.5 |
| poli1514TUF | DQ889073 | F:ATGCTGGTGTAGGGTTCCTC  R:TGTACAACTGACACAGTTGGAGTC | 57.5 |
| poli1279TUF | EF113032 | F:GATGCTAGCTCATCACAGGCT  R:CAGTAGAAACACCTGTGTGGTCA | 57.5 |
| poli798TUF | EF112758 | F:AACGGCAGGACTGAAGTACAG  R:GCTGAAAAGGATGATGTGGCAG | 57.5 |
| poli1289TUF | EF113040 | F:ACCATAAACGATCATCTGGGACC  R:CTGTAAGCCAGAGGCCAAAC | 57.5 |
| poli1156TUF | EF112938 | F:ATGAAAGCACCAGGAGGAAGAG  R:ATAAGCCATCCCTGCTGTCTC | 57.5 |
| poli1968TUF | AB459227 | F:CCATTTGTCAGCAGTCTGAAGG  R:TCCGGGATTTCAGACTCAGC | 57.5 |
| poli1431TUF | DQ888996 | F:ACTCGAGAGCTTTGACAACATCTG  R:GCGTGGTGCAGGTCTAATG | 57.5 |
| poli1023TUF | AB458915 | F:ACTGGGACACTGTTCCTGAG  R:GTCACACACATTTCTGCAGTCC | 57.5 |
| poli1232TUF | EF112992 | F:TGGCCTTAAACTTGGACTAGCTTC  R:AGCTCAGATGGGCTTCAGAC | 57.5 |
| poli1762TUF | AB459053 | F:TGCCGCAGACTTGTACATACTG  R:TGTAAGACAGTGGCACCCAAA | 57.5 |
| poli1418TUF | DQ888983 | F:ATAGATTCTCCATGGCAACGGG  R:CAGCTCACAATGAAAATCACCACG | 57.5 |
| poli1578TUF | AB458966 | F:AGGGAGTGGAGCTCTTAATGC  R:CTGTGGTGGACACTGCAAC | 57.5 |
| poli749TUF | EF112732 | F:TCAGCATGTAACCACGCTCTAG  R:CCACAACGCAGAAGGTCAAG | 57.5 |
| poli1413TUF | DQ888978 | F:GTCAAATCAACTGTCACACGCC  R:CACCTGCAGTATCTCTCCTGTAC | 57.5 |
| poli1981TUF | AB459238 | F:GCAATTGAAGCCCTCCACATAC  R:CTAGCAACAAGGACCCCTCA | 57.5 |
| poli1136TUF | EF112927 | F:CTCTTCTTCACAACCATCGCG  R:CGTGTGTGTAGCATGGAGC | 57.5 |
